# CONET: copy number event tree model of evolutionary tumor history for single-cell data

**DOI:** 10.1186/s13059-022-02693-z

**Published:** 2022-06-09

**Authors:** Magda Markowska, Tomasz Cąkała, BłaŻej Miasojedow, Bogac Aybey, Dilafruz Juraeva, Johanna Mazur, Edith Ross, Eike Staub, Ewa Szczurek

**Affiliations:** 1 University of Warsaw, Faculty of Mathematics, Informatics and Mechanics, Banacha 2, Warsaw, Poland; 2 Medical University of Warsaw, Postgraduate School of Molecular Medicine, Ks. Trojdena 2a Street, Warsaw, Poland; 3 Merck Healthcare KGaA, Translational Medicine, Oncology Bioinformatics, Frankfurter Str. 250, Darmstadt, 64293 Germany

**Keywords:** Copy number alterations, Tumor evolution, Single-cell sequencing, Probabilistic model, MCMC sampling

## Abstract

Copy number alterations constitute important phenomena in tumor evolution. Whole genome single-cell sequencing gives insight into copy number profiles of individual cells, but is highly noisy. Here, we propose CONET, a probabilistic model for joint inference of the evolutionary tree on copy number events and copy number calling. CONET employs an efficient, regularized MCMC procedure to search the space of possible model structures and parameters. We introduce a range of model priors and penalties for efficient regularization. CONET reveals copy number evolution in two breast cancer samples, and outperforms other methods in tree reconstruction, breakpoint identification and copy number calling.

**Supplementary Information:**

The online version contains supplementary material available at (10.1186/s13059-022-02693-z).

## Background

Elucidating tumor evolutionary history is pivotal to a better understanding of carcinogenesis and helps developing new cancer treatments. Copy number (CN) alterations (CNAs) are ubiquitous in the genomes of tumors across all types of cancer [1–3] and constitute the most common alteration types associated with tumor hypermutability [4]. CN events, corresponding to occurrences of CNAs in the evolutionary history of a tumor cell population, play an important role as drivers of tumor evolution. In particular, amplifications can activate so called oncogenes, promoting increased growth and other hallmarks of cancer [5], while deletions can disable tumor suppressors [1, 2].

Copy number calling and reconstruction of CN evolution from bulk sequencing data is notoriously difficult, as only the aggregated signal per region is observed [6–8]. In particular, bulk tumor samples are mixtures of thousands or millions of cells, some of which are normal cells, while others come from distinct tumor clones and differ by their CN profiles. Methods for approaching CN calling and evolution reconstruction from bulk data either focus on finding the mixture of different CN profiles and their unknown proportions in bulk samples [9–23], or the identification of appropriate measures of phylogenetic distance between the bulk CNA profiles [24–28].

Currently, the technology of single-cell DNA sequencing (scDNA-seq) revolutionizes the analysis of tumor cell populations and gives valuable insights into tumor evolution [29]. In contrast to bulk sequencing, scDNA-seq offers the measurement of the genomic sequence at the level of individual cells. As such, it paves the way for the identification of genomic alterations in single genomes, and for the reconstruction of their evolutionary relationships [30].

**Fig. 1:**
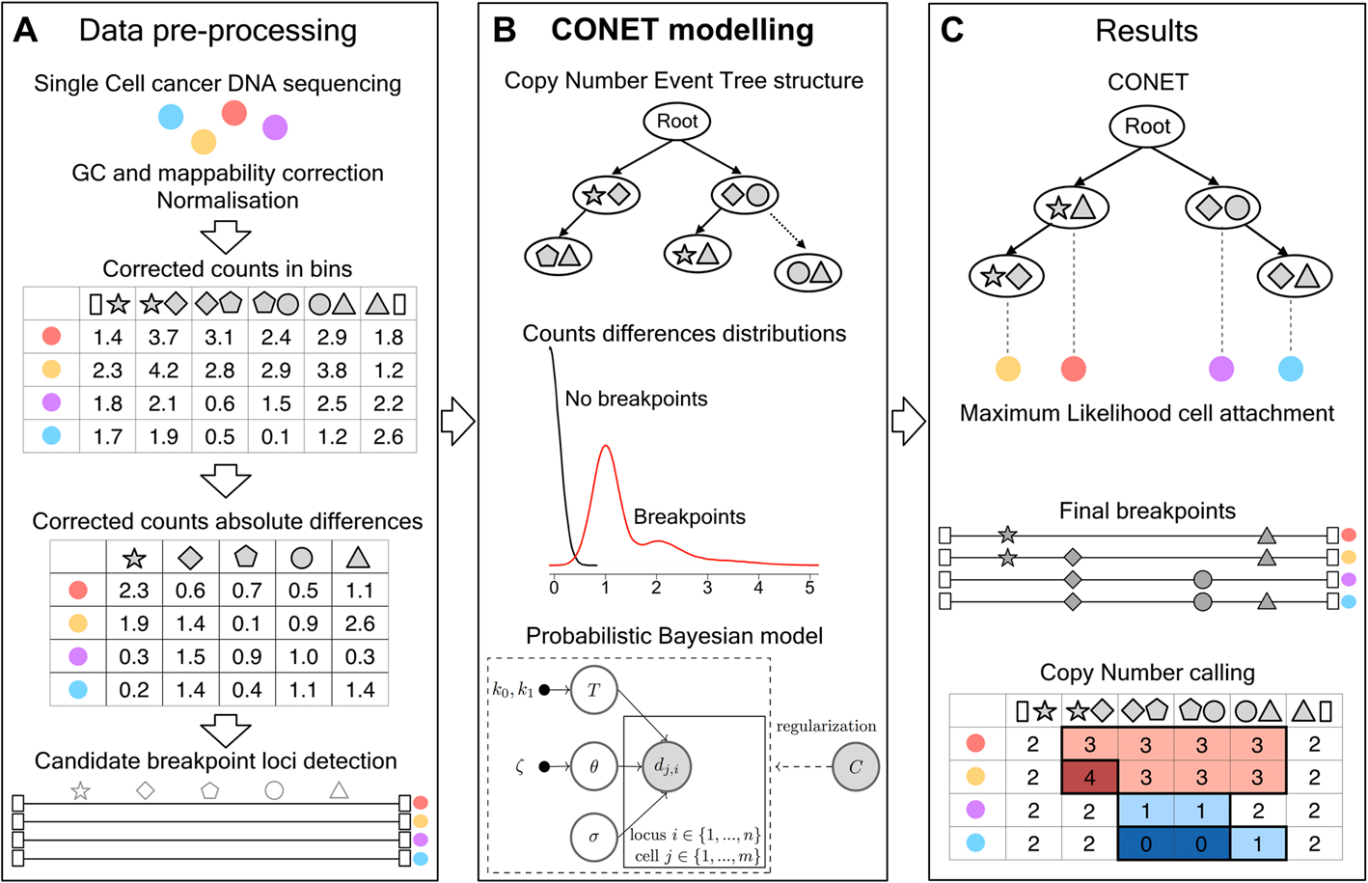
Joint inference procedure of CONET and integer CNs. **A** CONET input consists of the corrected counts in bins (per-bin data) and set of candidate breakpoint loci; the data needs to be pre-processed with GC and mappability correction and normalization methods best suited for a given scDNA-seq technology. **B** CONET is a rooted tree over nodes that are CN events, modeled as pairs of breakpoints, and edges that correspond to their partial order of occurrence. The main CONET inference procedure is an efficient MCMC sampling scheme, searching the space of possible CONET structures and model parameters, using corrected count absolute differences (per-breakpoint data) for likelihood calculation and per-bin data for model regularization. **C** In the post-processing steps, the cells are attached to the final CONET nodes using Maximum Likelihood. The inferred breakpoint history in each single cell, together with per-bin data, are used to determine the CN profiles. The latter is performed by grouping bins that underwent the same evolutionary history according to the tree and cell assignment (marked with widened boarders in the Copy Number calling matrix) and assigning them a CN equal to a rounded median across their corrected counts (marked with colors)

Several approaches have already been proposed for modeling tumor evolution of single nucleotide variants (SNVs) from scDNA-seq data [31–35] or jointly from bulk and scDNA-seq [36–38]. In contrast, modeling CN evolution of tumors from scDNA-seq data is still in its infancy. The most important challenges that need to be addressed include i) noise and low coverage uniformity in scDNA-seq data, and ii) the fact that CN events may overlap, and are thus not independent. In particular, a CNA acquired in a certain early CN event in the course of tumor evolution, may not be observable as a consistent CN segment in a cell sampled from the tumor population, as it may have been further altered by later CN events (for a didactic example, refer to Fig. 1C.) Consequently, the often-made infinite sites assumption [39, 40], which states that every genomic position is altered at most once in the evolutionary history of a population, does not hold [41–43], and traditional phylogenetic approaches operating on SNVs are not applicable [6].

A number of scDNA-seq high-throughput techniques, such as DOP-PCR [44–46] and C-PCR-L [47] achieve relatively even coverage distribution along the whole genome of the sequenced cells [6, 48]. DLP and DLP+ have been proposed as technologies omitting the pre-amplification step, and thus avoiding the amplification bias [49, 50]. Recently, a method called acoustic cell tagmentation (ACT) was developed, which performs high-throughput scDNA-seq at single-molecule resolution by combining fluorescence-activated cell sorting of single nuclei, tagmentation and acoustic liquid transfer technology [51]. These techniques open up a new avenue to CN calling in single tumor cells. After appropriate pre-processing of the data, including GC content bias correction and normalization, approaches such as circular binary segmentation (CBS) [52, 53] and MergeLevels [54] originally applied to array comparative genomic hybridization data, are commonly used for CN calling in scDNA-seq datasets [49, 51]. Specialized CN calling tools for scDNA-seq were proposed, including HMMCopy [55], Aneufinder [56], Gingko [57], Scope [58], and CHISEL [59]. Existing CN calling approaches, however, suffer from high false positive rates [48, 60]. In particular, identifying the locations of the borders between genomic segments with different CN, which are referred to as *breakpoints*, is largely hindered by the inherent noise in the scDNA-seq data. Finally, none of these CN calling approaches account for the fact that the observed CN data are generated by an evolutionary process.

Recent approaches analyzing CN changes in single cells from an evolutionary perspective aim at improving breakpoint and CN calling from scDNA-seq based on breakpoint trees (sitka; preprint) [61], modeling cell lineages based on the similarities of CN profiles between single cells and identifying events carrying fitness advantage (MEDALT) [62], analyzing large events such as whole genome duplications (MEDICC2; preprint)) [63], or inferring CN evolution and CN states from the binned read counts (SCICoNE; preprint) [64].

Here, we propose a novel approach for Copy Number Event Tree (CONET) inference and CN calling (Fig. 1). The model jointly infers the structure of an evolutionary tree on CN events and CN profiles of the cells, gaining statistical power in both tasks. The nodes of the evolutionary tree are CN events, which are allowed to overlap. CONET fully exploits the signal in scDNA-seq data. First, it models the *per-breakpoint* data, given by the difference of the counts at the potential breakpoint loci. The higher the absolute value of the difference, the more evidence for the presence of that breakpoint in the cell. Second, it utilizes the *per-bin* data, given by the sequencing counts in genomic bins, to regularize the model and perform CN calling. Joint inference of tumor phylogeny from single cells and CN calling from scDNA-seq data, exploiting both the per-bin and the per-breakpoint signals, can be expected to result in better evolutionary history reconstruction and more accurate CN calls.

CONET outperforms other compared methods in tree reconstruction, breakpoint identification and copy number calling, both for simulated data and in application to data for 260 cells from xenograft breast cancer sample. Taken together, the proposed approach is a step towards a better understanding of CN evolution in cancer.

CONET implementation is available at https://github.com/szczurek-lab/CONET.

## Results

### Model overview

We propose a joint procedure that efficiently infers the history of CN events occurring in single cells in the tumor tissue, identifies the presence of breakpoints and estimates the integer CNs in regions defined by these breakpoints in each cell from scDNA-seq data (Fig. 1). In the first step (Fig. 1A), we pre-process the per-bin scDNA-seq data from tumor sample, by correcting for GC content and mappability, and normalizing to a given basal ploidy. Most commonly, the basal ploidy can be assumed to be two, which is the ploidy of a healthy cell. For some tumor samples, however, it may be known that the evolutionary process has started by a genome-wide event, such as genome duplication, changing the basal ploidy to a different number.

To specify the per-breakpoint input data to the CONET model, we calculate the differences between the corrected counts in adjacent bins for each cell, arriving at the corrected count absolute difference matrix (Methods). Finally, we establish the set of candidate breakpoint loci. Here, both the data pre-processing and candidate breakpoint loci, are found using existing methods for copy number calling, such as HMMCopy [55] or copynumber [65], followed by MergeLevels [54]. (“Real data pre-processing” section). In general, the method used should be best suited for a given scDNA-seq technology and its choice belongs to the user.

The per-bin and per-breakpoint data constitute the input to CONET, a novel probabilistic model for inferring tumor evolution on single-cell CN events (Fig. 1B). The structure of CONET is defined by a rooted tree, with the root representing a cell with a given basal ploidy, and each remaining node corresponding to a CN event, interpreted as a CN change that introduces the occurrence of two breakpoints (start and end loci of the event, graphically represented by different shapes in Fig. 1). The events from different nodes (different CN events) are allowed to overlap. The candidate breakpoint loci are allowed to repeat as a start or end loci of different events. In this way, our model follows the finite sites assumption. In contrast to the infinite sites assumption, which is often made by models operating on SNVs, the finite sites assumption allows a genomic position to be changed more than once in evolutionary history and is much more realistic for CN events, as the same breakpoint loci with increased genomic instability were observed to be re-used in tumor evolution [66]. The evolutionary tree structure, together with the cells’ attachment to its vertices, defines the CN events history of each cell and consequently, the set of breakpoints that should be observed within each cell sequence.

We model the per-breakpoint data assuming that the corrected count absolute differences follow different distributions for breakpoint and no-breakpoint loci, i.e. for loci with and without CN change in adjacent bins, respectively. Specifically, the corrected count absolute differences in no-breakpoint loci are expected to be close to zero. In contrast, for breakpoint loci, the corrected count absolute differences should be close to or greater than one (depending on the magnitude of CN change). For real data generated using two different scDNA-seq technologies, DLP and ACT, the assumed data distributions fit the data perfectly (Additional file 1: Fig. S1). The parameters of those two distributions constitute the set of model parameters. We devise an efficient Markov Chain Monte Carlo (MCMC) sampling scheme that allows to jointly search the vast space of possible CONETs and model parameters. To regularize the model performance for noisy biological data, we employ a set of priors that help to obtain reliable results with desirable tree complexity (Methods). Specifically, we define a *tree structure prior*, controlling the size of the inferred trees, as well as an *attachment prior*, allowing to prioritize trees that attach cells to nodes with a history consisting of shorter event. The latter is motivated by the fact that shorter events are generally more likely to occur than long ones [66]. We also introduce a *count discrepancy penalty* that accounts for the per-bin data and penalizes the model for the inconsistency of corrected counts in bins with the same CN change history. Intuitively, the count discrepancy penalty corrects discrepancies between the observed per-bin data and the inferred CNs across cells.

Having the final CONET, we use Maximum Likelihood to attach each of the single cells to one of the tree vertices (Fig. 1C). To perform CN calling for these cells, we utilize the fact that the CN change history for each bin in each cell can be derived from the path from the vertex to which the cell is attached in the CONET tree to its root. Specifically, for each bin in each cell, we identify the set of CN events on this path that contain the bin between their start and end. By performing this task for all cells, we define clusters of bins that underwent exactly the same CN changes and thus should have the same integer CN. We calculate the inferred CN in each bin in each cell by averaging and rounding the corrected counts in each cluster. For all the bins not included in any CN event, we assume a basal ploidy, thus arriving at the estimated integer CN matrix (Fig. 1D).

### Performance of CONET in comparison to other methods on simulated data

To demonstrate the advantage of CONET over other methods, in a setting where the ground truth is known, we conduct the evaluation on simulated data. To this end, we devise and use a generative model that samples tree of a given size with attached predefined numbers of cells, outputting per-bin data in the form of corrected counts matrices (“Simulations for comparative evaluation of CONET on both per-bin and per-breakpoint data” section). Along CONET, we employ SCICoNE [64], as well as HMMCopy [55], circular binary segmentation [52], followed by MergeLevels [54] (referred to as CBS+MergeLevels) and simple rounding of corrected counts to nearest integer (referred to as CC-rounding). HMMCopy and CBS+MergeLevels are CN calling tools developed specifically for scDNA-seq data and together with CC-rounding are applied for CN calling, while SCICoNE is applied to both tree reconstruction and CN calling. For these simulated data, CONET is run using tree structure prior and attachment prior (see “Priors” section), and utilizes the count discrepancy penalty (“Definition of the count discrepancy penalty” section), which regularizes CONET based on the per-bin data. For all runs, the regularization parameters of CONET are fixed to the same values (for used parameter values see Additional file 1: Section S1). The run settings for the other methods were fixed to defaults and are given in Additional file 1: Section S2.

Each evaluation scenario is described by three parameters—the size of the real tree (equal to 20 or 40), the number of cells that are randomly assigned to tree vertices in the simulation (with values 200 or 1000), and, finally, the corrected counts distributions that are used for the generation of the corrected counts matrices. We use two distributions settings, which differ by the level of noise involved. Additionally, SCICoNE and CONET are run in different versions. SCICoNE (known) and CONET (known) are given true candidate breakpoint loci set as input. If the candidate breakpoint set is not given, SCICoNE uses a build-in approach for identifying candidate breakpoints, while CONET (HMMCopy) uses candidate breakpoint loci found by HMMCopy (identified as breakpoint in at least one cell), and CONET (CBS+MergeLevels) chooses those loci which are identified as breakpoints in at least 5 cells by CBS+MergeLevels.

For each of the scenarios described above (tree size, number of cells, distributions setting) we generate 50 random datasets. For each generated dataset, we run each method (CONET, SCICoNE, HMMCopy, CBS+MergeLevels, CC-rounding) 2 times (each time with a different seed). We then compare the inferred data to the ground truth from the simulated data.

Each CONET inference is run with 5·10^5^ steps for joint tree and parameter inference followed by 10^6^ steps for tree inference. The average running time of one CONET inference procedure ranges from less than 20 minutes in the least computationally demanding scenario (tree size 20 with 200 cells) to less than 120 min in the most demanding scenario (tree size 40 with 1000 cells), on a system equipped with AMD Ryzen Threadripper 3990X 64-Core CPU and 128 GB RAM, using 5 threads.

To assess the quality of inference results we use eight scores – *event precision*, *event sensitivity*, *edge sensitivity*, *edge precision* as well as *CN-RMSE*, *false positive rate*, *false negative rate*, and *symmetric distance* (see “Model evaluation metrics for simulated data” section for definitions). The first four scores assess the quality of the inferred CN event tree and as such can only be used to evaluate output of CONET and SCICoNE. Event sensitivity and event precision quantify the similarity of the inferred tree’s vertex set to that of the real tree, with their larger values indicating higher similarity. Similarly, edge sensitivity and edge precision quantify the similarity of the inferred tree’s edge set to that of the real tree. The last four scores assess the quality of breakpoint identification and CN calling procedure, with lower values indicating better results. False positive rate is a fraction of inferred breakpoints that are not present in the cells according to the true tree and cell attachment. False negative rate is a fraction of breakpoints that should be present in cells according to the true tree and cell attachment, but are not according to inference results. Symmetric distance measures the average number of incorrectly inferred breakpoints per cell. CN-RMSE is a root mean squared error between real and inferred integer CN matrix.

#### Performance of tree inference

**Fig. 2:**
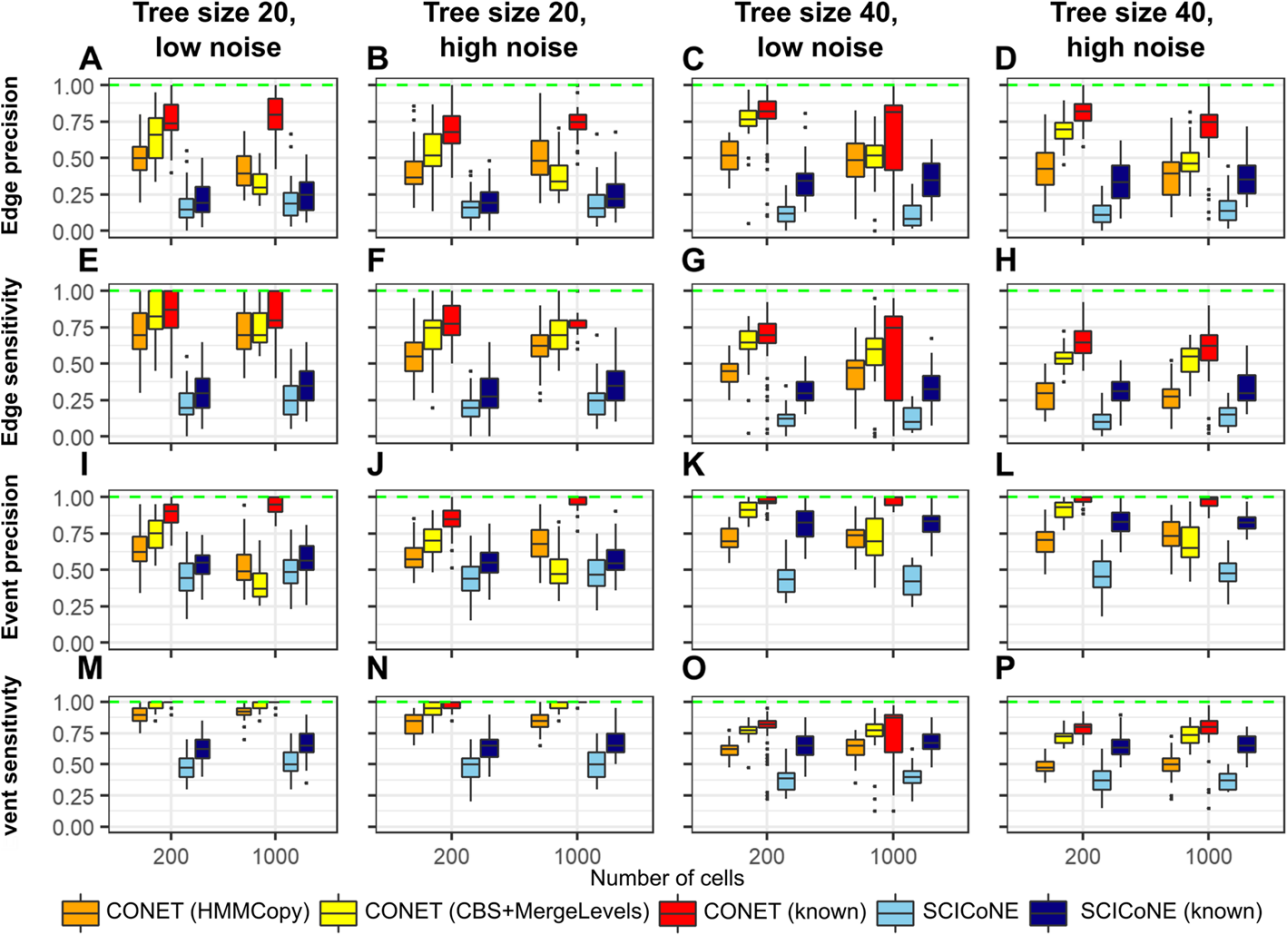
Comparative assessment of the tree structure inference for simulated data tests results. **A**–**D** Distribution of edge precision (*y*-axis) depending on the cell number (*x*-axis) for all simulation scenarios: smaller tree size (first and second column) versus larger tree size (third and fourth column), low noise (first and third column) versus high noise (second and fourth column). **E**–**H** Distribution of edge sensitivity (*y*-axis) depending on the cell number (*x*-axis) for all scenarios. **I**–**L** Distribution of event precision (*y*-axis) depending on the cell number (*x*-axis) for all scenarios. **M**–**P** Distribution of event sensitivity (*y*-axis) depending on the cell number (*x*-axis) for all scenarios. Optimal values of the presented quality measures are marked with a green dashed line in each plot

In terms of tree inference, CONET clearly outperforms SCICoNE, in both versions (with known and unknown candidate breakpoint loci; Fig. 2). Specifically, the best performance results according to all measures are for CONET (known), with median edge precision in (0.68, 0.82), median edge sensitivity in (0.62,0.87), median event precision in (0.84, 1), and median event sensitivity in (0.8, 1). The second best performance is observed for CONET (CBS+MergeLevels)). The worst performance results are obtained by SCICoNE without known breakpoint candidates loci, with median edge precision and sensitivity in (0.11, 0.25), median event precision and sensitivity in (0.37, 0.5). In all simulation scenarios CONET performs better than SCICoNE in pairwise (known vs known, or unknown vs unknown candidate breakpoint loci) comparisons. Moreover, for 21 out of all 32 scenarios and quality measures, CONET (HMMCopy) outperforms SCICoNE (known), while CONET (CBS+MergeLevels) outperforms SCICoNE (known) for 28 scenarios and measures. The advantage of CONET over SCICoNE may be due to the fact that CONET benefits from both the per-bin and per-breakpoint data, while SCICoNE operates only on the per-bin data, as well as from the fact that SCICoNE learns a tree labeled also by CN, and thus traverses a much larger space of possible trees during inference. Both CONET and SCICoNE prove to be robust to noise. Indeed, for both methods the evaluation measures, if at all, decrease only slightly for high noise versus the low noise setting. Larger trees are more difficult to infer for both methods. While the median edge precision decreases only slightly (Fig. 2A–D) and median event precision (Fig. 2I–L) does not change or even increases for larger trees, compared to the smaller trees, median edge sensitivity (Fig. 2E–H) and median event sensitivity (Fig. 2M–P) drop for larger trees. For example, the lowest median event sensitivity obtained by CONET for smaller trees is around 0.8 in the high noise setting, and it decreases to around 0.5 for larger trees. In case of SCICoNE, the lowest median event sensitivity for smaller trees is around 0.5, and it decreases to around 0.37 for larger trees. All in all, the obtained performance results illustrate superior performance of CONET in tree reconstruction.

#### Performance of breakpoint detection

**Fig. 3:**
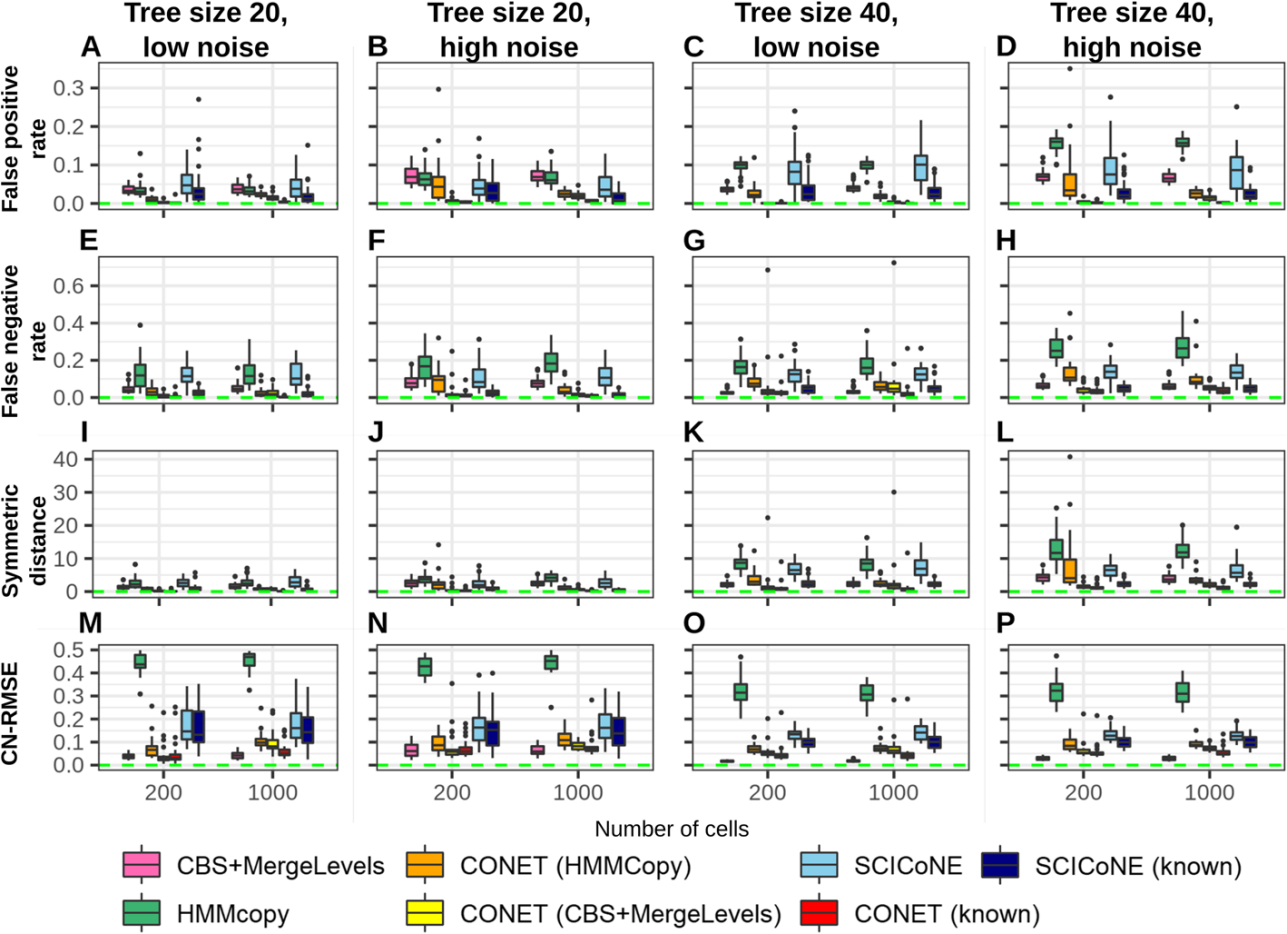
Comparative assessment of breakpoints inference and CN calling for simulated data tests results. **A**–**D** Distribution of false positive rate (*y*-axis) as a function of cell count (*x*-axis) for all simulation scenarios: smaller tree size (first and second column) versus larger tree size (third and fourth column), low noise (first and third column) versus high noise (second and fourth column). **E**–**H** Distribution of false negative rat (*y*-axis) as a function of cell count (*x*-axis) for all scenarios. **I**–**L** Distribution of symmetric distance score between inferred and real breakpoints (*y*-axis) as a function of cell count (*y*-axis) for all scenarios. **M**–**P** Distribution of CN-RMSE (*y*-axis) depending on the cell number (*x*-axis) for all scenarios. Optimal values of the presented quality measures are marked with a green dashed line in each plot

Figure 3A–L illustrates superior CONET performance in terms of breakpoint identification (detection of presence of breakpoints across cells for a set of candidate breakpoint loci). CONET (known) outperfoms other methods in 21 out of 24 scenarios. CONET (HMMCopy), CONET (CBS+MergeLevels), CONET (known), and SCICoNE (known) show lower false positive rate than HMMCopy and CBS+MergeLevels (Fig. 3A–D). This is possibly due to the fact that in contrast to CONET and SCICoNE, neither HMMCopy nor CBS+Mergelevels benefit from the inferred phylogenetic relationships between the analyzed cells and have difficulty distinguishing noise from real CN changes. Large false positive rates of SCICoNE without known breakpoint candidates loci can be due to problems with identification of the correct candidate breakpoints and not tree inference and CN calling. In terms of false negative rate and symmetric difference, CONET (known), CONET (CBS+MergeLevels) and SCICoNE (known) outperform HMMCopy and CBS+Mergelevels. The latter, however, shows consistently better performance per these two measures than SCICoNE without known breakpoint loci in all simulation settings, and tends to outperform CONET (HMMCopy) for larger trees. This again emphasizes the importance of identifying a good set of candidate breakpoint loci. Generally, the breakpoint identification is a bit harder for all methods when larger trees are simulated. CONET (known) shows the best, while HMMCopy the worst breakpoint identification performance out of all compared methods. Specifically, for the best performing CONET with known breakpoints, median false positive rate is around 0 for all simulation scenarios, median false negative rate is in (0,0.03), and median symmetric difference is around 1. This means that on average every cell has only a single missed or wrongly inferred breakpoint. Across these quality measures and experiment scenarios, CONET (CBS+MergeLevels) peforms similarly well to CONET (known). For the worst performing HMMCopy, the obtained median false positive rate is in (0.03,0.16), median false negative rate is in (0.1,0.25), and median symmetric difference is in (2,11). Notably, all compared methods are run using default settings. Possibly, the observed performance of HMMCopy could be improved by calibrating its parameters.

In comparison to CONET and other methods, the naive approach of CC-rounding performs dramatically poorly in terms of breakpoint detection (Additional file 1: Fig. S2). Results for CC-rounding are excluded from Fig. 3, as they are out of scale and render the differences between the other approaches hardly noticeable. The false positive rate for CC-rounding exceeds 0.6 for each scenario, and its median false positive rate is at least 0.74. In the most difficult scenario, for large trees and high noise, median false positive rate reaches 0.97. The false negative rate is small (median values between 0.01 and 0.035). Finally symmetric difference ranges between 100 and 1250. This indicates that naive CC-rounding mistakes noise for CN events.

#### Performance of CN calling

In terms of CN calling, the CN-RMSE measure (Fig. 3M–P), is minimized by CBS+Mergelevels, which obtains values close to zero. CONET (known) and CONET (CBS+Mergelevels) perform similarly to each other in terms of CN-RMSE and tend to have a bit larger values than CBS+Mergelevels but lower than CONET (HMMCopy) and SCICoNE in both versions. We observe worse performance for SCICoNE when the candidate breakpoint loci are not known. Similarly to tree inference, both when the breakpoints are known and when they are not given, CONET outperforms SCICoNE in most simulated scenarios. HMMCopy performs the worst by a large margin, reaching as high values as 0.54, compared to max median of 0.17 for the other methods, across all simulated scenarios. Again, this might be improved with calibrating the parameter settings of the model.

Median CN-RMSE for the naive CC-rounding exceeds 0.1 for scenarios with low noise, and is larger than 0.24 for higher noise (Additional file 1: Fig. S2). These results confirm that more sophisticated approaches are required to avoid over-fitting to noise in the data and assure high quality breakpoint inference and CN calling.

All in all, the obtained results illustrate superior performance of CONET in tree reconstruction, breakpoint identification and CN calling in comparison to other methods.

### Application to scDNA-seq data of SA501X3F xenograft breast cancer sample

To evaluate CONET performance on real data, we apply the model to 260 xenograft breast cancer cells in the SA501X3F data set [49], sequenced using the Direct Library Preparation (DLP) method. This specific scDNA-seq technology omits the pre-amplification step, thus ensuring better coverage uniformity which in turn allows for more reliable CNAs identification.

To validate the performance of CONET in a setting where the ground truth is unknown, we compare the results of CN calling procedure to those obtained with HMMCopy, CBS+MergeLevels, SCICoNE, and simple rounding of corrected counts to nearest integer (CC-rounding), and the CONET structure to the tree inferred by SCICoNE.

#### CONET structure for SA501X3F xenograft breast cancer data

**Fig. 4:**
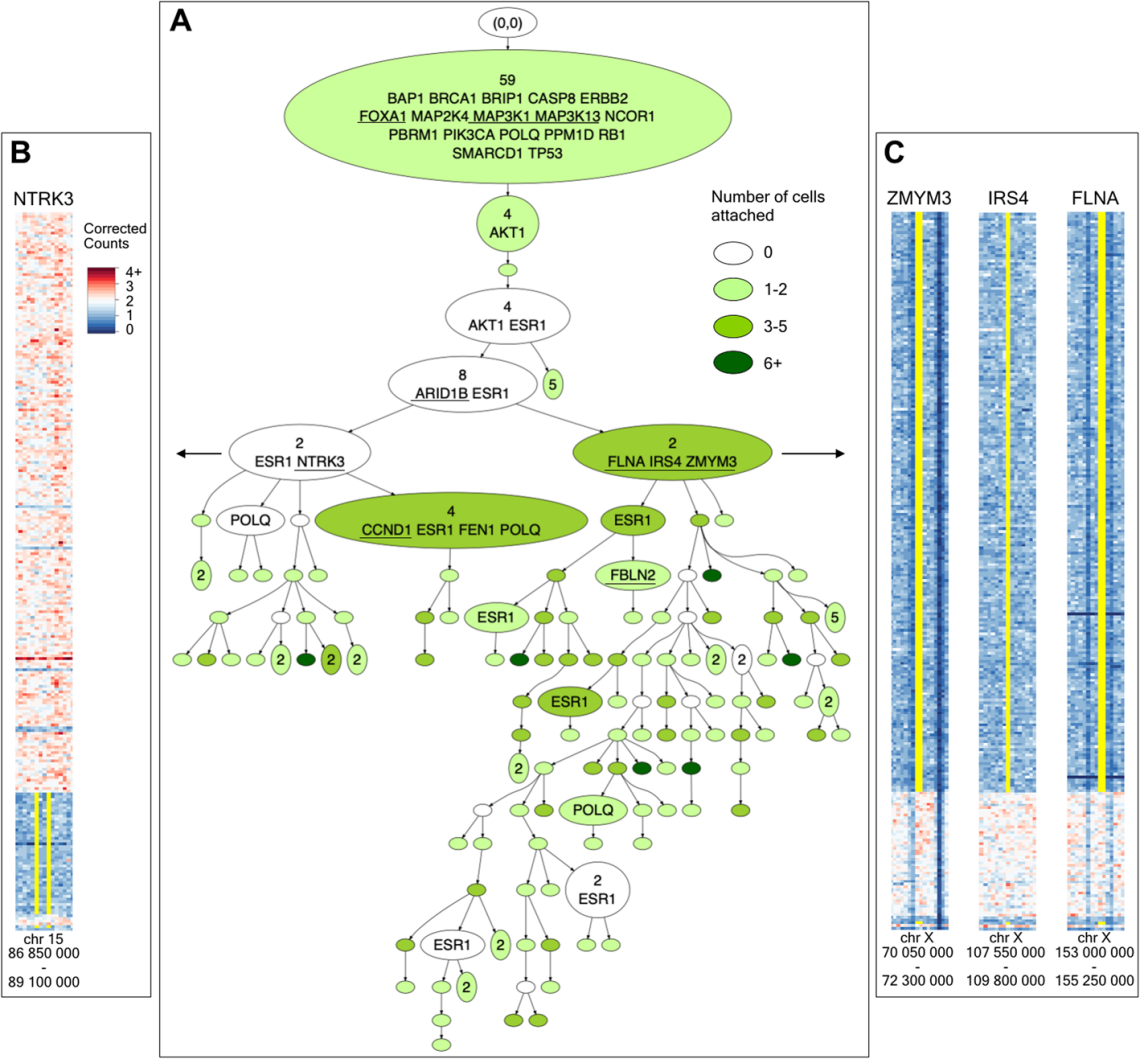
CONET for SA501X3F xenograft breast cancer data set. **A** The CONET is drawn in a compacted form, with part of vertices collapsed when it is possible without losing important information, i.e. when the collapsed vertex has no cells attached and not more than one child. Specifically, the collapsed vertex is joined with its closest descendant that does not satisfy these criteria (number of events is shown at the beginning of joint vertices). This results in decreasing the tree size from 225 to 131. The number of cells attached to each vertex is illustrated with different colors, where white vertices have no cells attached and the darker green indicates more cells attached. The names of the breast cancer genes [67] affected by the CN events are printed in alphabetical order in the corresponding vertices. Underlined genes appear only once in the CONET. **B**, **C** Enlarged parts of corrected counts matrix heatmap, with areas of the genome surroundinng the coding regions of such breast cancer genes that divide the cells into two distinctive subpopulations. The yellow lines are the borders of each of the genes’ coding regions, drawn for cells that have CN change of the gene in question, according to their attachment in the CONET

The inferred CONET (see Additional file 1: Section S1 for run settings) has a complex structure, indicating a high level of instability in the cancerous genome (Fig. 4A). In total, the events in the tree overlap with 27 genes determined as associated with breast cancer by the COSMIC Cancer Gene Census [67]). Additional file 2 presents the comprehensive list of all the CONET vertices with information about their parent, genomic coordinates of each event and breast cancer genes affected by the event.

**Fig. 5:**
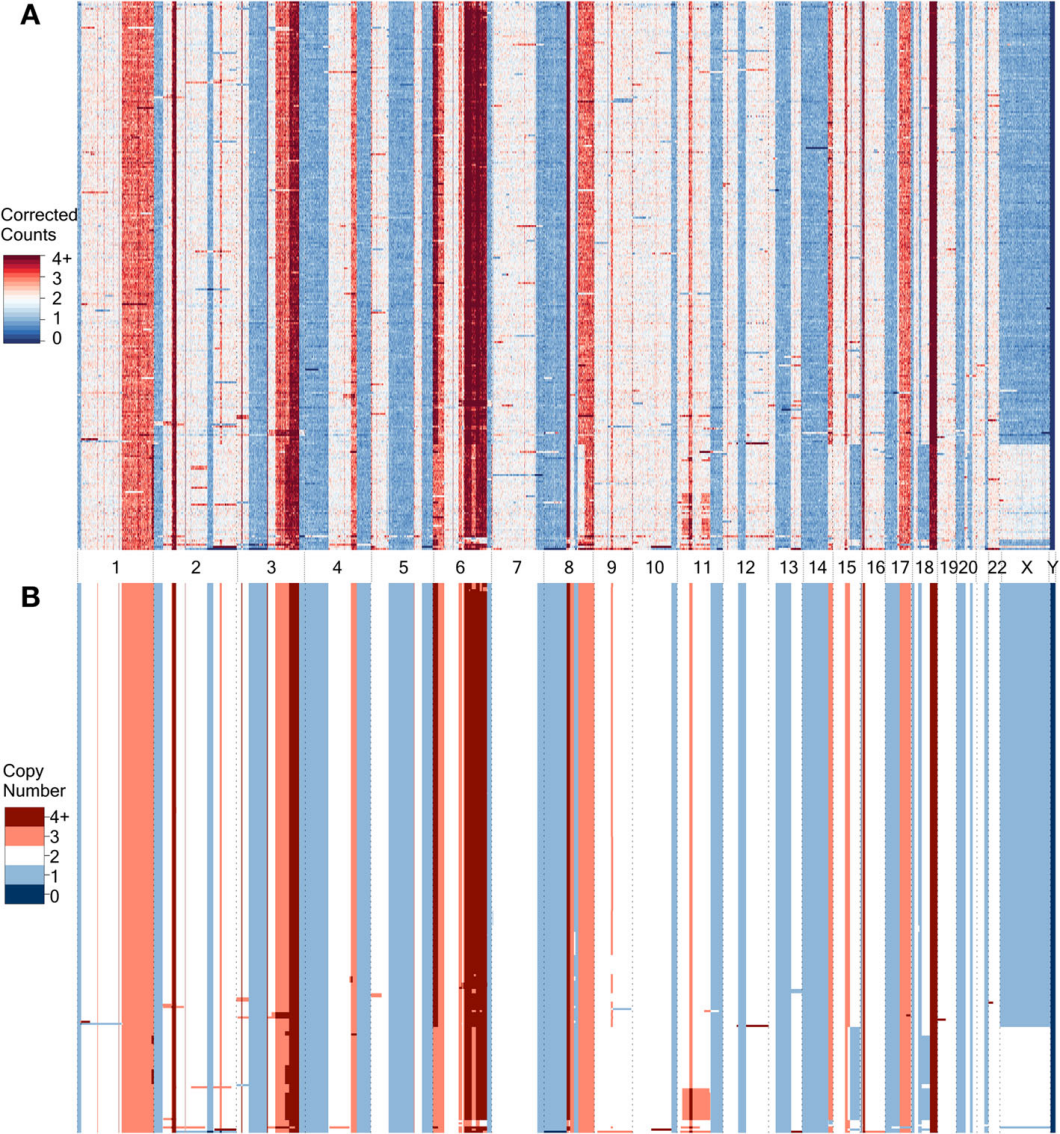
Graphical illustration of CN calling results for SA501X3F xenograft breast cancer data set. **A** The CC heatmap illustrates the biological data with corrected counts in genomic bins, **B** the CN heatmap presents the inferred integer CN for equivalent bins. Columns correspond to genomic locations, rows to single cells. The rows in both matrices are in the same order fixed using hierarchical clustering of cells according to their inferred CNs

Looking closer at the inferred CN events history, we first observe a linear evolution of the tumor, where the vast majority of cells undergo a series of common CN events, visible in the trunk of the tree. Those events overlap with many characteristic breast tumor suppressor genes such as *BRCA1*, *TP53*, *RB1*, or *CASP8* [67]. CN events that can cause a decrease in expression of those genes (all genes fall into clusters with inferred CN equal to one—see Fig. 5) could have contributed to the onset of breast cancer. The next significant event in the evolutionary history of this tumor sample is the branching of the trunk that divides the cells into two distinct subgroups, the smaller one (left of the tree in Fig. 4A) characterized by unique *NTRK3* gene CN change, and the more abundant (on the right of the tree in Fig. 4A), distinguished by unique *FLNA*, *IRS4*, and *ZMYM3* CN changes (with inferred CN equal to one). Figure 4B presents an enlarged fragment of corrected counts heatmap with the genomic region around *NTRK3* where the gene location and subpopulation of cells with lowered CN according to the inferred CONET is marked in yellow. The second subpopulation of cells, which emerged during main tree branching, is shown in Fig. 4C across fragments of corrected counts heatmaps surrounding *FLNA*, *IRS4* and *ZMYM3*. It can be speculated that the CN event affecting the *NTRK3* gene could have been part of a known translocation causing the formation of the *ETV6-NTRK3* fusion oncoprotein characteristic of human secretory breast carcinoma [68]. This event could result in an evolutionary advantage for the subpopulation of cells that are attached under the vertex describing this CN event. Among the genes characterising the other larger subpopulation, *ZMYM3* is a known tumor suppressor [67], whose deficiency impairs DNA repair by homologous recombination and can result in high genome instability [69]. Evidence of this instability is clearly visible in the CONET structure for this subpopulation where we distinguish numerous CN events that do not overlap with new breast cancer genes. Another interesting observation can be drawn from genes such as *ESR1* or *POLQ* (both falling into regions with amplified CN for most of the cells—not shown) that reappear many times in the events of the inferred CONET, also in parallel branches, suggesting that some genomic regions have very high instability and are more prone to CNAs. This also points to the fact that the evolutionary trees reconstructing CN changes do not follow the perfect phylogeny assumption.

#### CN calling for SA501X3F xenograft breast cancer data

Figure 5 graphically represents the high quality of the CN calling procedure for SA501X3F xenograft breast cancer data. The regions with clearly visible deviations from the neutral CN in the noisy corrected counts heatmap (Fig. 5A) are for the most part correctly identified as such in the inferred integer CN heatmap (Fig. 5B). At the same time, the inferred CN heatmap lacks the noise observed in the true corrected count data. Importantly, the fidelity of CN events’ reconstruction is maintained even for very narrow genomic events (sometimes only one bin wide). This is especially true for CN events that are common for a large enough number of cells.

#### Comparison to other methods

##### Comparison of CN calling results.

To demonstrate the advantage of the proposed CN calling procedure, we compare it to SCICoNE (inferred CN heatmap featured in [64]), HMMCopy (inferred CN heatmap featured in [49]), circular binary segmentation (CBS) [52], followed by MergeLevels [54] (CBS+MergeLevels; inferred CN heatmap in Additional file 1: Fig. S3), as well as CC rounding (Additional file 1: Fig. S4). The CBS+MergeLevels procedure is applied by MEDALT [62] prior to inference of the cell lineage tree. We run all methods on the same SA501X3F xenograft breast cancer data using default parameters (Additional file 1: Section S2). The agreement between the CN matrices inferred by the methods under comparison and the data is measured using root mean square error, denoted CNCC-RMSE. CNCC-RMSE is calculated as the root of quadratic mean of the differences between inferred integer CN and corrected count in each bin. It is important to realize that in contrast to CN-RMSE computed for simulated data, here the CNCC-RMSE is the distance to the noisy per-bin data and it can not be minimized to zero. Additionally, for each inferred CN matrix, we identify CNAs as maximal regions spanning sets of consecutive bins with equal CN. A CNA is then a region within a cell’s genome, with specified start and end loci, and integer CN. A CNA in another cell with the same start and end but different CN is counted separately. Next, we compute the average number of CNAs per cell, the total number of unique CNAs across the CN matrix, and the number of singleton CNAs. Singleton CNAs are defined as CNAs that were detected only in one cell (Table 1).

**Table 1:** Assessment of CN calling methods for SA501X3F xenograft breast cancer data

Method	CNCC-RMSE	Avg. CNAs per cell	Unique CNAs	Singleton CNAs
CONET	0.43	86.8	374	153
HMMCopy	0.82	43.74	1871	1257
CBS+MergeLevels	0.39	79.95	3860	2757
SCICoNE	0.61	93.55	118	4
CC-rounding	0.25	3761	200,013	100,306

The CNCC-RMSE for HMMCopy is equal to 0.82, which is almost twice as high as the one for CONET. The CBS+MergeLevels obtains the lowest CNCC-RMSE equal to 0.39. However, even though the average number of CNAs per cell is similar for CONET and CBS+MergeLevels, the latter method infers over ten times more unique CNAs (3860) and singleton CNAs (2757) than CONET (374, 153). Compared with CNAs occurring in more than one cell, singleton CNAs are more likely to be false positive calls. These results suggest that CBS+MergeLevels overfits the data, while CONET is more focused on capturing events that play an important role in tumor evolution. These conclusions are in agreement with those drawn using simulated data (see Performance of CONET in comparison to other methods on simulated data). SCICoNE obtains a relatively high CNCC-RMSE (0.61) but also the smallest number of inferred events (118)—over three times less than CONET. At the same time, SCICoNE’s average number of CNAs per cell is the highest of all methods. This indicates that SCICoNE focuses on events that affect the whole population but neglects local CNAs. By far the worst results are obtained by CC-rounding: in comparison to CONET, it detects two orders of magnitude larger average number of CNAs per cell (3761), as well as an extremely high number of unique CNAs (200,013) and singleton CNAs (100,306). With the lowest CNCC-RMSE of only 0.25, CC-rounding again clearly overfits to noise in the data.

Overall, using CONET improves the accuracy of calling integer CNs in genomic bins. Compared to other methods, CONET maintains a good fit to the count data, while avoiding false positive calls.

##### Comparison of event tree inference.

We first compare the CONET obtained for the xenograft breast cancer SA501X3F data (Fig. 4) to the tree obtained by SCICoNE in [64] and asses the sets of breast cancer genes[67] affected by the events in these trees. The trunks of both trees include a similar number of genes but they are not in full agreement, with the main difference constituting *BRCA1* and *CASP8*, both present in the CONET trunk, but the first one not present in the SCICoNE tree at all, and the second one affecting only 4 cells. Another important difference is due to the *NTRK3* gene which is present in the SCICoNE trunk, but in the case of CONET is altered in only around 20% of cells and characterizes the smaller of the two main subclones. This division into two subclones is not clearly visible in the SCICoNE tree, although *IRS4*, one of the genes characterising the second subclone, appears on a similar level in both trees. Both trees also share recurrent events affecting the *ESR1* gene, while the *POLQ* gene, reappearing in CONET, does not show up in the SCICoNE tree at all. Taking into consideration that both models are novel approaches to a very difficult problem, it is very promising that they partly share biological conclusions drawn from noisy experimental data set. Further research is needed to establish whether the differences in the results come from the models themselves or the weak signal present in the data.

We also compare CONET to the event trees that are the output of MEDALT for the xenograft breast cancer SA501X3F data. In contrast to CONET, MEDALT does not directly model the per-bin nor per-breakpoint data, but requires that the integer CNs are already called for each bin and provided as input. We thus apply MEDALT to three different CN matrices, as inferred by CBS+MergeLevels, HMMCopy and CONET. We do not expect a large overlap between the CONET and MEDALT trees, as MEDALT intends to select only driver events. Still, regardless of the way the input CN calling was performed for MEDALT, the breast cancer genes affected by significant events are also found affected by CONET. It turns out, however, that the MEDALT output trees and the sets of affected genes largely differ depending on the input CN matrix (Additional file 1: Section S3 and Fig. S5). This problem does not occur for CONET, as it infers the event tree structure and performs CN calling simultaneously. These results indicate favorable performance of CONET in comparison to other related methods on real scDNA-seq data.

#### Additional validation of CONET using passage SA501X4F xenograft breast cancer sample

To additionally validate CONET inference, we run CONET for a total of 513 single cells, from two xenograft breast cancer samples: SA501X3F (260 cells) and it’s subsequent passage, SA501X4F (253 cells) [49].

Comparison of the CONET structures for the joint SA501X3F, SA501X4F sample and SA501X3F alone (Additional file 1: Fig. S6) reveals that for the joint sample the general tree structure is maintained, with a long trunk and further branching of the tree, which divides cell population into two main subclones. The fact that events from the trunk and branching vertices of both trees overlap with mostly the same breast cancer genes confirms the consistency of CONET inference procedure. Interestingly, the cells from the passage sample SA501X4F attach only to the larger one of the two main subclones. The smaller subclone contains only the cells from the earlier SA501X3F sample in both trees. This may be due to the fact that the cells from the passage were sampled from a similar localization, populated with cells derived from only one of the subclones. A similar observation that the passage maintained only one of the subclones found in the SA501X3F sample, was made by [49].

### Application to scDNA-seq data of TN2 invasive ductal carcinoma sample

To demonstrate CONET applicability to an additional experimental data set, obtained using a different scDNA-seq technique, we apply the model to 1024 invasive ductal carcinoma cells in the TN2 data set [51]. This sample was sequenced using the ACT method, which has less experimental steps, shorter processing time and higher throughput compared to other scDNA-seq techniques relying on whole genome pre-amplification steps [51]. Moreover, since the sample has a reported ploidy equal to 3.03 [51], we demonstrate the applicability of CONET model to data with basal ploidy set to three.

#### CONET structure for TN2 invasive ductal carcinoma sample

The CONET for TN2 invasive ductal carcinoma data set has a complex structure, indicating a high level of instability in the cancerous genome (Additional file 1: Fig. S7). In total, the events in the tree overlap with 41 genes determined as associated with breast cancer by the COSMIC Cancer Gene Census [67]). Additional file 3 presents the comprehensive list of all the CONET vertices with information about their parent, genomic coordinates of each event and breast cancer genes affected by the event.

Looking closer at the inferred CN events history, we first observe a linear evolution of the tumor, where the vast majority of cells undergo a series of common CN events, visible in the trunk of the tree. Those events overlap with many characteristic breast tumor suppressor genes such as *BRCA1*, *BRCA2*, *CHEK2* [67] with inferred CN lower than basal ploidy. The next significant event in the evolutionary history of this tumor sample is the branching of the trunk that divides the cells into two distinct subgroups. The left one is characterized by *ARID1A* gene CN change (deletion according to the inferred CN matrix illustrated in Additional file 1: Fig. S8B), with further branching: the most left subranch characterized by *ARID1A* CN change and middle branch - *RB1* CN change (both amplified according to the inferred CN matrix). The other main subclone on the right is distinguished by unique *SALL4* CN change (amplified according to the inferred CN matrix). Genes such as *MAP3K13*, *PIK3CA*, *POLQ* appear repeatedly in the in CONET, which is due to the fact that they are located in the genomic regions which are highly amplified according to the inferred CN matrix.

#### CN calling for TN2 invasive ductal carcinoma sample

Additional file 1: Fig. S8 graphically represents the high quality of the CN calling procedure for TN2 invasive ductal carcinoma data. The regions with clearly visible deviations from the basal ploidy 3 in the noisy corrected counts heatmap (Additional file 1: Fig. S8A) are for the most part correctly identified as such in the inferred integer CN heatmap (Additional file 1: Fig. S8B). At the same time, the inferred CN heatmap lacks the noise observed in the true corrected count data. Importantly, the fidelity of CN events’ reconstruction is maintained even for very narrow genomic events (sometimes only one bin wide). This is especially true for CN events that are common for a large enough number of cells.

### Performance of CONET in three additional challenging tasks

Additionally, we evaluate CONET in three challenging tasks.

First, we evaluate whether CONET correctly recovers the ordering of cells and their clustering represented by cell attachment. The ancestor–descendant relations among cells are assessed using measures called *ancestry recall* and *branching recall* (Additional file 1: Fig. S9; see “Model evaluation metrics for simulated data” section for the definition of the measures). Such relations are induced by the ordering of nodes in the evolutionary tree, as the cells are assigned to nodes. For the evaluation we simulate data in the challenging setting with high noise (with tree size in {20,40} and number of cells in {200,1000}). We test the performance of CONET in three different versions: CONET with given true candidate breakpoint loci (CONET (known)), and with candidate breakpoint loci found using either HMMCopy, or CBS+MergeLevels. With median ancestry recall scores greater than 0.65 for CONET (HMMCopy), greater than 0.7 for CONET (CBS+MergeLevels) and greater than 0.9 for CONET (known) across all scenarios, together with median branching recall scores greater than 0.7 for CONET (HMMCopy) and greater than 0.75 for CONET (CBS+MergeLevels) and CONET (known) across all scenarios, CONET proves to correctly recover ancestry relations of cells. We exploit the same simulation setups to evaluate the clustering of cells by calculating *rand index* between true and inferred cell attachment (Additional file 1: Fig. S9; see “Model evaluation metrics for simulated data” section for the definition of the measures). With median rand index greater than 0.93 across all scenarios, CONET demonstrates excellent performance of clustering the cells by attaching them to correct tree vertices.

Second, we investigate the performance of CONET in detecting short versus long events (Additional file 1: Section S4 and Fig. S10). This analysis reveals that event detection performance is similar for long and short events.

Third, we investigate the performance of CONET (known) for an increasing number of cells. Here, we do not evaluate CONET with breakpoint loci identified using HMMCopy nor CBS+MergeLevels, since the number of candidate loci would differ for varying number of cells and would be a confounding factor influencing the performance. We simulate the data for models with varying number of cells (values from {50,500,1000,2000,10,000}), tree size 40 and high noise (20 models for each scenario). Values for the tree structure prior are scaled linearly in order to achieve comparable sizes of inferred trees. Computation time is approximately linear, proving very good scalability of CONET (Additional file 4: Table S1). All tree quality measures (except for Node precision) increase with the growing number of cells, indicating that CONET inference quality benefits from larger data.

### CONET convergence analysis

Next, we inspect CONET convergence on both real and simulated data. The likelihood trace plots for multiple CONET runs for the SA501X3F xenograft breast cancer sample show high agreement between different runs and indicate likelihood convergence (Additional file 1: Fig. S11).

Similarly, the likelihood trace plots for CONET (known), CONET (HMMCopy), and CONET (CBS+MergeLevels) on the synthetic data with high noise (described in “Simulations for comparative evaluation of CONET on both per-bin and per-breakpoint data” section) indicate convergence to some local maximum with comparable likelihood values (Additional file 1: Fig. S12). Trace plots for 1000 cells are more spread out. This is to be expected, as the more cells, the more possibilities to add a subclone, which improves the likelihood.

Despite high noise in the analyzed simulated data, inferred model parameters *θ* (defined in CONET - Copy Number Event Tree model) exhibit excellent reproducibility, with very small differences among parameters inferred from different runs, across varying tree size and number of cells (Additional file 5: Table S2). Moreover, the results of CONET (known) and CONET (CBS+MergeLevels) are highly consistent. CONET (HMMCopy) shows less agreement with these two, with most noticeable differences for models with 200 cells.

### Assessment of the impact of the regularization parameters and of data separability on CONET performance

#### Assessment of impact of the tree structure and attachment priors, as well as of data separability on CONET performance on simulated data

In addition, we show that CONET achieves excellent performance for a range of different priors that can be used for the model, as well as different levels of separability of the per-breakpoint data (separability of the absolute count differences’ distribution for the breakpoint loci from the absolute count differences’ distribution for loci where there are no breakpoints; Additional file 1: Section S5). In this analysis CONET performance is evaluated in the tasks of tree reconstruction and breakpoint detection when only limited per-breakpoint data are available, without access to the per-bin data. Other methods are not evaluated in this setting, as they are not applicable to per-breakpoint data only.

This analysis indicates that the choice of the regularization constant for the tree structure prior calibrates the size of the final tree, and the trade-off between edge sensitivity and precision. Indeed, for stronger tree structure prior and smaller trees, only nodes corresponding to events with strong support in the data are be found. Consequently, CN calling identifies events shared across many cells, which are likely to be true positives, giving overall high precision. At the same time, however, nodes corresponding to rare events might be missed, causing false negatives in CN calling and low sensitivity. For relaxed tree structure prior larger trees are constructed, with many nodes, and sensitivity is high. In this setting, however, the leaves of the tree may correspond to events that are false positives, lowering precision. Addition of the attachment prior improves the performance for most metrics and simulation settings. In this challenging setting, where only the per-breakpoint data is available, good data separability is a determining factor of the quality of the results.

#### Assessment of impact of the count discrepancy penalty on CONET performance based on the xenograft breast cancer SA501X3F data

We demonstrate the advantage of using both per-breakpoint and per-bin data by evaluating the quality of the CONET structure and CN calling with and without the discrepancy penalty (Additional file 1: Section S6, Additional file 6: Table S3 and Additional file 7: Table S4). This analysis clearly indicates that an additional regularization in the form of the count discrepancy penalty is necessary when dealing with noisy, low-depth scDNA-seq data from real experiments.

Intuitively, the count discrepancy penalty has two effects, controlled by two separate parameters. First, it prioritizes models that yield CN calls that are closer to the noisy per-bin count matrix. Second, it penalizes trees that create regions changed by CN events and having inferred CN equal to two (or the basal ploidy). Similarly to the tree structure prior, an adjustment of this penalty influences the size of the tree. With too large penalty, the model may over-fit to noisy per-bin data, and grow a large tree that predicts many events, resulting in high sensitivity but low precision. With lower penalty, the sensitivity decreases but the precision increases. It is recommended to use dedicated model quality measures (see Additional file 1: Section S6.1) to test possible regularization parameter choices (see Additional file 1: Section S7).

## Discussion

CONET differs from other recent evolutionary models of breakpoints or CN events: the sitka model [61], MEDALT [62] and SCICoNE [64]. It is the only tree with nodes labeled by pairs of breakpoints. For the trees inferred by [61], the nodes do not correspond to CN events. Instead, each node of such tree corresponds to an acquisition of only a single breakpoint. The MEDALT model is a lineage tree spanning the input cells. As a post-processing step, it can construct trees on events that it identifies as significant. In contrast, SCICoNE directly models an event tree, where each node is labeled with a vector of starts and ends of CN events, together with the CN change that occurred for each of the events. CONET deliberately avoids modeling the exact CN changes acquired at each event, thereby vastly reducing the space of possible models.

The reduced space and efficient MCMC implementation facilitate advantageous computation times for CONET inference procedure. The main factors influencing the run time are the number of cells and the size of inferred CONETs, which can be regulated by the user with the number of potential breakpoints, tree structure priors and the choice of corrected counts penalty. The control over model parameters and regularization will give a particular advantage when dealing with the much larger and hopefully less noisy data sets, which can be anticipated in the future, given the constant advances in whole genome scDNA-seq techniques.

CONET bears also several differences to existing approaches to copy number calling in single-cell data, such as HMMCopy [55], the CBS [52] algorithm, Gingko [57], Aneufinder [56] or Scope [58]. First, CONET calls only such copy number events that are explained by the evolutionary tree. Intuitively, since the copy number changes are in fact acquired in the course of evolution, this may reduce false positive CN calls. Indeed, this enables CONET to combine information from bins that undergo the same evolutionary history and avoid over-fitting to the noise in the data. On top of that, model regularization parameters allow to steer the tree size and consequently control the level of detail of the output copy number calling, from small trees explaining events that are the earliest in tumor evolution and thus result in copy number changes that are the most common across cells, to large trees that incorporate rare events that occur only in small fractions of cells. Second, while some CN calling methods utilize both the per-bin and per-breakpoint signal in the data, they define the per-breakpoint signal differently and use it to infer segments of the genome that share the same CN, either via a hidden Markov model (HMMCopy) or segmentation algorithm (CBS or Gingko). Instead, CONET directly models the probability distribution of per-breakpoint data (as defined in Model overview) and uses it to calculate the likelihood of a CN tree, which is additionally regularized with the per-bin data. Third, CONET does not perform raw data mapping and pre-processing and assumes the input data is normalized and corrected for biases. This is similar to the other evolutionary models of breakpoints or CN events, the sitka model [61], MEDALT [62] and SCICoNE [64]. Pre-processed data is also commonly required at input for multiple approaches to SNV evolution modeling from scDNA-seq data [31–35]. The advantage of separating pre-processing from evolutionary inference and copy number calling is applicability to a wide range of sc-DNAseq protocols, which ideally should be pre-processed using a dedicated pipeline. Indeed, here we show CONET can successfully be applied both to pre-processed DLP and ACT data.

As the strength of CONET lies in an integrative approach - simultaneous tree inference and copy number calling, as well as combining per-breakpoint and per-bin signal - it could potentially further benefit from incorporating additional signals. For example, such as implemented by ReMixT [70], a bulk sequencing-based approach to clone-specific genomic structure estimation in cancer, CONET could be extended to model genomic structural variants and reconstruct the true genomic topology. Another possible extension of CONET would be, similarly to CHISEL [59], to combine signal across cells to infer allele-specific CNs.

Deciphering the gene CN evolution of tumors is of huge importance for the understanding of the process of carcinogenesis. Using CONET, we analyzed the evolutionary history of CN events and CN changes in single cells for a xenograft breast cancer sample. Evaluation of the trees obtained using different regularization schemes indicates that in the case of modeling real data, penalization for the disagreement between the estimated and actual per-bin counts is crucial for constraining the model and obtaining high-quality trees. Comparison of the corrected count per-bin data with inferred CN profiles illustrates the excellent performance of breakpoint identification and the CN calling procedure for the breast cancer sample. We observe the smoothing of the noisy biological signal for less evident events. Since the biological truth is unknown, it is difficult to assess the quality of the inferred CONET structure. Still, the inferred CONET identifies CN events that occur early in tumor evolution, amplifying or deleting cancer driver genes such as for example *BRCA1*, *TP53*, *RB1*, or *CASP8*, all of which play pivotal roles in breast cancer progression. These trunk alterations could potentially suggest the choice of efficient anticancer treatment. On top of that, our approach distinguishes subclones characterized by unique *ZMYM3*, *IRS4*, *FLNA*, and *NTRK3* deletions caused by events from specific tree sub-branches. The alterations of these genes can in turn confer resistance to therapy, and as such can highlight the need for additional therapeutic intervention or combinatorial treatment. Similarly, application of CONET to the TN2 invasive ductal carcinoma data brought novel insights on its evolution and copy number changes to breast cancer genes.

Applicability of CONET extends beyond the tasks presented in this work. For example, the accurate CN calls from CONET can provide valuable input to methods that rely on CNA information for improved elucidation of tumor subclones and their relationships from variant allele frequencies in bulk sequencing data [71, 72]. Compared to the CN calls alone, CONET output can define even more specific constraints for reliable modeling of evolutionary SNV trees inferred from high coverage scDNA-seq [73]. Ultimately, once scDNA-seq data from larger cohorts is available, the application of CONET will help to uncover general patterns of the order of occurrence of CNAs in tumors together with their importance. In summary, CONET is a powerful tool that takes advantage of scDNA-seq to better understand the CN evolution in cancer and may guide the choice of therapy when applied to patient data in the clinic.

## Conclusions

Recent developments of high-throughput scDNA-seq technologies have enabled tracing CN changes in single-cell genomes. To our knowledge, CONET is the first Bayesian probabilistic approach for joint CN evolution inference and CN calling that fully exploits the scDNA-seq readouts. CONET is robust to errors in scDNA-seq data, as it models the per-breakpoint and per-bin readouts in a probabilistic manner. By combining the process of tree inference and CN calling, it gains power in both tasks. We propose an efficient MCMC procedure for the search across the space of possible trees and model parameters.

CONET performs favorably in comparison to other methods in terms of evolutionary tree reconstruction and CN calling both on simulated and real data. Comprehensive analysis on simulated data indicates that CONET excels in the correct breakpoint identification for single cells, even in the case of more demanding scenarios with bigger trees and more noisy data. The model enables accurate inference of the model structure, as measured by the event and edge precision, as well as event and edge sensitivity.

## Methods

### Real data pre-processing

#### SA501X3F data set

To illustrate the performance of our model on true biological data we apply it to scDNA-seq data from 260 xenograft breast cancer cells in SA501X3F data set [49] sequenced using Direct Library Preparation (DLP) method. According to [49] the sequencing reads were binned into 150 kilo base bins, corrected for GC content and mappability and normalized such that 2 signifies a neutral CN. The resulting positive real numbers for each bin are further called corrected counts in bins and constitute the per-bin input to our model. We calculate the corrected count absolute differences by subtracting corrected counts in the adjacent bins and taking the absolute value. The resulting corrected count absolute differences matrix with genomic loci in columns and cell ids in rows represents the per-breakpoint input to the model.

To run our model, we also need to establish candidate breakpoint loci set. The set of candidate loci determines the set of possible CN events and as a consequence significantly influences the computational complexity of the model inference procedure. The candidate breakpoints are set by the user.

Here, we run HMMCopy [55] implemented as Bioconductor package using corrected counts as input with standard parameters, obtaining integer CN state for each bin. We further assume that each locus with inferred CN state change in adjacent bins is a possible breakpoint. To this set of candidate breakpoints we also add the beginning and end of each chromosome, together with loci which show high corrected count absolute difference evidence (more than 80% of cells with the corrected count absolute difference higher than 3). To complete our potential breakpoint loci set, for each of the above mentioned candidates (except chromosome ends) we add one locus to the right to be always able to infer short one bin events. The final set of candidate loci for the SA501X3F data set contains 2044 possible breakpoints. After this step, we prepare the final corrected count absolute differences input matrix restricted to chosen candidate breakpoint loci columns.

#### SA501X4F data set

To additionally validate the performance of our model on true biological data, we use scDNA-seq data from 253 xenograft breast cancer cells in SA501X4F data set [49]. This sample is a passage from SA501X3F sample and we apply the same pre-processing protocol as described in SA501X3F data set. Then we join the corrected counts and count absolute differences matrices and perform the CONET inference for total of 513 cells (in columns). We also join the potential breakpoints set adding 88 new potential breakpoints.

#### TN2 data set

To demonstrate CONET applicability to an additional experimental data set, sequenced using a different scDNA-seq technique (ACT method [51]), we apply the model to 1024 invasive ductal carcinoma cells in the TN2 data set [51]. For this data set we assume basal ploidy *n*_*CN*_=3, based on the average ploidy 3.03 and whole-genome duplication event for this sample inferred by [51]. The sequencing reads were binned into bins with varying width (average 222,349 bases) and corrected for GC content as described in [51] (the reads in this pre-processed form were obtained from the authors of [51]). For the normalization we divide bin counts by sample mean bin size and then multiply by the average ploidy equal to 3.03. Subsequently we divide each bin count by its bin width and multiply by average bin width. The resulting positive real numbers for each bin are further called corrected counts in bins (corresponding to copy number ratios in [51]) and constitute the per-bin input to our model. We calculate the corrected count absolute differences by subtracting corrected counts in the adjacent bins and taking the absolute value. The resulting corrected count absolute differences matrix with genomic loci in columns and cell ids in rows represents the per-breakpoint input to the model.

To establish candidate breakpoint loci set, we start with log mean segment ratios for tumor TN2 inffered by [51] using Bioconductor package copynumber (v1.26) [65], followed by MergeLevels [54]. We transform obtained log segment ratios into ratio values, multiply by average ploidy and round to closest integer. We further assume that each locus with inferred CN state change in adjacent bins is a possible breakpoint. To this set of candidate breakpoints we also add the beginning and end of each chromosome, together with loci which show high corrected count absolute difference evidence (more than 80% of cells with the corrected count absolute difference higher than 3). To complete our potential breakpoint loci set, for each of the above mentioned candidates (except chromosome ends) we add one locus to the right to be always able to infer short one bin events. The final set of candidate loci for the TN2 data set contains 329 possible breakpoints. After this step, we prepare the final corrected count absolute differences input matrix restricted to chosen candidate breakpoint loci columns.

### CONET - Copy Number Event Tree model

Below, we explain CONET, a generative probabilistic model for inferring tumor evolution on single-cell CN events. Let *C*={*c*_*j*,*i*_}_*j*∈{1,…,*m*},*i*∈{1,…,*n*}_ denote a data set of corrected read counts from *n* genomic bins for a total of *m* cells. We assume the read count data are pre-processed, in particular, normalized to basal ploidy *n*_*CN*_, corrected for GC content and other potential biases (the methods for correcting the biases in the data need to be adjusted to a given scDNA-seq technique). For each chromosome we add an artificial bin representing the end of a chromosome with corrected count equal to basal ploidy *n*_*CN*_. This is necessary for being able to include CN events that start or end at the ends of a chromosome, i.e., physically have only one breakpoint. The data modeled by CONET is defined as the absolute differences of counts at consecutive bins:

1 $$ d_{j,i}= \left\{\begin{array}{ll} |c_{j,i}-c_{j, i - 1}|& \text{if}\ {i \ge 2} \\ |c_{j,i} - {n_{CN}}| & \text{otherwise.} \end{array}\right.  $$

Each difference is indexed with *j*, denoting the cell it is calculated for, and with *i*, denoting the genomic locus (or interchangeably - the genomic bin starting in this locus). We assume a set of loci *L*⊂{1,...,*n*} is given. We define a CN event as an ordered pair of loci (*i*,*l*) from *L*, such that *i*<*l*, i.e., locus *i* occurs before *l* in the genome, and loci represented by *i*,*l* lie on the same chromosome. We assume that in bins [*i*,*i*+1,...,*l*) the CN changed during the evolutionary history of cells that underwent this event. Since the loci *i* and *l* mark the endpoints of the deleted or amplified regions, we refer to them as breakpoints. Loci from *L* will be referred to as candidate breakpoints.

We assume that each CN event can occur only once in the tumor evolution. Still, one breakpoint can be part of many CN events and the events can overlap. In this sense, our tree does not satisfy the infinite sites assumption.

Let *D* denote the data matrix of absolute count differences for such bins that start at candidate breakpoints, i.e., such that for *d*_*j*,*i*_ it holds *i*∈*L* and *j*∈{1,…,*m*}.

CONET is a tuple (*T*,*σ*,*θ*), where *T* is the evolutionary tree structure, *σ* is referred as cell attachment, and *θ* denotes the set of model parameters. Let *T* denote a directed rooted tree with a set of vertices *V*_*T*_ corresponding to CN events and edges to the partial order of these events. Additionally, *V*_*l*_ denotes the set of all the tree leaves and *V*_0_ denotes the set of all possible events, which are not present on the tree (i.e., that do not belong to *V*_*T*_). We refer to *V*_0_ as the set of inactive events, an(*v*) denotes the ordered set of vertices (including vertex *v*) that occur on the path from vertex *v* to tree’s root and depth(*v*) denotes the depth of vertex *v* in the tree. The root of the tree is given as an artificial event denoted by (0,0), corresponding to no somatic CN events.

The attachment of cells to the tree is given by a function *σ*:{1,…,*m*}→*V*_*T*_. For a given cell *j*, the path from *σ*(*j*) to the root of the tree defines the set of events that the cell *j* underwent and their order (an(*σ*(*j*))). It also specifies the set of breakpoints that this cell has. Indeed, for a given tree *T* and cell attachment *σ*(*j*), the set of breakpoints of cell *j*, denoted by *I*_*bp*_(*j*|*T*,*σ*) is given by the breakpoints that occur in at least one event on the path an(*σ*(*j*)). We also define *I*_0_(*j*|*T*,*σ*) to be the set of remaining candidate breakpoints in *L*, i.e., *I*_0_(*j*|*T*,*σ*):=*L*∖*I*_*bp*_(*j*|*T*,*σ*).

The set of parameters *θ* parametrizes the distributions of the absolute count differences, depending on whether they are calculated at breakpoints or not. The absolute count differences at breakpoints are expected to be large, while differences at loci that are not breakpoints should oscillate around zero. Accordingly, for a given tree *T* and attachment *σ*, we have *d*_*j*,*i*_∼*f*(*d*_*j*,*i*_|*T*,*σ*_*j*_,*θ*), where

$$ f(d_{j,i} | T, \sigma_{j}, \theta) = \left\{\begin{array}{ll} f_{bp}(d_{j,i}|\theta_{bp})& \text{if}\ i\in I_{bp}(j | T, \sigma) \\ f_{0}(d_{j,i} |\sigma_{0}) & \text{if}\ i\in I_{0}(j | T, \sigma), \end{array}\right. $$

and *f*_*bp*_ is a mixture of *K* normal distributions truncated to $\mathbb {R}_{+}$ and *f*_0_(·|*σ*_0_) is a mean zero normal distribution, also truncated to $\mathbb {R}_{+}$. Thus, $\theta _{bp} = \{ \mu _{1}, \ldots, \mu _{K}, \sigma _{1}^{2}, \ldots, \sigma _{K}^{2}, w_{1}, \ldots w_{K}\}$, where *μ*_1_,…,*μ*_*K*_, are the means, $\sigma _{1}^{2}, \ldots, \sigma _{K}^{2}$ are the variances and *w*_1_,…,*w*_*K*_ are the weights of the mixture components. The full set of CONET parameters is given by *θ*=*θ*_*bp*_∪{*σ*_0_}. The types of the probability distributions for modeling count differences were chosen after analyzing the real data distributions (Additional file 1: Fig. S1). The number of mixture components *K* is user defined. During the optimization procedure *K* is decreased when the corresponding weights decrease beyond a user defined threshold.

Thus, the likelihood of the data *D* (the count difference matrix), given unknown model constituents (tree structure, attachment, parameters) is defined to be:

2 $$\begin{array}{*{20}l} {P(D | T,\theta, \sigma)} & = \prod\limits_{j=1}^{m} \prod\limits_{i \in L} f(d_{j,i} | T, \sigma_{j}, \theta)  \\ & = \prod\limits_{j=1}^{m} \left[ \prod\limits_{i \in I_{0}(j|T, \sigma)} f_{0}(d_{j,i} | \sigma_{0})\prod\limits_{i \in I_{{bp}}(j|T, \sigma)} f_{{bp}}(d_{j,i} | \theta_{{bp}}))\right] \end{array} $$

Intuitively, the likelihood is expected to be high for such tree structures and attachments that indicate breakpoints for count differences with relatively high values and vice versa: no breakpoints for small count differences.

We assume that cells’ attachment probabilities are conditionally independent given the tree structure and do not depend on *θ*:

3 $$ P(\sigma | T, \theta) = \prod\limits_{j=1}^{m} P(\sigma_{j} | T).  $$

The exact form of *P*(*σ*_*j*_|*T*) is specified below (“Attachment prior” section). To decrease the complexity of the state space of our algorithm, we marginalize out the cell attachment vector *σ*. We furthermore use assumption (3) to transform the marginalized version of formula (2) to achieve *O*(|*L*|·*m*·|*V*_*T*_|) complexity of a single likelihood computation:

4 $$\begin{array}{*{20}l} {{P(D | T,\theta) }} & = \sum\limits_{\sigma} \left[P(D | T, \theta, \sigma) \prod\limits_{j=1}^{m} P(\sigma_{j} | T) \right] \\ & = \sum\limits_{\sigma}\prod\limits_{j=1}^{m} \left[P(\sigma_{j} | T) \prod\limits_{i \in I_{0}(j|T, \theta)} f_{0}(d_{j,i} | \sigma_{0}) \prod\limits_{i \in I_{{bp}}(j|T, \theta)} f_{{bp}}(d_{j,i} | \theta_{{bp}}) \right] \\ & = \prod\limits_{j=1}^{m} \sum\limits_{\sigma_{j}}\left[ P(\sigma_{j} | T) \prod\limits_{i \in I_{0}(j|T, \theta)} f_{0}(d_{j,i} | \sigma_{0}) \prod\limits_{i \in I_{{bp}}(j|T, \theta)} f_{{bp}}(d_{j,i} | \theta_{{bp}}) \right]. \end{array} $$

Cell attachment information is obtained from (*T*,*θ*) by choosing such *σ*, which maximizes *P*(*D*|*T*,*θ*,*σ*). Such *σ* is denoted by *σ*^∗^ and referred to as *maximal attachment*.

The described generative model deliberately ignores the actual CN change associated with each event and the actual count data observed at each bin. In this way, the state space for our model is significantly reduced. Still, the CN state for each event and each bin in each cell can easily be estimated. We utilize this fact to 1) penalize the model for inconsistency between the estimated CN state and counts in each bin and cell during training and basal plidy *n*_*CN*_) estimate the state in the bins for the final model (see below).

### Priors

Here, we define the priors: *P*(*T*) for tree structure, *P*(*θ*) for the parameters and *P*(*σ*_*i*_|*T*) for attachment given the tree structure. In all formulas below, *k*_*x*_ are model hyperparameters that are set by the user.

#### Tree structure priors

We define the tree structure prior as a product of three different prior distributions, i.e.,

5 $$\begin{array}{@{}rcl@{}} P(T) = P_{{ds}}(m, |V_{T}|, k_{1}) \cdot P_{{el}}(E, k_{0}) \cdot P_{T}(T). \end{array} $$

The *P*_*ds*_ is a prior for the tree size, following the Occam’s razor principle, that the simplest explanation is the most probable. The prior *P*_*ds*_ controls size of the inferred tree and prevents overfitting of the tree structure and is defined as follows

6 $$\begin{array}{@{}rcl@{}} P_{{ds}}(m, |V_{T}|, k_{1})=e^{-k_{1} \cdot |V_{T}| \cdot m}. \end{array} $$

This prior explicitly depends on the number of cells *m*. This constant is used to avoid over-fitting of the model to the data and constrains excessive growth of the tree size with increasing sample size.

The prior *P*_*el*_ accounts for the observation that shorter CN aberrations occur more often than the long ones [66], and favors trees with smaller total length of CN events *E*_*T*_, i.e.:

7 $$\begin{array}{@{}rcl@{}} P_{{el}}(E_{T}, k_{0})=e^{-k_{0} \cdot |E_{T}|}, \end{array} $$

where $|E_{T}| = \sum _{v \in V_{T}} |v|$ and |*v*| denotes the length of event *v* (the position of the end locus minus the position of the start locus).

The last prior is technical and serves as a stabilizer for proposal ratios of those MCMC sampling moves that change the tree size (Add leaf, Remove leaf).

8 $$ P_{T}(T)=e^{-C_{0}|V_{T}|} \quad,\text{ where}\quad C_{0}=\log\left(\frac{|V_{0}||V_{T}|}{|V_{l}|} \right).  $$

#### Attachment prior

The attachment prior *P*(*σ*_*j*_|*T*) reflects our belief about probable attachment of cell *j*. We can choose between an uniform attachment prior or assume that cell attachment depends on the tree structure and the resulting CN events. In the latter case the prior is proportional to

9 $$\begin{array}{@{}rcl@{}} {P}(\sigma_{j} = v| T) \propto \exp{\left(-\sum\limits_{u \in \text{an}(v)}\frac{|u|}{\text{depth}(v)} \right). } \end{array} $$

During cell attachment marginalization the second alternative attributes more weight to attachments that correspond to a shorter average length of events in cells.

#### Parameters’ priors

Recall that the set of parameters consists of $\theta =\left \{ \sigma ^{2}_{0}, \mu _{1}, \ldots, \mu _{K}, \sigma ^{2}_{1}, \ldots, \sigma ^{2}_{K}, w_{1}, \ldots, w_{K}\right \}$. To facilitate MCMC sampling of *θ*, we instead work with the parameters’ set

$$\theta := \left\{ \log\left(\sigma^{2}_{0}\right), \mu_{1}, \ldots, \mu_{K}, \log\left(\sigma^{2}_{1}\right), \ldots, \log\left(\sigma^{2}_{K}\right), \log(w_{1}), \ldots, \log(w_{K})\right\} $$

(denoted also by *θ* with a slight abuse of notation). Moreover, we do not force the log-weights to sum to one – this enables moves which change only one of the weights and enhances exploration of state space.

Priors for each of the 3*K*+1 parameters in *θ*, are independent zero-centered normal distributions, with the union of their parameters denoted by *ζ*. For means, the priors are truncated to $\mathbb {R}_{+}$. For variances pf the priors *ν*_*k*_∈*ζ*:*k*=1,…,3*K*+1, in our simulations we set *ν*_*k*_=1 for all *k*.

### Count discrepancy penalty

Here, we describe (1) the estimation of the CN states of bins in each cell given a certain event tree *T* and (2) penalization of the model for the inconsistency between the estimated states and actual counts.

#### Estimation of CN states for each bin in each cell

Consider all possible bin-cell pairs, denoted (*i*,*j*), for *i*∈{1,…,*n*} and *j*∈{1,…,*m*}. We say bin *i* is contained in event *v*=(*l*_1_,*l*_2_), denoted *i*∈*v*, if *i* is larger or equal than *l*_1_ and lower than *l*_2_.

For a given tree *T* and cell attachment *σ*, consider such a bin-cell pair (*i*,*j*) for which there exists an event *v*∈an(*σ*(*j*)) that satisfies *i*∈*v*, i.e., according to the model, bin *i* has its CN changed during cell’s *j* evolutionary history. Note that since we allow events in *V*_*T*_ to overlap, there may be several such events in an(*σ*(*j*)). Denote by *v*_*F*_(*i*,*j*|*T*,*σ*) the set of all such events in an(*σ*(*j*)). In the evolutionary history of the tumor described by *T* and *σ*, *v*_*F*_(*i*,*j*|*T*,*σ*) is the set of all events that affected the CN state of bin *i* in cell *j*.

For a bin-cell pair (*i*,*j*) there may be no such vertex *v*∈an(*σ*(*j*)) that *i*∈*v*. In this case, according to *T* and *σ*, the CN state of bin *i* in cell *j* was not changed during the evolutionary history of the tumor. Such a bin has CN state equal to basal ploidy *n*_*CN*_ and we fix *v*_*F*_(*i*,*j*|*T*,*σ*)=*∅*.

Given an event tree *T* and attachment *σ*, we define a clustering $\mathcal {BC} = \bigcup {BC}_{w}$, where each cluster *BC*_*w*_ is defined by bin-cell pairs with the same *v*_*F*_(*i*,*j*|*T*,*σ*)

10 $$ {BC}_{w}= \{ (i, j) | w = v_{F}(i, j | T, \sigma) \} \text{ for } w \in \mathcal{P}(V_{T}),  $$

where $\mathcal {P}(V_{T})$ denotes the powerset of vertices. For *w*≠*∅*, *BC*_*w*_ is a set of bins that share events that changed their CN state. In contrast, *BC*_*∅*_ is the set of bins that did not have their CN state changed.

Note that for (*T*,*σ*) reflecting the true CN event history, all corrected counts in bins belonging to a given *BC*_*w*_ should be approximately equal (modulo measurement noise) and reflect the true CN in those bins. Thus, we estimate the CN in bins from *BC*_*w*_ using the average count in cluster

11 $$ \overline{w} := \frac{1}{|{BC}_{w}|}\sum\limits_{(i,j) \in {BC}_{w}} c_{j,i}.  $$

For the root node cluster *BC*_*∅*_, prior knowledge tells that $\overline {\emptyset }$ should be equal to basal ploidy *n*_*CN*_. Thus, in this special case we fix $\overline {\emptyset } = {n_{CN}}$.

#### Definition of the count discrepancy penalty

To score how a given event history fits to corrected count data we define

$$S_{(T, \sigma)} := \frac{1}{m \cdot n} \sum\limits_{w \in \mathcal{P}(V_{T})} \sum\limits_{(i,j) \in {BC}_{w}} (c_{j,i} - \overline{w})^{2} $$

The *S* score can be seen as the L2 distance between the noisy per-bin counts *C* and the CN calling results of the model.

Furthermore, we define a second score, *S*^′^ which counts the number of bins for which the average count is close to basal ploidy *n*_*CN*_. The penalty *S*^′^ is motivated by the fact that a bin with basal ploidy *n*_*CN*_ is not expected to be a result of CN evolution. Such bins could result for example from an amplification by one copy and then deletion by another copy, but such a situation, by Occam’s razor, is less likely. Thus, we define

$$S^{\prime}_{(T, \sigma)} := \frac{1}{m \cdot n} \sum\limits_{w \in \mathcal{P}(V_{T}) \setminus \emptyset} \mathbbm{1}_{\overline{w} \in [n_{CN} - 0.5, n_{CN} + 0.5)} |{BC}_{w}| $$

which penalizes events with inferred *CN*=*n*_*CN*_.

Finally, we define the count discrepancy penalty as

12 $$\begin{array}{@{}rcl@{}} R(C, D, T, \theta) = s_{1} \cdot S_{(T, \sigma^{*})} + s_{2} \cdot S^{\prime}_{(T, \sigma^{*})}, \end{array} $$

where *s*_1_ and *s*_2_ are user defined non-negative constants and *σ*^∗^ is the maximum likelihood attachment of cells calculated from (*D*,*T*,*θ*). In the case when the data *C* is not available, we set *R*(*C*,*D*,*T*,*θ*)=0. This situation occurs when the data is simulated from the generative model, as CONET only generates the count differences *D*.

### CN calling

Given an event tree *T* and the count data, we form the clustering $\mathcal {BC} = \bigcup {BC}_{w}$. The estimated CN state for each bin in each cell mapped to *BC*_*w*_ is given by the integer number that is the closest to median of *c*_*j*,*i*_ for (*i*,*j*)∈*BC*_*w*_ (median of corrected counts in bins mapping to a *BC*_*w*_). The maximum inferred CN is be set by the user.

### MCMC sampling

Our approach is to maximize the penalized *a posteriori* distribution *P*(*T*,*θ*|*D*). *P*(*T*,*θ*|*D*) is proportional to *P*(*D*|*T*,*θ*)·*P*(*θ*)·*P*(*T*) while the penalized version of the log-distribution is equal to:

13 $$ \mathcal{L}(T, \theta| C, D) = \ln(P(T, \theta|D)) + \lambda \cdot R(C, D, T, \theta)  $$

To this end, we employ a standard Metropolis-Hastings (M-H) algorithm on the joint space of event trees and mixture parameters (attachment variables have been marginalized out to decrease the size of the state space).

Moves on the state space are divided into two groups - those that change the count difference distribution parameters *θ* and those that modify the tree structure *T*. We switch between those two types in an alternating fashion. For one parameter update, we perform a fixed number of tree changing moves (in the case of our simulations this number was set to 10). This is motivated by the fact that moves on *θ* are more computationally intensive.

We use *q*(*T*^′^,*θ*^′^|*T*,*θ*) to denote the proposal kernel. Note that by the discussion above always either *T*^′^=*T* or *θ*^′^=*θ*. When it causes no confusion we omit fixed variables from the kernel, so for instance the kernel for the moves that change tree structure will be denoted by *q*(*T*^′^|*T*).

Move acceptance probability is standard and given by the expression

14 $$\begin{array}{@{}rcl@{}} \rho = \min \left(1, \frac{q(T, \theta|T^{\prime}, \theta^{\prime})}{q(T^{\prime}, \theta^{\prime}|T, \theta)} \exp({ \mathcal{L}(T^{\prime}, \theta^{\prime}|D, C) - \mathcal{L}(T, \theta|D, C))} \right). \end{array} $$

### Tree moves

We employ standard moves on the space of trees, i.e., *Prune and reattach* and it’s combinations: *Swap subtrees* and *Swap vertices on the tree*. Additionally, we employ moves that change *V*_*T*_: *Add leaf*, *Remove leaf*, *Swap event ends between the tree vertices* and *Swap vertices between* *V*_*T*_ *and* *V*_0_.

A tree of required size can be obtained from any tree by subsequent applications of *Remove leaf*, *Add leaf* moves. Structure of a tree can be adjusted by *Prune and reattach* moves. And finally any labeling can be obtained by *Swap vertices between* *V*_*T*_ *and* *V*_0_ moves. Hence our tree sampling scheme is irreducible. Aperiodicity of *Prune and reattach* assures aperiodicity of the whole chain. Below we explain the moves in detail.

#### Prune and reattach

We sample a vertex *v* uniformly from the tree and cut the edge leading to this vertex to remove the subtree rooted at *v* from the tree. Then we sample one of the remaining vertices (including the root) uniformly and attach the subtree there instead. The reverse of this move, where we again sample *v* first but then pick its old parent, has the same proposal probability since the non-descendant set has the same size each time *v* is removed. Since we can also choose the old parent when sampling a new one, this move has a non-zero probability of proposing the same tree *T*, ensuring aperiodicity. There is also a path from any tree to a tree with all vertices attached to the root, by moving each vertex to the root step by step. Via reversibility, we can likewise move from there to any other tree.

#### Swap vertices on the tree

We sample two vertices uniformly from the set *V*_*T*_∖{(0,0)} (we can not relabel the root) and exchange their positions on the tree. To reverse the move, we need to resample the same vertices. Hence, the proposal kernel is symmetric.

#### Swap subtrees

We sample two vertices *u* and *v* uniformly from the set *V*_*T*_∖{(0,0)}. If the two vertices are not in an ancestor/descendant relationship we detach them (together with their subtrees) from their parents and reattach them to each others’ former parent. Since for the reverse move we would simply need to select the same pair of vertices, this case is symmetric.

In the other case, assume *v* is a descendant of *u*. First, we cut the edge leading to *v* and move it with its subtree and attach it to the parent of *u*. Next, we detach *u* and its remaining subtree (with *v* and its descendants removed). The new parent of *u* is sampled uniformly from among *v* and all of its descendants. This assures the move to be reversible. To reverse the move, we again need to sample *u* and *v* at the start, and also to sample the previous parent of *v* from among *u* and its new (remaining) descendants. If we denote the number of descendants of *u* as *d*(*u*), the proposal probabilities now depend also on *d*(*u*) and *d*(*v*), i.e.,

15 $$\begin{array}{@{}rcl@{}} \frac{q(T|T^{\prime}) }{q(T^{\prime}|T)} =\frac{d(v) + 1}{d(u) +1}. \end{array} $$

#### Swap vertices between *V*_*T*_ and *V*_0_

We sample a vertex *u* uniformly from the set *V*_*T*_∖{(0,0)} and a vertex *v* from |*V*_0_| available inactive events. Then we transpose those vertices: we put *v* on the tree instead of *u* and move *u* to *V*_0_. To reverse the move, we again sample *v* first from |*V*_*T*_∖{(0,0)}| and *u* from |*V*_0_| available inactive events. The reverse move has the same proposal probability since both *V*_*T*_ and *V*_0_ have the same cardinalities as before the move.

#### Swap event ends between the tree vertices

Let *e*(*l*_0_,*l*_1_) denote unique event created from loci *l*_0_,*l*_1_, i.e., *e*(*l*_0_,*l*_1_) is equal to (*l*_0_,*l*_1_) if *l*_0_<*l*_1_ and to (*l*_1_,*l*_0_) otherwise.

We sample two vertices – (*l*_0_,*l*_1_),(*u*_0_,*u*_1_) from *V*_*T*_ uniformly. If the vertices represent events that belong to different chromosomes, the move is rejected. Otherwise, we have four possible ways of obtaining new events for vertices (*l*_0_,*l*_1_),(*u*_0_,*u*_1_):

- *e*(*l*_0_,*u*_0_),*e*(*l*_1_,*u*_1_),
- *e*(*l*_0_,*u*_1_),*e*(*l*_1_,*u*_0_),
- *e*(*l*_1_,*u*_1_),*e*(*l*_0_,*u*_0_),
- *e*(*l*_1_,*u*_0_),*e*(*l*_0_,*u*_1_).

We sample one of those uniformly. If both new events are valid and belong to *V*_0_ then we change (*l*_0_,*l*_1_) and (*u*_0_,*u*_1_) to new labels with probability given by (14). Otherwise, the move is rejected.

Formally, if one of the new events is either not valid or is already present on the tree then we set the likelihood *P*(*T*^′^,*θ*|*D*,*C*) of such structure to zero. This allows us to reject such a proposal deterministically since the acceptance ratio (14) is equal to zero.

This move changes both the *V*_*T*_ and *V*_0_ sets, but their cardinality remains the same. The move is symmetric.

#### Add leaf

We uniformly sample a vertex *v* from the set of inactive events *V*_0_ and add it as a leaf to uniformly sampled vertex from the *V*_*T*_ set. Denote the updated set of leaves by $\phantom {\dot {i}\!}V_{l'}$. $\phantom {\dot {i}\!}|V_{l'}|$ depends on the fact whether we added *v* as a leaf under internal vertex of the initial tree *T*, thus increasing the number of leaves, or under a leaf, leaving the number of leaves unchanged. To reverse this move, we resample *v* from $V_{l^{\prime }}$, remove *v* from the tree and add it to *V*_0_. This yields the Hastings ratio

16 $$\begin{array}{@{}rcl@{}} \frac{q(T|T^{\prime})}{q(T^{\prime}|T)} = \frac {|V_{0}||V_{T}|} {|V_{l^{\prime}}|}= \left\{\begin{array}{ll} \frac {|V_{0}||V_{T}|} {|V_{l}|}, & \text{if leaf added under a leaf} \\ \frac {|V_{0}||V_{T}|} {|V_{l}| + 1}, & \text{otherwise}. \end{array}\right. \end{array} $$

#### Remove leaf

We uniformly sample a leaf *v* from the set of leaves *V*_*l*_, remove *v* from the tree and add it to the set of inactive events *V*_0_. To reverse this move, we resample *v* from updated set of inactive events $\phantom {\dot {i}\!}V_{0'}$ ($V_{0^{\prime }} = |V_{0}| \cup v$), and add it as child to it’s former parent. To choose this parent, we sample it from the updated $V_{T^{\prime }} = V_{T} \setminus v$. Consequently, the Hastings ratio for this move becomes

17 $$\begin{array}{@{}rcl@{}} \frac{q(T|T^{\prime})}{q(T^{\prime}|T)} = \frac{|V_{l}|}{(|V_{0}|+1)(|V_{T}|-1)}. \end{array} $$

### Moves on parameter space

Recall that the vector of parameters is equal to:

$$\theta := \left(\log\left(\sigma^{2}_{0}\right), \mu_{1}, \ldots, \mu_{K}, \log\left(\sigma^{2}_{1}\right), \ldots, \log\left(\sigma^{2}_{K}\right), \log(w_{1}), \ldots, \log(w_{K}) \right). $$

We use the Metropolis-within-Gibbs algorithm, i.e. one move from *θ* to *θ*^′^ consists of changing the value of only one of the coordinates. We use the deterministic scan strategy. The coordinate, which will be updated is chosen periodically – *i*-th step proposes new value for 1+(*i* mod 3*K*+1) coordinate. For every coordinate, we use adaptive scaling random walk Metropolis kernel, where the step size is adjusted to achieve optimal acceptance probability. [74].

### Improving the convergence of MCMC sampling

To improve the convergence of our algorithm we divide the sampling scheme into two chains. We start with a chain that works on the joint space (*T*,*θ*) and outputs the estimated maximum *a posteriori* value of mixture parameters (let us denote this value by *θ*^∗^). This chain alternates between moves changing *T* and moves changing *θ*, as was described in “MCMC sampling” section. The second chain runs a fixed number *R* (this constant is user defined) of copies of the chain on the space of trees in parallel. The values of the mixture parameters are fixed and set to *θ*^∗^.

Let us describe the latter phase more precisely. Denote trees of the chains by *T*_1_,…,*T*_*R*_ and let *γ*_1_=1>*γ*_2_>…>*γ*_*R*_>0 be a sequence of *temperatures*. Chain number *i* targets distribution given by log-density

$$ \mathcal{L}_{i}(T| C, D, \theta^{*}) = \ln({P(D | T,\theta^{*})^{\gamma_{i}}} \cdot P (\theta^{*}) \cdot P(T)) + \lambda \cdot R(C, D, T, \theta^{*}). $$

Notice that the difference between the standard distribution 13 and $\mathcal {L}_{i}$ is that the likelihoods *P*(*D*|*T*,*θ*) are tempered and values of the parameters are held fixed.

One iteration of the second phase consists of two procedures:

- Each chain samples new tree $T^{\prime }_{i}$ independently using scheme from MCMC sampling,
- States of chains *J*,*J*+1 are swapped with probability $\left (\frac {P(D| T^{\prime }_{J+1},\theta ^{*})}{P(D | T^{\prime }_{J},\theta ^{*})} \right)^{\gamma _{J} - \gamma _{J+1}} $. *J* is a random index sampled uniformly from {1,…,*R*−1}.

This scheme is an example of *parallel tempering* algorithm [75] and it can be shown that its stationary distribution is proportional to $\prod _{i=1}^{R} \exp { (\mathcal {L}_{i}(T_{i}| C, D, \theta ^{*}))}$. Instead of fixing specific values for the temperatures, we utilize the adaptive Parallel Tempering algorithm of [75]. Maximum *a posteriori* tree found by the latter scheme together with *θ*^∗^ is defined to be the output of the whole procedure.

The speed of convergence of chain inferring *θ*^∗^ is highly dependent upon a good choice of initial values for *θ*. We aggregate *D* matrix into one vector and assume that it is a sample from a mixture of *K*+1 truncated normal distributions with non-negative means from which one is constrained to have mean zero. We employ the EM algorithm [76] to infer the parameters of this mixture and use them as initial values for *θ*.

### Simulations for comparative evaluation of CONET on both per-bin and per-breakpoint data

Simulations are performed to test our model’s performance in the conditions where we know the ground truth tree and both corrected counts and CN matrix, for performance evaluations in Figs. 2 and 3. For performance evaluation of CONET with different priors on the per-breakpoint data simulated directly from the model, see Additional file 1: Section S5, Figs. S13 and S14. Here, different simulation settings are generated by varying the size *t* (20 or 40 vertices) of the simulated tree *T*, the size of the simulated genome represented by the number of bins *n* (1500 for tree of size 20 or 10,000 for tree of size 40), the number of cells *m* (200 or 1000) and the level of noise in the corrected counts data matrix (low or high noise). The data is simulated as originating from one chromosome.

First, we simulate the tree structure. We assume that the tree has a linear trunk and uniformly sample the number of vertices in the trunk *t*_*r*_ from interval [0.1·*t*,0.4·*t*], and round it to integer. The branching structure of the tree below the trunk is sampled uniformly from the set of all possible trees of pre-specified size *t*−*t*_*r*_.

Next, we simulate the breakpoints and the true CN matrix. We achieve that by sampling labels for each vertex. A label (*e*,*cn*) includes information about a CN event *e* (in the form of start and end breakpoint) and its integer CN *cn*. Note that resulting labeled tree is different from CONET – the latter carries no information about CNs. In our setting *cn*∈{0,1,3,4} and breakpoints belong to one theoretical chromosome, which has either 1500 (for the tree size 20) or 10,000 (for the tree size 40) bins.

In order to obtain a realistic distribution of simulated corrected counts, we fit a mixture of five normal distributions with means fixed to (0,1,2,3,4) and unknown variances to the distribution of corrected counts from the xenograft breast cancer SA501X3F data. The estimated mixture weights for CNs (0,1,2,3,4) equal (0.02,0.2,0.68,0.05,0.038), while the respective variances equal (0.2,0.01,0.03,0.01,0.07). We denote the estimated variance of the component that corresponds to a given CN *cn* by $\sigma ^{2}_{cn}$. Likewise, weight corresponding to CN *cn* is denoted by *w*_*cn*_.

Events and CNs for each vertex are sampled independently – the former from the uniform distribution, and the latter from the categorical distribution with unnormalized weights (*w*_0_,*w*_1_,*w*_3_,*w*_4_). The resulting tree is conditioned to have no descendant events overlapping with a parent event that has CN equal to 0. It is also ensured that all parent–descendant overlapping events have different CNs.

Having the tree structure, we randomly attach cells to tree vertices. The probability of a cell being attached to a given vertex is proportional to the vertex’s depth. Cell’s attachment to the vertex with label (*e*,*cn*) defines the cell’s true CN in bins from event *e* being equal to *cn*. The number of cells is either 200 or 1000.

Finally, we generate the per-bin data in the form of the corrected counts matrix *c*_*j*,*i*_ for each cell *j*=1,…,*m* and each bin *i*=1,…,*n*. To this end, we read cell’s final CN in each bin from the tree structure and labels, and add mean zero normal noise with variance $\sigma ^{2}_{cn}$ (*low noise setting*) or $2 \cdot \sigma ^{2}_{cn}$ (*high noise setting*) where *cn* is cell’s CN in a given bin. In both settings, in addition, we introduce error to the data by sampling a random CN. To this end, for each bin, with probability 0.01, we forget the cell’s CN for that bin read off the tree structure, and sample a random one (from distribution (*w*_0_,*w*_1_,*w*_3_,*w*_4_)). Next, we use this random CN *cn* to sample corrected counts for the given bin using a zero-mean gaussian with variance either $\sigma ^{2}_{cn}$ or $2\cdot \sigma ^{2}_{cn}$, depending on the noise setting. From the resulting corrected counts matrix *c*_*j*,*i*_ we calculate the per-breakpoint data i.e. the corrected counts difference matrix *d*_*j*,*i*_.

### Model evaluation metrics for simulated data

To facilitate the assessment of the quality of inference results for simulated data we introduce nine performance scores. The scores can be divided into two groups – those that evaluate the quality of CN and breakpoint identification and those that evaluate the quality of the inferred event history.

The breakpoint identification scores are based on the comparison of the inferred breakpoint matrix to the real (simulated) one. By breakpoint matrix we mean a matrix

$$H = \left\{ h_{j,i}\right\}_{j \in \left\{1, \ldots,m\right\}, i \in \left\{1, \ldots, n\right\}} $$

where *h*_*j*,*i*_ is equal to 1 if bin *i* has a different CN then its leftmost neighbor for cell *j* and 0 otherwise.

*CN-RMSE* – root mean squared difference between real and inferred CN matrices.

*False positive rate* – number of entries equal to 1 from inferred breakpoint matrix which are equal to 0 in the real matrix divided by the total sum of entries of the inferred matrix. False positive rate ranges from 0 to 1.

*False negative rate* – number of entries equal to 0 from inferred breakpoint matrix which are equal to 1 in the real matrix divided by the total sum of entries of the real matrix. False negative rate ranges from 0 to 1.

*Symmetric distance* – *L*_1_ distance between real and inferred breakpoint matrices divided by the number of cells. Notice that although *FP*,*FN* are divided by the total number of breakpoints, this one is not. As a result values of SD may exceed 1.0. For instance, *SD*=1 indicates that on average one breakpoint is missed or wrongly inferred for each cell.

Lower values of *CN-RMSE*, *false positive rate*, *false negative rate* and *symmetric distance* indicate better CN and breakpoint identification.

The event history scores facilitate the comparison of the inferred tree structure to the real (simulated) structure and consist of:

*Tree size* – the size of the inferred tree |*T*|, i.e. the number of vertices including root. In the evaluation, this number is compared to the real tree size.

*Event sensitivity* – the proportion of the inferred events that are present in the real tree.

*Event precision* – the proportion of real tree events that are present in the inferred tree.

*Edge sensitivity* – the proportion of the inferred tree edges that are present in the real tree.

*Edge precision* – the proportion of real tree edges that are present in the inferred tree.

The values of event sensitivity, event precision, edge sensitivity and edge precision range between 0 and 1, where larger values indicate better inference of the event history.

*Ancestry recall* - Let $\mathcal {A}(T, \sigma)$ denote all pairs of cells (*i*,*j*) such that *σ*(*i*) is an ancestor of *σ*(*j*) in *T*. Ancestry recall is defined as $\frac {|\mathcal {A}(T, \sigma) \cap \mathcal {A}(T_{real}, \sigma _{real})|}{ |\mathcal {A}(T_{real}, \sigma _{real})|}$, where (*T*,*σ*) is an inferred tree and (*T*_*real*_,*σ*_*real*_) is a real tree.

*Branching recall* - Let $\mathcal {B}(T, \sigma)$ denote all pairs of cells (*i*,*j*) such that *σ*(*i*) is not an ancestor or descendant of *σ*(*j*) in *T*. Branching recall is defined as $\frac {|\mathcal {B}(T, \sigma) \cap \mathcal {B}(T_{real}, \sigma _{real})|}{ |\mathcal {B}(T_{real}, \sigma _{real})|}$, where (*T*,*σ*) is an inferred tree and (*T*_*real*_,*σ*_*real*_) is a real tree.

*Rand index* - fraction of cell pairs (*i*,*j*) that are clustered correctly by the inferred tree (*T*,*σ*). A pair (*i*,*j*) is clustered correctly if either:

*σ*(*i*)=*σ*(*j*) and *σ*_*real*_(*i*)=*σ*_*real*_(*j*)

or:

*σ*(*i*)≠*σ*(*j*) and *σ*_*real*_(*i*)≠*σ*_*real*_(*j*).

## Supplementary Information

**Additional file 1** Supplementary information. It includes CONET run settings used in the presented results (Section S1), the run settings for the compared methods (Section S2), the comparison of MEDALT trees on different CN calling input for the xenograft breast cancer SA501X3F data (Section S3), the comparison of event discovery for short and long events (Section S4), the performance of CONET using different priors on per-breakpoint data simulated from the model (Section S5), the evaluation of the count discrepancy penalty for scDNA-seq data sample (Section S6), a recommended procedure for setting CONET regularization parameters (Section S7) and Figs. S1–S14.

**Additional file 2** List of the inferred CONET vertices for xenograft SA501X3F breast cancer sample.

**Additional file 3** List of the inferred CONET vertices for TN2 invasive ductal carcinoma sample.

**Additional file 4** Table S1 Assessment of CONET accuracy and computational time for increasing number of cells.

**Additional file 5** Table S2 Average estimated parameters for different versions of CONET on synthetic data with high noise, for runs shown in Fig. S12.

**Additional file 6** Table S3 Assessment of CONET inference for biological data.

**Additional file 7** Table S4 Assessment of CN calling for biological data.

**Additional file 8** Review history.

## Data Availability

CONET implementation is freely available under CC-BY-NC 4.0 International license on Github (https://github.com/szczurek-lab/CONET)[77] and Zenodo (DOI:10.5281/zenodo.5786319) [78]. SA501X3F sample is deposited under accession number EGAD00001003149 and SA501X4F sample is deposited under accession number EGAD00001003150. Both were published in [49]. TN2 sample is deposited under accession number PRJNA629885 and was published in [51].
