# Supplementary material for: CONET: copy number event tree model of evolutionary tumor history for single-cell data
Source: Genome Biol. 2022 Jun 9;23:128. doi: 10.1186/s13059-022-02693-z (PMC9185904; doi:10.1186/s13059-022-02693-z)
Supplement: Supplementary file 1 — Additional file 1 Supplementary information. It includes CONET run settings used in the presented results (Section S1), the run settings for the compared methods (Section S2), the comparison of MEDALT trees on different CN calling input for the xenograft breast cancer SA501X3F data (Section S3), the comparison of event discovery for short and long events (Section S4), the performance of CONET using different priors on per-breakpoint data simulated from the model (Section S5), the evaluation of the count discrepancy penalty for scDNA-seq data sample (Section S6), a recommended procedure for setting CONET regularization parameters (Section S7) and Figs. S1–S14. [file 13059_2022_2693_MOESM1_ESM.pdf]

RESEARCH

CONET: copy number event tree model of evolutionary tumor history for single-cell data Supplementary Information

Magda Markowska[1,2†], Tomasz Cąkała[1†], Błażej Miasojedow[1], Bogac Aybey[3], Dilafruz Juraeva[3], Johanna Mazur[3], Edith Ross[3], Eike Staub[3] and Ewa Szczurek[1*]

*Correspondence: szczurek@mimuw.edu.pl
[1]University of Warsaw, Faculty of Mathematics, Informatics and Mechanics, Banacha 2, Warsaw, Poland
Full list of author information is available at the end of the article
†Equal contributor

Additional File 1

Contents

S1 CONET run settings used in the presented results **3**

S2 Run settings for the compared methods **3**

S3 Comparison of MEDALT trees on different CN calling input for the xenograft breast cancer SA501X3F data **4**

S4 Comparison of event discovery for short and long events **4**

S5 Performance of CONET using different priors on per-breakpoint data simulated from the model **5**
S5.1 Data simulation from the model 7

S6 Evaluation of the count discrepancy penalty for scDNA-seq data sample **8**
S6.1 Applied quality measures 9
S6.2 Assessment of CONET quality 9
S6.3 Assessment of CN calling quality 10

S7 Recommended procedure for setting CONET regularization parameters **11**

List of Figures

S1 The fit of the assumed theoretical and the empirical data distributions, for data generated using two different scDNA-seq technologies. 13
S2 Assessment of CC-rounding CN calling for synthetic data 14
S3 Graphical illustration of CN calling results with CBS+MergeLevels for SA501X3F xenograft breast cancer data set. 15
S4 Graphical illustration of CN calling results with rounding for SA501X3F xenograft breast cancer data set 16

S5 MEDALT trees. 17
S6 Comparison of CONETs for mixed SA501X3F and SA501X4F vs single SA501X3F xenograft breast cancer samples. 18
S7 CONET for TN2 invasive ductal carcinoma data set 19
S8 Graphical illustration of CN calling results for for TN2 invasive ductal carcinoma data set. 20
S9 Assessment of CONET recovery of ancestry relations and clustering of cells. 21
S10 Effectiveness of CONET on short versus long copy number events. . 22
S11 Log-likelihood trace plots for eight runs of CONET on Xenograft breast cancer data. 22
S12 Log-likelihood trace plots of parameters inference chain for different versions of CONET on synthetic data with high noise level. 23
S13 Assessment of the tree structure inference for simulated data tests results. 24
S14 Assessment of breakpoints inference for simulated data tests results. 25

S1 CONET run settings used in the presented results

For all runs on simulated data presented in Section Performance of CONET in comparison to other methods on simulated data in the main text, the run parameters of CONET are fixed to the following values:

$$(k_0 = 1, k_1 = 0.01, s_1 = 100000, s_2 = 100000, n_{CN} = 2).$$

For all runs on simulated data presented in Section Performance of CONET using different priors on per-breakpoint data simulated from the model, the run parameters of CONET are fixed to the following values:

- $(k_0 = 1, k_1 = 0.5, s_1 = 0, s_2 = 0, n_{CN} = 2)$ for *Tree structure prior* equal to 0.5 setting (cell attachment probability is uniform),
- $(k_0 = 1, k_1 = 0.1, s_1 = 0, s_2 = 0, n_{CN} = 2)$ for *Tree structure prior* equal to 0.1 setting (cell attachment probability is uniform),
- $(k_0 = 1, k_1 = 0, s_1 = 0, s_2 = 0, n_{CN} = 2)$ for *Attachment prior* equal to 0.5 setting.

For the evaluation of CONET scalability on simulated data with increasingly large m (number of cells), presented in Table S1 (Additional File 4), the run parameters of CONET are fixed to:

$$(k_0 = 1, k_1 = 0.01 \cdot \frac{500}{m}, s_1 = 100000, s_2 = 100000, n_{CN} = 2).$$

For the SA501X3F xenograft breast cancer sample the run settings were:

$$(k_0 = 1, k_1 = 0.5, s_1 = 200000, s_2 = 200000, n_{CN} = 2).$$

For the joint SA501X3F and SA501X4F xenograft breast cancer sample the run settings were:

$$(k_0 = 1, k_1 = 0.005, s_1 = 200000, s_2 = 200000, n_{CN} = 2).$$

For the TN2 invasive ductal carcinoma sample the run settings were:

$$(k_0 = 1, k_1 = 0.1, s_1 = 1000000, s_2 = 1000000, n_{CN} = 3).$$

S2 Run settings for the compared methods

HMMCopy was run with the command *HMMSegment* from R "HMMcopy" package with *maxiter* set to 50000.

CBS+Megelevels were run using the following commands:

- *segment(smooth.CNA(·), verbose=0, alpha = 0.01, undo.prune = 0.05)*,
- followed by *mergeLevels* (after appropriate transformation of results from the preceding command).

using R "DNAcopy", "aCGH" packages.

SCICoNE was run with default parameters provided by the implementation with the exception of *window_size* which was set to 10.

MEDALT was run with default parameters provided by the implementation.

S3 Comparison of MEDALT trees on different CN calling input for the xenograft breast cancer SA501X3F data

Figure S5 shows comparison of MEDALT trees obtained from different CN matrices inferred by CBS+MergeLevels, HMMCopy and CONET. For input from CBC+MergeLevels, the CN calling procedure that is the default for MEDALT, the event tree (Figure S5A) is the largest and has multiple branches. Still, the majority of cells are assigned to one node, which includes alteration of *AKT3* and *ATR*. For this input, the events recognized by MEDALT as significant affect only two breast cancer genes, *AKT1* and *NTRK3* (according to the COSMIC Cancer Gene Census [67]). For the input CNs called using HMMCopy, the MEDALT tree is much smaller, and contains a single node which aggregates the majority of cells (Figure S5B). Among the events associated with this largest node the alterations of *AKT3* and *ATR* occur again, similarly to the tree for the CBC+MergeLevels input. Other events, however, do not agree between the two trees: either the events from one tree do not occur in the other, or it occurs in a different order implied by the tree structures. For the HMMCopy input, MEDALT identifies events affecting 9 breast cancer genes, two of which overlap with the breast cancer genes identified for the CBC+Mergelevels input. Finally, for the input provided by CONET, the tree structure and event occurrence and ordering is again different from the other two trees (figure S5C). This tree, however, is characterized by a more even distribution of cells assigned to different nodes of the tree. This tree contains events affecting four genes, two of which overlap with the other ones (*AKT1* and *NTRK3*).

S4 Comparison of event discovery for short and long events

To assess the effectiveness of CONET on different scales of CNAs, we compare event precision and sensitivity on short and long events, for CONET with three different sets of candidate breakpoints loci: i) CONET (known), with true candidate breakpoint loci set, ii) CONET (HMMCopy), with candidate breakpoint loci set identified by HMMCopy and iii) CONET (CBS+MergeLevels), with candidate breakpoint loci set identified by CBS followed by MergeLevels (Figure S10). The experiment is performed for all 50 synthetic data sets generated in the most challenging setting (tree of size 40 and 1000 cells; see Section Simulations for comparative evaluation of CONET on both per-bin and per-breakpoint data in the main text). An event is considered short if its length is smaller than 0.25th quantile of event length for all events from the ground truth tree. Likewise, an event is considered long if its length is greater than 0.75th quantile of event length for all events from the ground truth tree.

For CONET (known), event precision is very high and larger for short events than for long events. This can be due to the fact that the model prioritizes shorter events (see the description of P_{el} prior in Section Tree structure priors in the main text). In contrast, for CONET (HMMCopy) and CONET (CBS+MergeLevels) event precision is lower for short events than for long events. We speculate that this is due to the fact that HMMCopy and CBS+MergeLevels are worse at detecting breakpoints for short events and fail to provide them as input candidate loci.

For CONET (known) and CONET (CBS+MergeLevels) event sensitivity does not show differences between long and short events. For CONET (HMMCopy) the median sensitivity for long events is around 10 percentage points higher.

Overall CONET obtains satisfactory results for both short and long CN events detection. The above mentioned differences do not show systematic bias for the event length and result from the method of candidate breakpoint loci detection.

S5 Performance of CONET using different priors on per-breakpoint data simulated from the model

To assess the performance of CONET using different priors in a setting where the ground truth is known, we conduct the evaluation on simulated data. To this end, we use CONET as a generative model, and sample trees of given sizes with attached predefined numbers of cells, outputting per-breakpoint data in the form of absolute count difference matrices, according to the assumed absolute count difference distributions (Section Data simulation from the model). Note that this simulation experiment differs in several ways from the experiment on simulated data presented in the main text. First, the data is generated directly from the model, and thus the performance of the model in terms of learning the trees and recovering the breakpoints depends exclusively on the level of noise put into the simulated data in a controlled manner. Second, only the per-breakpoint data are simulated, and not the per-bin data, and thus no other existing approach can be applied. Therefore, in this simulation experiment we do not compare CONET to any other method. Instead, we evaluate the performance of CONET inference from per-breakpoint data alone, for different types of priors.

The simulated count difference matrix is used as an input for CONET inference procedure and the results are compared with the ground truth to assess the quality of the output tree structure and breakpoints in each cell, according to cell attachment which maximizes the likelihood.

Each evaluation scenario is described by three parameters - the size of the CONET tree (with values from $\{20, 40\}$), the number of cells (with values from $\{200, 400, 1000, 2000\}$) which are randomly assigned to tree vertices during simulation, and finally the absolute count difference distributions that will be used for the generation of the difference matrices. The number of potential breakpoint loci is fixed to twice the tree size.

Entries of the difference matrix are sampled from two corrected counts absolute difference distributions settings - *well separated* and *poorly separated*. The first one provides a clear distinction between the distribution of the absolute count differences at breakpoint loci and the differences at loci without breakpoints. The well separated setting corresponds to higher quality data, with less noise and higher coverage. The poorly separated setting represents input data with more noise and as such is expected to be more challenging for our algorithm. During the inference procedure, the distributions of the corrected count absolute differences are assumed to be unknown and must be inferred by our algorithm.

Additionally, we run our algorithm with two different choices of priors. The *tree structure prior* penalizes inference of large trees. The impact of this penalty depends on the constant which is set by the user, and we evaluate this prior with the constant set either to 0.1 or to 0.5. The *attachment prior* encourages our scheme to propose trees that attach cells to nodes with a history consisting of shorter events.

For each of the scenarios described above (number of cells, tree size, distributions setting, prior) we generate 10 random models. For each of the generated models, we

run our inference scheme 10 times (each time with a different seed) with $5 \cdot 10^5$ steps for parameter inference and 10^6 MCMC steps, obtaining 10 inferred CONETs and 10 breakpoint matrices (information about breakpoints in each cell according to their maximum attachment to the tree). The average running time of one inference procedure ranges from less than two minutes in the least computationally demanding scenario (tree size 20 with 200 cells) to half an hour in the most demanding scenario (tree size 40 with 2000 cells). We then compare the inferred data to the ground truth information from the simulated models. This strategy allows us to evaluate not only the quality of a single prediction, but also the consistency of the results across different runs of the algorithm for common input data.

The quality of inference results was evaluated using the same metrics as introduced in the main text: *Inferred Tree Size*, *Edge Sensitivity*, *Edge Precision* for assessing the quality of the inferred CN event tree, *False Positive Rate* and *False Negative rate* quantifying the similarity of the inferred tree's edge set to that of the real tree, and *Symmetric Distance* assessing the quality of breakpoint detection.

Figure S13 depicts aggregated tree scores for the analyzed simulation scenarios and demonstrates the high performance of the model in terms of inferring CONET structure. The sizes of the inferred trees oscillate around the real values of 20 or 40, with slight over- or under-prediction being dependent on the choice of prior (Figure S13 A–D). There is a tendency, however, that the trees grow as the number of cells increases. This is to be expected, since for a higher amount of cells the algorithm has more possibilities of increasing the likelihood by adding subtrees that correspond to small subpopulations of our cells. All evaluated priors regularize the model and are effective at limiting the inferred tree growth. For the tree structure prior, the results depend on the constant controlling the prior's strength. The more complex scenarios with more cells and bigger true trees, the strong tree structure prior (constant 0.5) sometimes over-penalizes the tree size, resulting in overly small trees. In comparison to the strong tree structure prior, the moderate tree structure prior (constant equal to 0.1) results in trees of size closer to the true values. The attachment prior works in the most subtle way i.e. the model returns the biggest trees compared to the two other priors.

Edge sensitivity (Figure S13 E–H) decreases with stronger regularization due to smaller tree size, but is very high for adequate regularization choice. In simulation scenarios, the strong tree structure prior gives the smallest edge sensitivity. For trees of size 20 and the case of the well separated distributions (Figure S13 E), both the attachment prior and moderate tree structure prior yield a high edge sensitivity of around 0.75, regardless of the number of cells. For the same tree size and the poorly separated case, edge sensitivity decreases by around 0.1 for these priors (Figure S13 F). For larger trees (Figure S13 G,H) edge sensitivity further decreases, but it grows with larger numbers of cells. In the most difficult of the analyzed simulation scenarios (tree size 40 and poorly separated data), with 2000 cells, the attachment and moderate tree structure prior give edge sensitivity of around 0.65.

Compared to edge sensitivity, the different regularization priors have less effect on edge precision (Figure S13 I-L). In all analyzed simulation scenarios, the majority of discovered edges is contained in the real history. In the simplest scenario (tree

size 20 and well separated data, Figure S13 I), edge precision is around 0.75 for all cell numbers. Even in the most difficult scenario (Figure S13 L), the median edge precision of the algorithm exceeds 0.5, regardless of the prior and the cell count. In some simulation scenarios (Figure S13 I,J), edge precision decreases with the increasing number of cells for the attachment prior. This is because with this prior, larger trees are inferred.

Figure S14 illustrates the excellent performance of breakpoint detection using our algorithm. In all analyzed scenarios, the median false positive rate of the detected breakpoints is very low. Indeed, regardless of the choice of the prior and the number of cells, the median false positive rate is below 0.1 (Figure S14 A–D). In the well separated data scenarios (Figure S14 A,C), the false positive rates are even lower (median less than 0.05).

Similarly, the false negative rate is very low in all scenarios (Figure S14 E–H). Only in the most difficult simulation scenario (tree size 40 and poorly separated data) and only for the strong tree structure prior (constant 0.5), the median false negative rate exceeds 0.1. Better data separability (Figure S14 E,G) yields even better results, with median false negative rates below 0.025. Transition to poorly separated distribution results in a more significant deterioration of the false negative rate than of the false positive rate – this is an outcome of more pronounced underestimation of tree size in those scenarios (Figure S13).

Interestingly, both false positive and false negative breakpoint rates are very small for the attachment prior, also in the scenarios with small trees, where attachment prior yielded relatively low edge precision (Figure S13 I,J) and proposed trees were larger than the true trees (Figure S13 A,B). This result suggests that even though edge precision may be worse for CONET with priors that propose trees that are too large, the algorithm can still exhibit good breakpoint detection.

The excellent performance in breakpoint re-detection for simulated data is best quantified using the symmetric difference metric (Figure S14 I–L). Again, even in the most difficult scenario (Figure S14 L), the symmetric distance between real and the inferred breakpoint matrices is only around 1. This means that on average every cell has only a single missed or wrongly inferred breakpoint.

In conclusion, although the best results are achieved for the smaller trees with well separated absolute count difference distributions, overall the algorithm excels in breakpoint detection across all evaluated measures, while the correct choice of regularization ensures satisfactory edge sensitivity and precision.

S5.1 Data simulation from the model

Simulations are performed to test CONET performance in the conditions where we know the ground truth CONET tree. Different simulation settings are generated, by varying the size t of the simulated tree T, the number of loci $|L|$, the number of single cells m and whether the distribution of the corrected count absolute differences for the breakpoint loci is well separated from the distribution for the non-breakpoint loci (*well separated setting*) or not (*poorly separated setting*). The data is simulated as originating from one chromosome.

First, the tree structure T is sampled uniformly from the set of all trees of prespecified size t. The size t is either 20 with the number of all possible breakpoints $|L|$

equal to 40 or two times more, i.e., 40 with 80 possible breakpoints. Next, we sample the events corresponding to each vertex from the set of all possible events with log-probability proportional to the negative length of the event. The probability of a cell being attached to a given vertex v is equal to α_v^*, where $(\alpha_v^*)_{v \in V(T)}$ is sampled from Dirichlet($\mathbb{1}$).

Finally, we generate simulated corrected count absolute differences $d_{j,i}$ for each cell $j = 1, \ldots, m$ and each locus $i \in L$. To this end, for each cell, its breakpoints are read from its tree attachment. Differences for loci without breakpoint are sampled from distribution f_0 while differences for loci with breakpoint are sampled from distribution f_{bp}. In the *well separated setting* case we set:

- $f_0 = \mathcal{N}(0, 0.3)$
- f_{bp} is a mixture of $(\mathcal{N}(1, 0.4), \mathcal{N}(2, 0.4), \mathcal{N}(3, 1.7))$ with weights $(0.5, 0.35, 0.15)$.

In the *poorly separated setting* case we set:

- $f_0 = \mathcal{N}(0, 0.7)$
- f_{bp} is a mixture of $(\mathcal{N}(1, 0.7), \mathcal{N}(2, 0.7), \mathcal{N}(3, 1.7))$ with weights $(0.5, 0.35, 0.15)$.

Where $\mathcal{N}(a, b)$ denotes normal distribution with mean a and standard deviation b. In the simulations we do not produce corrected count data C and thus set the s_0 constant to 0, i.e., the $R(C, D, T, \theta)$ penalty is ignored in the inference.

S6 Evaluation of the count discrepancy penalty for scDNA-seq data sample

In the case of a real scDNA-seq data set, where the per-bin corrected count data is available, it is advantageous to apply additional regularisation in the form of the count discrepancy penalty. The count discrepancy penalty consists of two terms (Methods). The first term corresponds to the L_2 distance between the noisy counts in the data and the CN estimation based on the model. The weight of this term is controlled by a constant s_1, with $s_1 = 0$ corresponding to the fact that this term is not included in the penalty. The second term penalizes trees that create regions changed by CN events and inferred CN equal to two. The weight of this term is controlled by the constant s_2.

To demonstrate the benefit of applying the discrepanccy penalty, we consider three scenarios. In the first scenario, similarly to the tests on simulated data, we do not apply the count discrepancy penalty at all ($s_1 = s_2 = 0$). In the second scenario we apply only the first penalty ($s_1 = 200000$, $s_2 = 0$). Finally, in the third scenario we take advantage of both count discrepancy penalties ($s_1 = s_2 = 200000$). In each case, CONET inference is performed under the same MCMC sampling setting): 0.5 million iterations with joint inference of the tree structure and the corrected count absolute difference distributions, proceeded with 1 million iterations with only tree moves (see Section Methods in the main text).

The run time for the first scenario is 2.5 hours, the second scenario - 11.5 hours, and the third - just under 10 hours (on a high performance computer with AMD Ryzen Threadripper 3990X 64-Core CPU and 128 GB RAM, using 5 threads). For each scenario, the run times for the whole inference procedures in the case of biological data are longer than for simulated data, since we deal with over ten times more potential breakpoints (which translates to around 100 times more potential CN events). The first scenario is substantially the least computationally demanding

because it skips the count discrepancy calculation in each MCMC step and - as a result - infers much smaller trees (tens vs hundreds of vertices). The second scenario runs longer than the third because the inferred trees are larger when we do not penalize trees for inferring regions with CN equal to two.

S6.1 Applied quality measures

The CONETs obtained in the three scenarios are evaluated using several quality measures.

For basic characterization of the inferred trees we report *Tree size* (the number of CN events inferred for a given data set) and *No of clusters* – the number of all BC_w clusters i.e. subsets of bins that share the same copy number event history, including the $B_\emptyset$ cluster.

To evaluate the consistency of an inferred CONET with the count data, we use characteristics defined below. Cluster support is calculated as the number of bins in a given cluster with corrected count close to inferred CN ($c \in [CN - 0.5, CN + 0.5]$) divided by the number of all the bins in the cluster.

Avg cluster support – average over all BC_w clusters' supports,

Perc of good clusters – percentage of clusters with at least 0.7 support,

Perc of n_{CN} *clusters* – percentage of clusters that infer CN equal to basal ploidy n_{CN} (not including $B_\emptyset$ cluster).

Finally, we define detailed measures evaluating the quality and consistency of the CN calling results, where by bins inside events we mean bins in all BC_w clusters except for $BC_\emptyset$.

Avg Gini Index in events – Gini Index calculated separately for corrected counts in bins for each BC_w cluster ($w \neq \emptyset$), averaged over the clusters,

Avg Entropy in events – normalized Shannon Entropy calculated separately for corrected counts in bins in each BC_w cluster ($w \neq \emptyset$), averaged over the clusters; to calculate the entropy in each cluster the corrected counts are grouped into intervals of width 1, concentrated around integer copy numbers,

Gini Index outside events – Gini Index calculated for corrected counts in bins in the $BC_\emptyset$ cluster,

Entropy outside events – normalized Shannon Entropy calculated for corrected counts in bins in the $BC_\emptyset$ cluster,

CN-TPR – The number of bins inside events with corrected count c far from basal ploidy, i.e. $c \notin [n_{CN} - 0.5, n_{CN} + 0.5]$, divided by the total number of bins with the true corrected count far from basal ploidy,

CN-FPR – The number of bins inside events with corrected count c close basal ploidy, i.e. $c \in [n_{CN} - 0.5, n_{CN} + 0.5]$ divided by the total number of bins with the true corrected count close to two.

CNCC-RMSE – root mean square error for CN calling for experimental data with unkown true CN, the quadratic mean of the differences between the inferred integer copy number for each bin and the corrected count for each corresponding bin.

S6.2 Assessment of CONET quality

The additional count discrepancy improves the quality of the inferred tree (Additional File 6: Table S3). In the first scenario, the tree inferred without the penalty

contains only 35 events and defines 127 clusters. It is hard to expect that such a small tree can fully explain the CN variation in 260 single tumor cells. Correspondingly, according to all quality measures, this tree has the lowest quality of clusters, compared to the trees obtained with the additional count discrepancy penalty. The tree obtained in the second scenario is noticeably larger, and although the cluster qualities are comparable to the third scenario, the percentage of clusters with CN equal to two is too high. The comparison clearly demonstrates the overall advantage of applying the full count discrepancy penalty (scenario 3), which yields the CONET of the average size and combines the good quality of nodes with low percentage of clusters that infer CN equal to basal ploidy 2. The fact that the inference without the additional penalty (the first scenario) gives very good performance on simulated data, shows that the real biological data poses a significantly more difficult challenge for the model.

S6.3 Assessment of CN calling quality

Similarly, incorporation of the count discrepancy penalty improves CN estimation (Methods; Additional File 7: Table S4). As above, the evaluation is based on the quality of bin clusters (sets of bins that share the same event history according to the model). Here, we first consider bin clusters that are indeed changed by events of the tree, referred to as *in events*. Second, we consider bins that are in one large cluster of all bins that were not included in the events of the tree, i.e. the tree does not infer any CN tree for these bins. These bins are referred to as *outside events*. To quantify the dispersion of corrected counts in both the bin clusters inside and outside events, we use the Gini index and Shanon Entropy measures. Next, CN true positive rate (CN-TPR) is computed as the fraction of bins with true corrected count not rounding to two and contained in events of a CONET, out of all bins with true corrected count not rounding to two. Here, we consider the bins with true corrected count not rounding to two as the positive examples of bins that truly underwent a CN change event. The CN false positive rate (CN-FPR) quantifies the fraction of bins that are part of a CONET events (the bins that underwent a CN event according to our model) out of the total number of bins with corrected count rounding to two (the bins that did not undergo a CN event according to the data). Root mean square error for our CN calling procedure (CNCC-RMSE) is calculated as the quadratic mean of the differences between inferred integer CN and corrected count in each bin, i.e. the square root of the count discrepancy penalty. For a correctly inferred CONET, the CNCC-RMSE reflects the noisiness of scDNA-seq data.

The small size of the CONET inferred without the count discrepancy penalty in the first scenario, results in bad overall quality of inferred CNs according to all quality measures. Compared to the trees inferred with the penalty, this tree has higher dispersion inside events and - more apparently - outside events, very low CN-TPR and almost two times higher CNCC-RMSE. In the second scenario an opposite situation occurs, where the CN-TPR is close to 100% because this most complicated CONET includes such a high number of events that almost all bins with CN far from two are included inside them. The dispersion measures improve substantially compared to scenario 1 and the CNCC-RMSE is the lowest. This

happens at the cost of CN-FPR, which is over three times higher than in the first scenario and nine times higher than in the third scenario. This again proves that without penalizing inference of CN equal to two inside events, the inferred tree grows too large. The CN calling results obtained using the CONET with the full discrepancy penalty in the third scenario are by far the best, constituting a well balanced compromise between the too simple and too complicated CONETs from the two other scenarios. The quality of CN calling results for the tree in scenario 3 is higher than for the first scenario across all parameters. In comparison to the tree obtained in scenario 2, this tree is similar in terms of low dispersion in counts in bins inside and outside events, rare inclusion of bins with corrected count far from two in CN events (CN-TPR) and low CN calling error (CNCC-RMSE), while scoring far better in terms of restricting the inclusion of bins with corrected count close to two (CN-FPR).

We conclude that additional regularisation in the form of the full count discrepancy penalty is necessary when dealing with noisy, low-depth scDNA-seq data from real experiments. The degree of this regularisation should be calibrated to the specific biological data set with scDNA-seq technology in mind, utilising different s_1 and s_2 count discrepancy constants' values and comparing the results with the aid of the described quality measures.

Author details
[1]University of Warsaw, Faculty of Mathematics, Informatics and Mechanics, Banacha 2, Warsaw, Poland. [2]Medical University of Warsaw, Postgraduate School of Molecular Medicine, Ks. Trojdena 2a Street, Warsaw, Poland. [3]Merck Healthcare KGaA, Translational Medicine, Oncology Bioinformatics, Frankfurter Str. 250, 64293 Darmstadt, Germany.

References

S7 Recommended procedure for setting CONET regularization parameters

Regularization parameters can be used to control the output tree size, as well as the fit of the output CN calls to the input per-bin data. Copy number events that occur in high frequency across cells are localized in vertices at the top of the tree (close to the trunk). Larger trees will explain rarer CN events, to the extent that very large trees can already include events that correspond to the noise in the data. Therefore, manipulating the regularization parameters helps to avoid over-fitting.

To identify optimal regularization parameter values, we recommend running the model for different regularization parameter settings and choosing those that yield the best tree quality measures, as introduced in section Applied quality measures. Specifically, in the initial run, set the values for the model regularization parameters:

$$(k_1 = 0.0, s_1 = 100000, s_2 = 100000, k_0 = 1).$$

After finishing the inference procedure, calculate the quality measures defined in Applied quality measures using the available *readTree.R* script. Compare obtained values to those described in Section Assessment of CONET quality and presented in Tables S3 and S4 (Additional Files 6 and 7). If the trees are too large and over-fit to the data, which can be indicated by a large value of $CN - FPR$, fit the data size prior k_1 i.e. run a series of models with different values $k_1 \in \{0.01, 0.05, 0.1, 0.2, 0.5\}$, calculate and compare the quality measures. Higher k_1

values generally decrease the size of inferred CONET. After setting optimal k_1, perform fine tuning by checking results for different values of count penalty constants $s_1 = s_2 \in \{50000, 200000, 500000, 1000000\}$. Higher s_1 and s_2 values generally increase the size of inferred CONET. To make sure that the regularization parameter values are optimal, additional runs can be performed in the relative proximity of the regularization parameters value that returned the best inference results.

Supplementary Figures

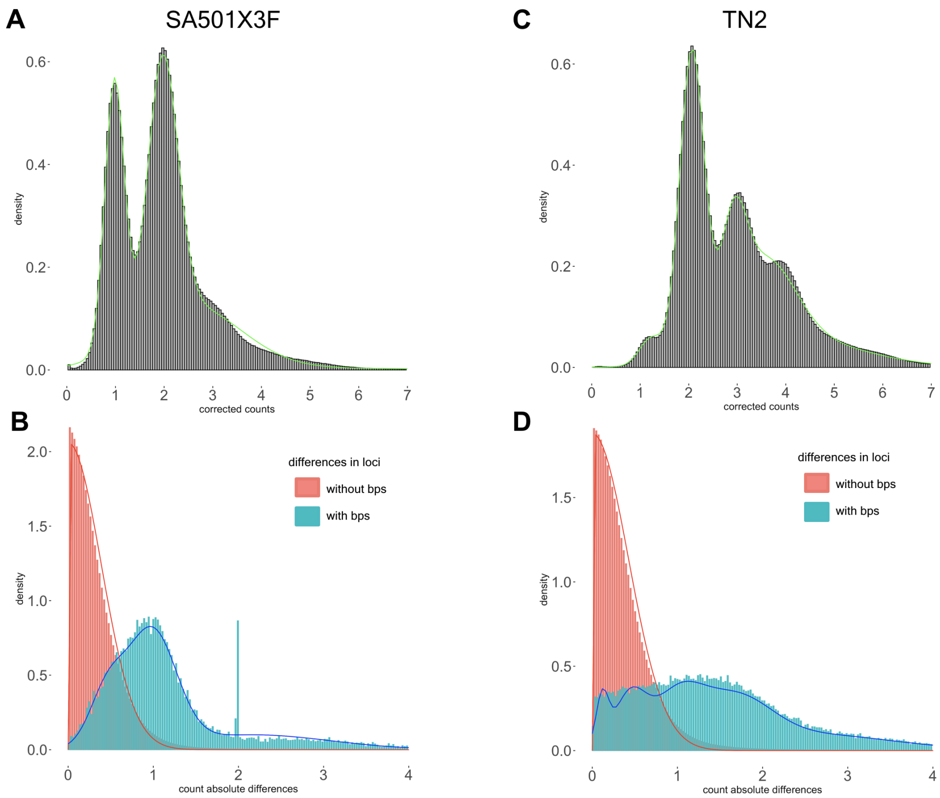

Figure S1 The fit of the assumed theoretical and the empirical data distributions, for data generated using two different scDNA-seq technologies. A Density histogram (y axis) of corrected counts (x axis) for SA501X3F experimental data set, generated using DLP protocol. Green line represents fitted mixed normal distribution with 4 components. **B** Double density histogram (y axis) of count absolute differences (x axis) for SA501X3F experimental data set. Red line represents fitted truncated normal distribution and blue line - fitted mixed normal distribution (4 components). **C** Density histogram (y axis) of corrected counts (x axis) for TN2 experimental data set, generated using the ACT protocol. Green line represents fitted mixed normal distribution with 6 components. **D** Double density histogram (y axis) of count absolute differences (x axis) for TN2 experimental data set. Red line represents fitted truncated normal distribution and blue line - fitted mixed normal distribution (6 components). For both datasets, the assumed theoretical distributions fit the data perfectly.

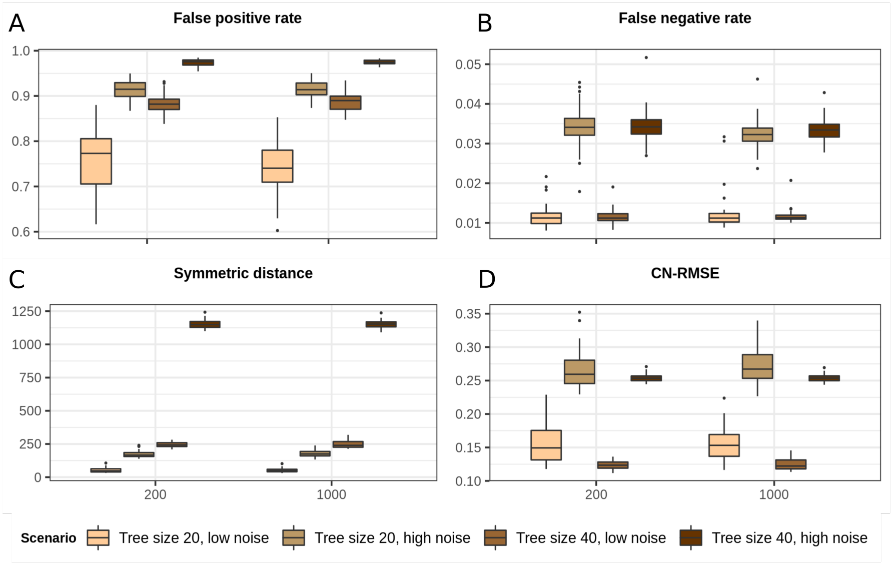

Figure S2 Assessment of CC-rounding CN calling for synthetic data A Distribution of false positive rate (y axis) as a function of cell count (x axis) for all simulation scenarios. **B** Distribution of false negative rate (y axis) as a function of cell count (x axis) for all simulation scenarios. **C** Distribution of symmetric distance (y axis) as a function of cell count (x axis) for all simulation scenarios. **D** Distribution of CN-RMSE (y axis) as a function of cell count (x axis) for all simulation scenarios. Overall results are very poor for all scenarios indicating very high sensitivity to noise in the data.

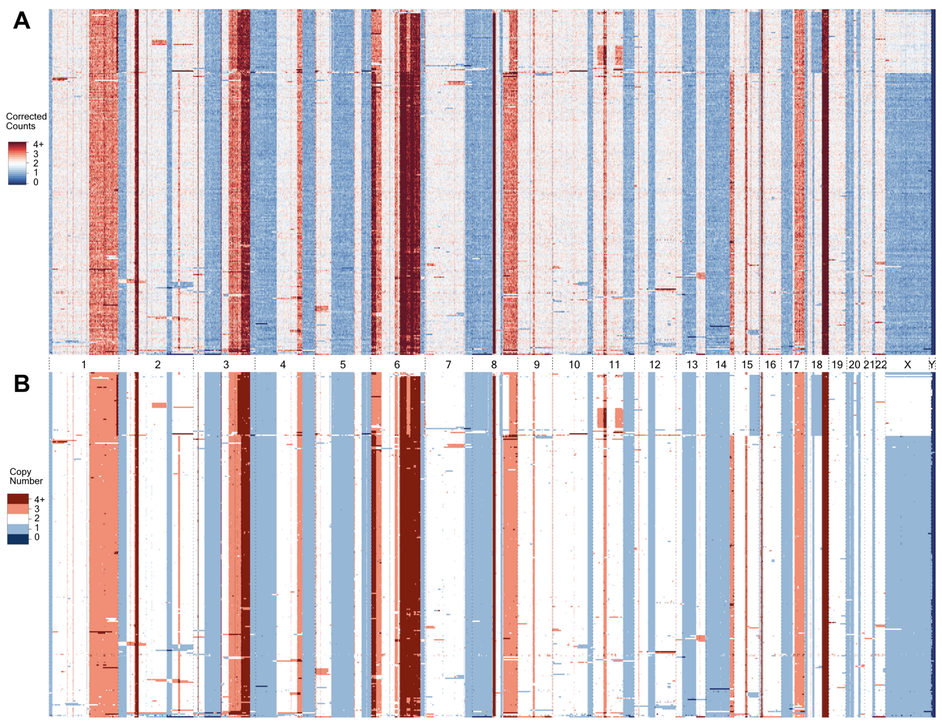

Figure S3 Graphical illustration of CN calling results with CBS+MergeLevels for SA501X3F xenograft breast cancer data set. A The CC heatmap illustrates the biological data with corrected counts in genomic bins, **B** the CN heatmap presents the inferred integer CN for equivalent bins using CBS+MergeLevels. Columns correspond to genomic locations, rows to single cells. The rows in both matrices are in the same order fixed using hierarchical clustering of cells according to their inferred CNs.

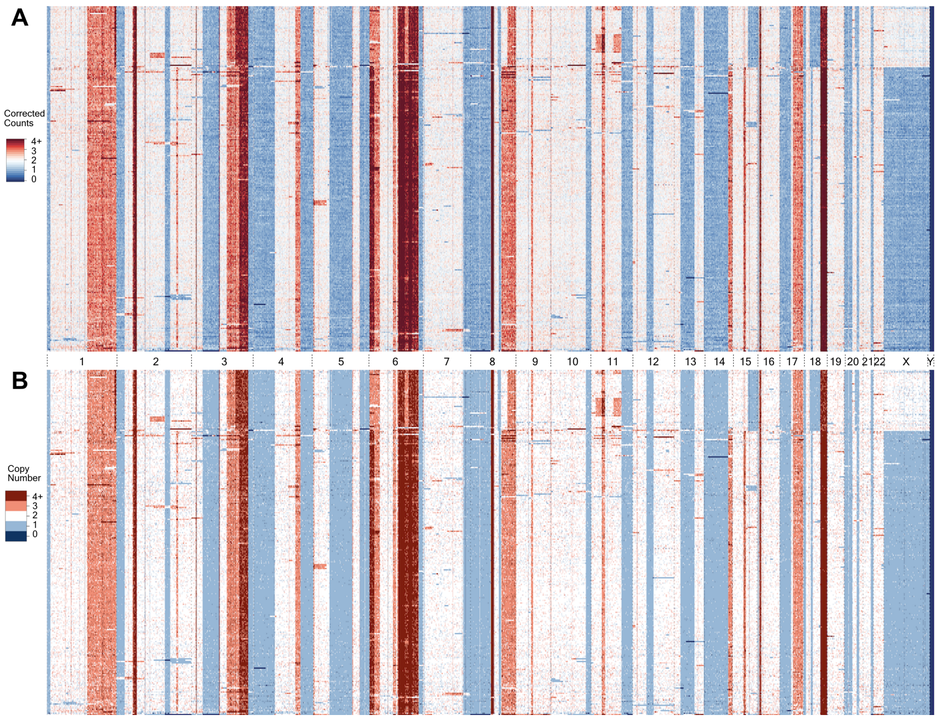

Figure S4 Graphical illustration of CN calling results with rounding for SA501X3F xenograft breast cancer data set. A The CC heatmap illustrates the biological data with corrected counts in genomic bins, **B** the CN heatmap presents the inferred integer CN for equivalent bins using simple rounding. Columns correspond to genomic locations, rows to single cells. The rows in both matrices are in the same order fixed using hierarchical clustering of cells according to their inferred CNs.

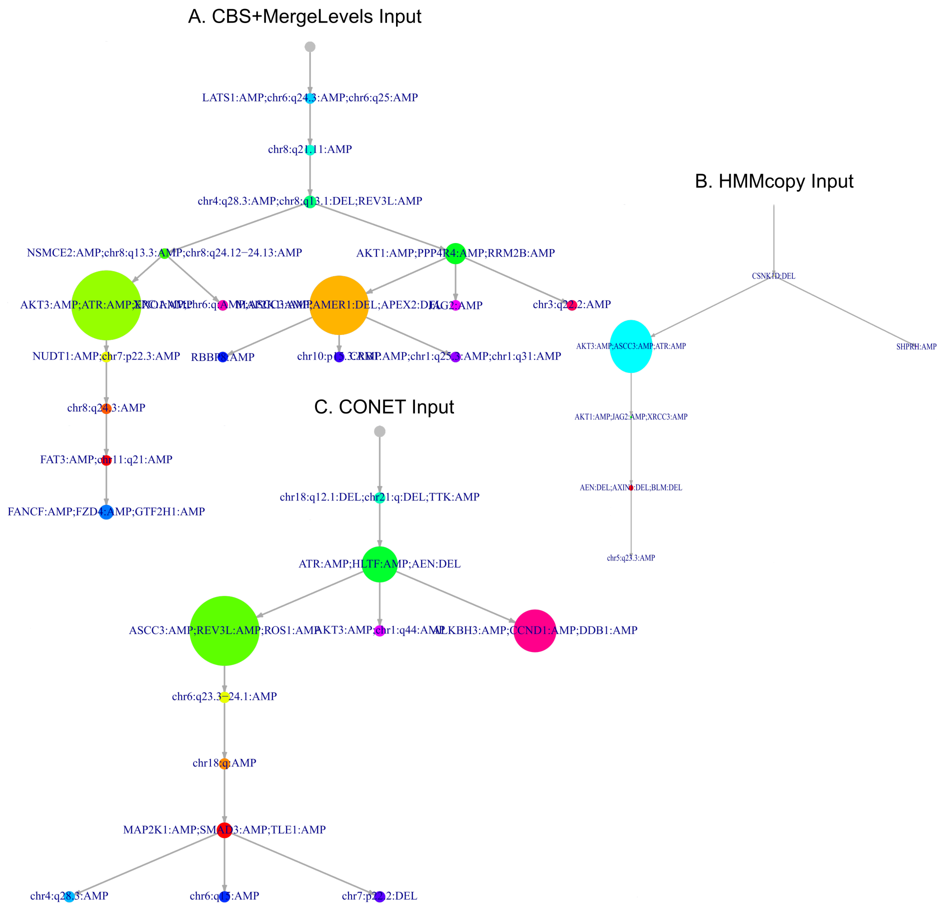

Figure S5 MEDALT trees for CN input from **A** CBS+MergeLevels, **B** HMMCopy, **C** CONET.

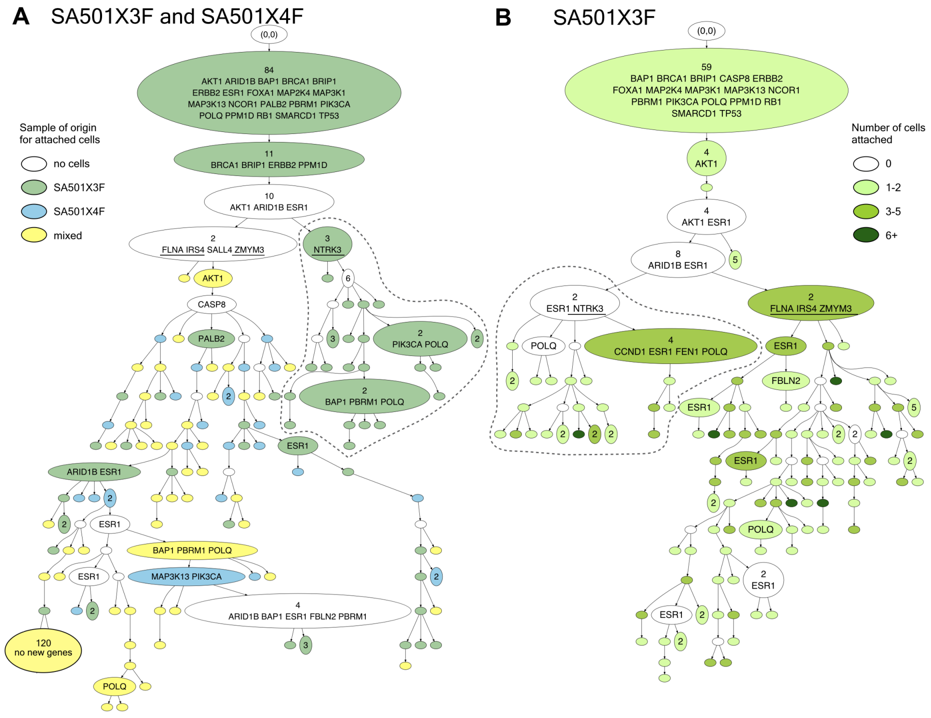

Figure S6 Comparison of CONETs for mixed SA501X3F and SA501X4F vs single SA501X3F xenograft breast cancer samples. A The CONET for SA501X3F and SA501X4F xenograft breast cancer samples. The CONET is drawn in a compacted form, with part of vertices collapsed when it is possible without losing important information i.e. when the collapsed vertex has no cells attached and not more than one child. Specifically, the collapsed vertex is joined with its closest descendant that does not satisfy these criteria (number of events is shown at the beginning of joint vertices). This results in decreasing the tree size from 388 to 240. For clarity, we additionally collapse 120 events into one artificial vertex (left lower corner in the Figure). The names of the breast cancer genes [67] affected by the CN events are printed in alphabetical order in the corresponding vertices, except for the artificial vertex, which only contains events overlapping with cancer genes present in other vertices on the path from this node to the root. The colors illustrate the sample of origin of the cells that are attached to o vertex. Underlined genes correspond to their counterparts in the CONET from B part of the figure. **B** The CONET for SA501X3F xenograft breast cancer sample, reproduced from Figure 4A in the main text.

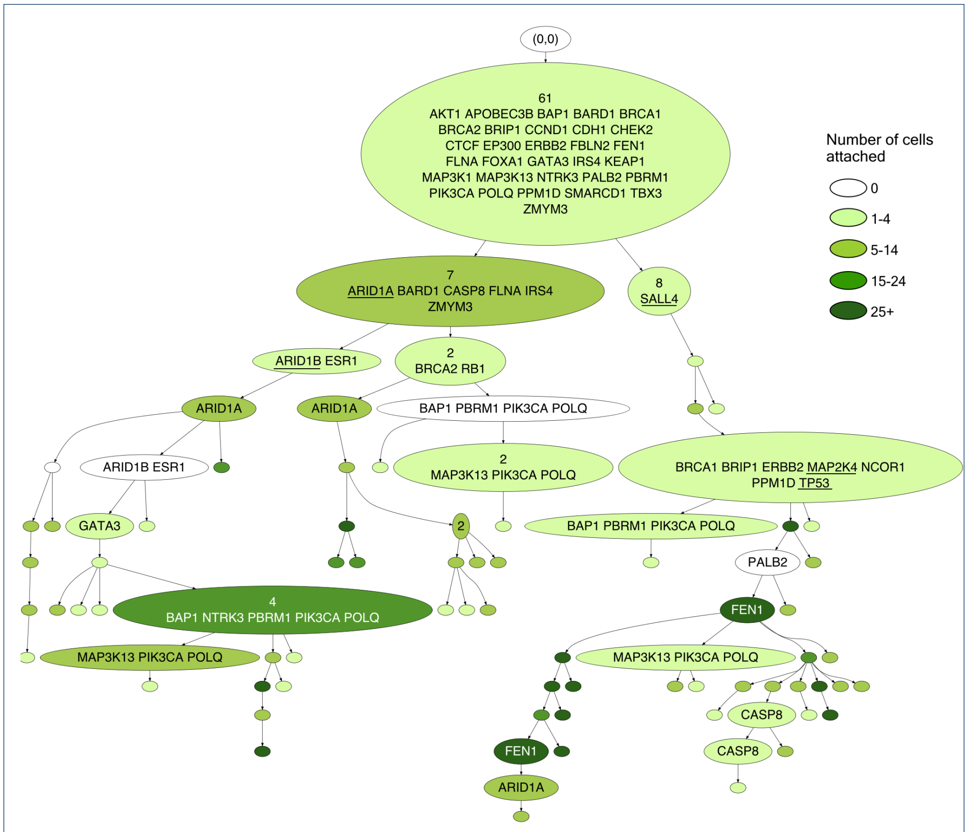

Figure S7 CONET for TN2 invasive ductal carcinoma data set. A The CONET is drawn in a compacted form, with part of vertices collapsed when it is possible without losing important information, i.e. when the collapsed vertex has no cells attached and not more than one child. Specifically, the collapsed vertex is joined with its closest descendant that does not satisfy these criteria (number of events is shown at the beginning of joint vertices). This results in decreasing the tree size from 163 to 84. The number of cells attached to each vertex is illustrated with different colors, where white vertices have no cells attached and the darker green indicates more cells attached. The names of the breast cancer genes [67] affected by the CN events are printed in alphabetical order in the corresponding vertices. Underlined genes are characteristic for the subclones.

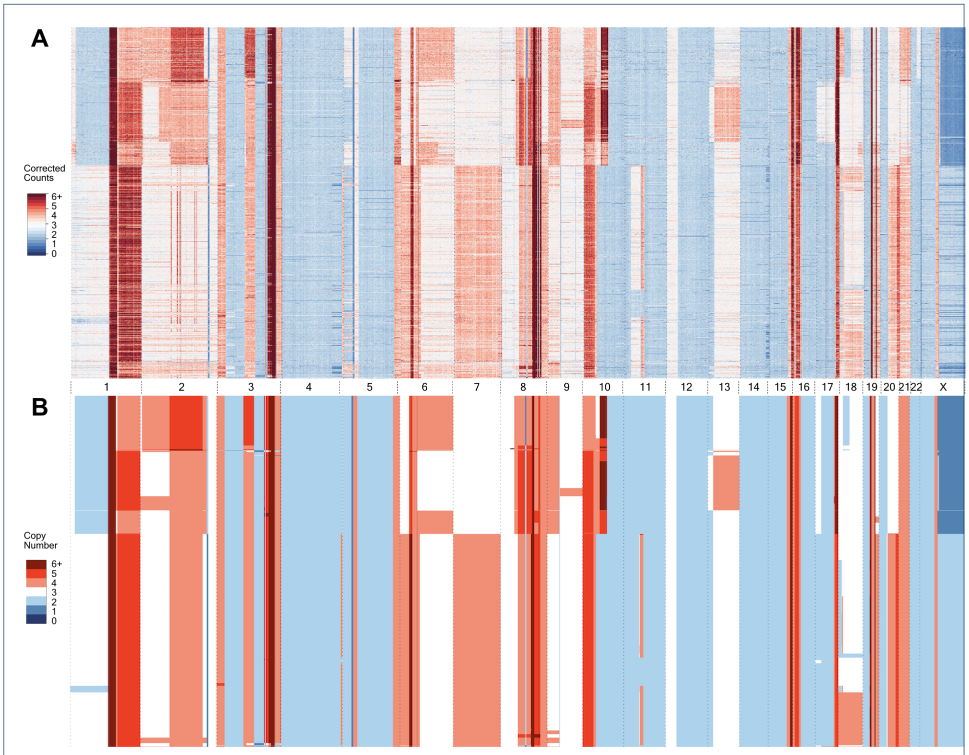

Figure S8 Graphical illustration of CN calling results for for TN2 invasive ductal carcinoma data set. A The CC heatmap illustrates the biological data with corrected counts in genomic bins, **B** the CN heatmap presents the inferred integer CN for equivalent bins. Columns correspond to genomic locations, rows to single cells. The rows in both matrices are in the same order fixed using hierarchical clustering of cells according to their inferred CNs.

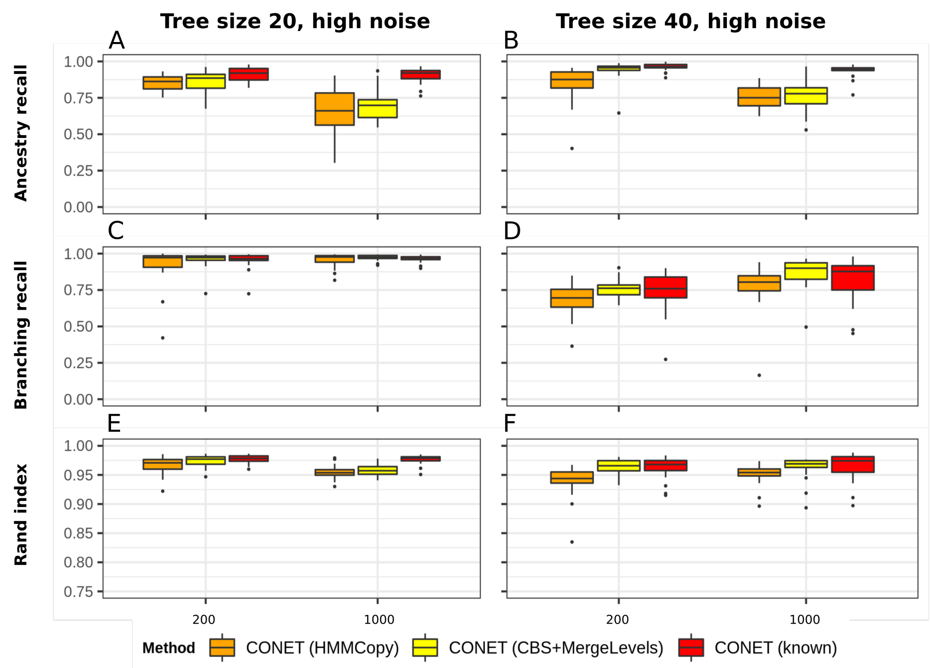

Figure S9 Assessment of CONET recovery of ancestry relations and clustering of cells. A,B Distribution of ancestry recall (y axis) as a function of cell count (x axis) for all three CONET versions (colors), in scenario with real tree of size 20 and high noise (**A**) and in scenario with real tree of size 40 and high noise (**B**). **C, D** Distribution of branching recall (y axis) as a function of cell count (x axis) for all three CONET versions, in scenario with real tree of size 20 and high noise (**C**) and in scenario with real tree of size 40 and high noise (**D**). **E, F** Distribution of rand index (y axis) as a function of cell count (x axis) for all three CONET versions in scenario with real tree of size 20 and high noise (**E**) and in scenario with real tree of size 40 and high noise (**F**). Overall, the results show that even in challenging high-noise settings CONET is able to correctly recover majority of ancestry relations and cell clustering.

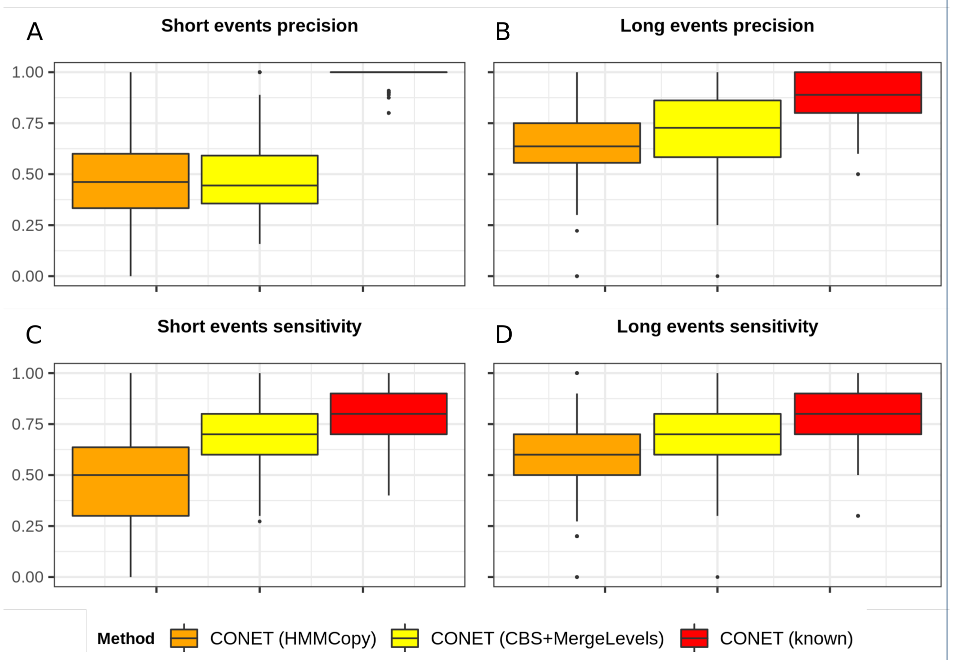

Figure S10 Effectiveness of CONET on short versus long copy number events. A Distribution of event precision (y axis) for short events for all methods (x axis). **B** Distribution of event precision (y axis) for long events for all methods (x axis). **C** Distribution of event sensitivity (y axis) for short events for all methods (x axis). **D** Distribution of event sensitivity (y axis) for long events for all methods (x axis).

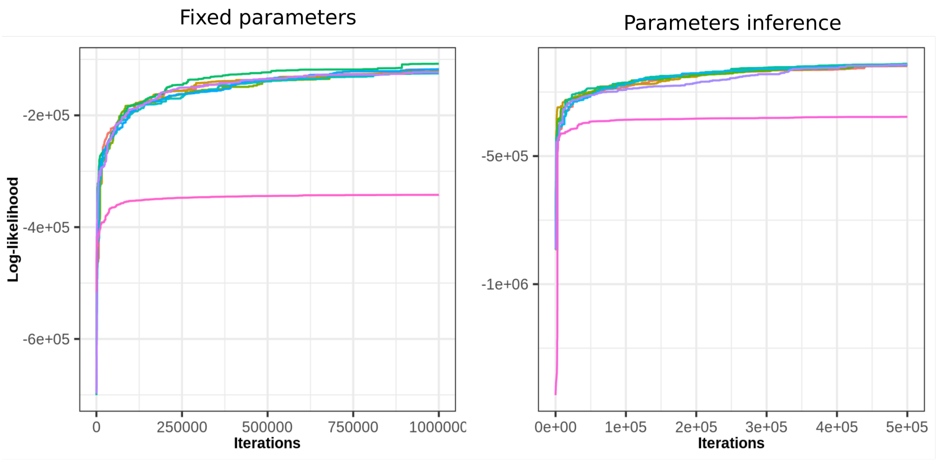

Figure S11 Log-likelihood trace plots for eight runs of CONET on the SA501X3F xenograft breast cancer sample. Right - trace plots for the first chain in the procedure (inference of model's parameters). Left - trace plots for the second chain in the procedure (tree inference). The plots indicate convergence with only one of the eight chains being stuck in local suboptimal maximum.

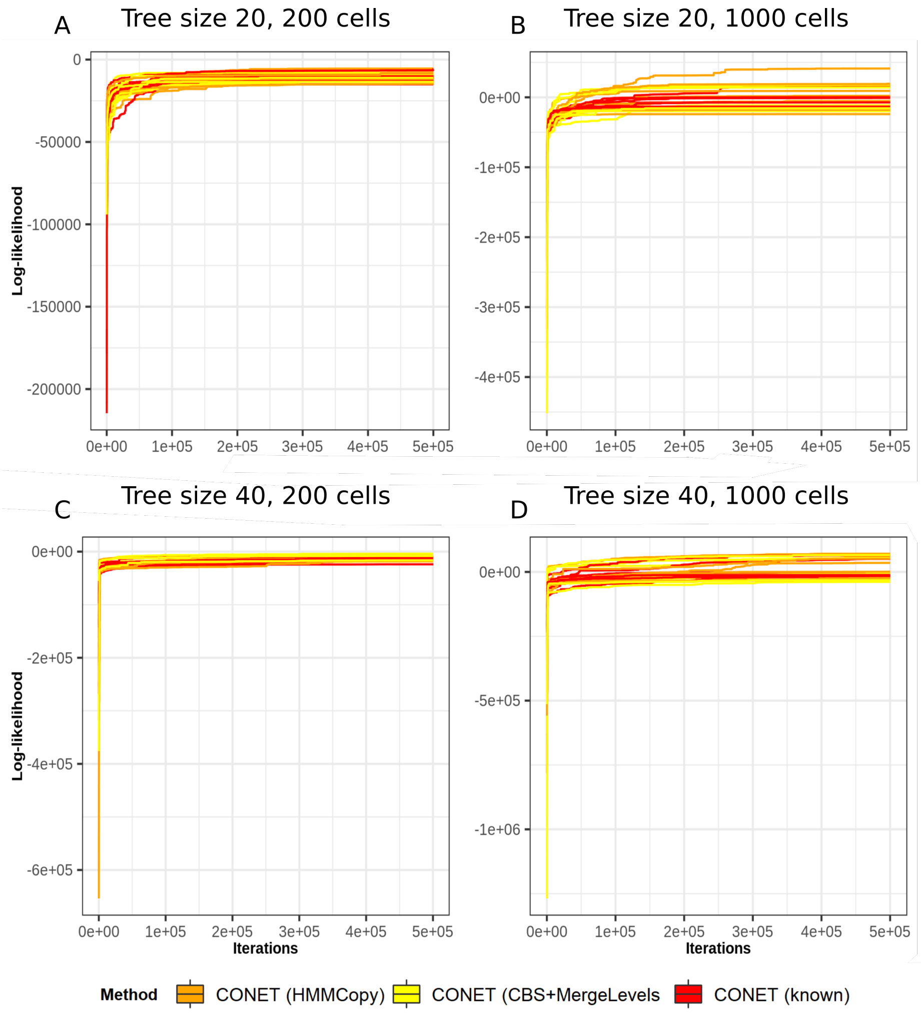

Figure S12 Log-likelihood trace plots of parameters inference chain for different versions of CONET on synthetic data with high noise level. A - trace plots for all three CONET versions (8 runs per version) for model with real tree of size 20 and 200 cells. **B** - trace plots for all three CONET versions (8 runs per version) for model with real tree of size 20 and 1000 cells. **C** - trace plots for all three CONET versions (8 runs per version) for model with real tree of size 40 and 200 cells. **D** - trace plots for all three CONET versions (8 runs per version) for model with real tree of size 40 and 1000 cells.

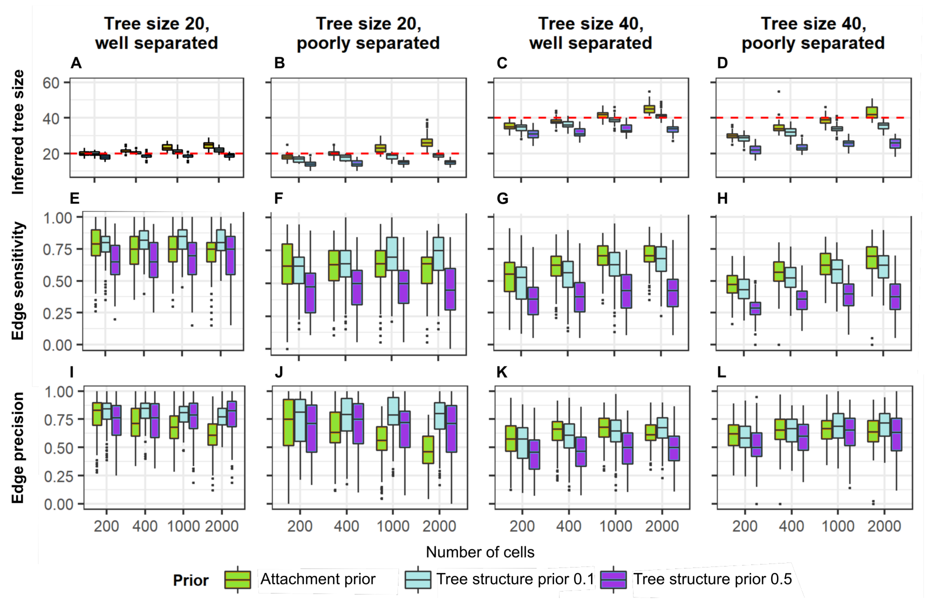

Figure S13 Assessment of the tree structure inference for simulated data tests results. A-D Distribution of inferred tree sizes (y axis) depending on the cell number (x axis) for all simulation scenarios. The horizontal line indicates the size of the true event tree. **E-H** Distribution of edge sensitivity (y axis) depending on the cell number (x axis) for all scenarios. **I-L** Distribution of edge precision (y axis) depending on the cell number (x axis) for all scenarios. The results indicate very high efficiency in detecting real event history. In all figures *Attachment prior* scenario has *Tree structure prior* set to 0. Scenarios with non-zero *Tree structure prior* use uniform attachment probability.

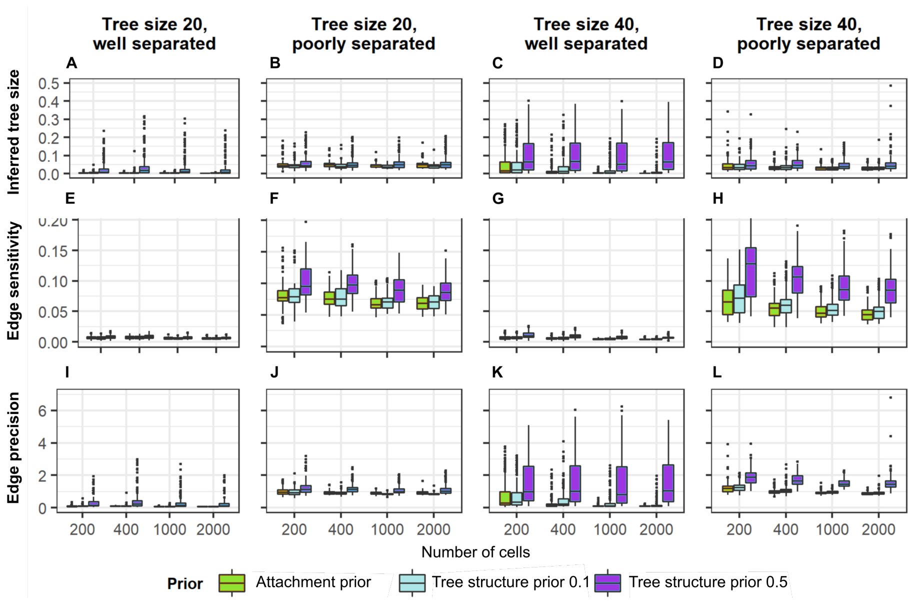

Figure S14 Assessment of breakpoints inference for simulated data tests results. A-D Distribution of false positive rate (y axis) as a function of cell count (x axis) for all simulation scenarios. **E-H** Distribution of false negative rate (y axis) as a function of cell count (x axis) for all scenarios. **I-L** Distribution of symmetric distance score between inferred and real breakpoints (y axis) as a function of cell count (y axis) for all scenarios. Overall results are very good for all scenarios, and good data separability is a determining factor of the quality of the results. In all figures *Attachment prior* scenario has *Tree structure prior* set to 0. Scenarios with non-zero *Tree structure prior* use uniform attachment probability.
