# Supplementary material for: CONET: copy number event tree model of evolutionary tumor history for single-cell data
Source: Genome Biol. 2022 Jun 9;23:128. doi: 10.1186/s13059-022-02693-z (PMC9185904; doi:10.1186/s13059-022-02693-z)
Supplement: Supplementary file 8 — Additional file 8 Review history. [file 13059_2022_2693_MOESM8_ESM.docx]

Review History

**First round of review**

**Reviewer 1**

**Were you able to assess all statistics in the manuscript, including the appropriateness of statistical tests used?**

Yes: I believe that I am competent to assess the statistical content of the manuscript and had no concerns with it, aside from a couple of questions about model convergence and the use of a prior to control model complexity that were raised in my comments to the authors.

**Were you able to directly test the methods?**

No.

**Comments to author:**

This paper describes a new method for inferring evolutionary histories of tumors in terms of copy number variation derived from single-cell data. The problem of copy number evolution in cancers is extremely important in understanding their etiology and mechanism, but it is a challenging problem for computational methods and has historically been relatively neglected by the literature compared to mutation by single nucleotide variations. While there is a long history of copy number phylogeny methods, which the paper does a reasonable job of surveying, there is a need for better methods particularly for the emerging single-cell directions. The present work offers some interesting new ideas, notably combining two sources of sequence evidence --- per-bin and per-breakpoint data --- and integrating copy number and possibly breakpoint inference into phylogenetic tree building. The broad ideas behind the method are sensible and the Bayesian model and the MCMC algorithm to solve for it are well engineered and implemented. The paper does a generally good job of comparing to quite a few related tools in the literature. It provides good empirical evidence for improvement over the prior art by a variety of reasonable metrics based on simulated data. It also provides a reasonable demonstration of effectiveness on a real xenograft data set. It is overall a well written and technically strong study on which I have little to suggest with respect to problems with the methodology. There are, however, some points of the method description that prompt some questions for clarification and some areas in which the empirical validation could be more compelling. I describe a few questions and concerns below:

1. While the model is generally well explained and justified, it does have several user-defined parameters, notably the regularization parameters. I believe more detail is needed on how these parameters were set and incorporated into the validation to demonstrate clearly that the method has not been unfairly tuned to the tests. How were these parameters set in practice for the validation studies? Were they set in a consistent way across tests? And can you provide some evidence that the model is not highly sensitive to these parameters or that a standard parameter set (or standard procedure for setting them) generalizes to independent data sets?

2. It would also be useful to see some evidence about convergence of the methods on the test data. While the results look good and the methods show excellent design for efficient convergence, this is a complicated model where one would expect a high risk of methods failing to converge or getting stuck in local optima.

3. The method is generally well evaluated in terms of simulation data, exploring the relevance of several suitable parameters, appropriate measures of quality, and comparison to related methods. However, I was unclear on how well the simulation protocols capture the relevance of different scales of CNAs, which might be expected to be very relevant to the relative effectiveness of the methods. For example, we might expect the method to have different effectiveness on small focal CNAs vs. changes on large scales or on data with relatively homogeneous vs. highly heterogeneous sizes of CNAs.

4. The study of xenograft data provides a necessary demonstration that the method produces reasonable results on a real data set, but it would be helpful to see some more compelling evidence that the results are correct and lead to some novel insight lacking from prior studies. This is understandably difficult given that there is no certain ground truth and the paper makes some argument based on more reproducibility of calls between cells. While there is a logic to this in arguing that CONET is better at finding phylogenetically informative markers, it is still unclear if the CONET results are actually better given that we might expect a lot of low-reproducibility variations in a tumor with a highly unstable genome. The source paper for the data set [31] provided several other complementary analyses on these tumors as part of their own validation that might be used to provide at least indirect validation of the results here. [31] also had additional data sets profiling different passages of the xenograft model, which might also be used to provide an independent validation of some aspects of the inferred tree. And the same data set has been used for validation of quite a few other tools beyond those directly tested here, at least providing a basis for establishing some consensus against which to compare.

I also note a few minor points:

5. p. 6, line 62: ``low false rate'' seems like it should be either ``low false positive rate'' or ``low false negative rate''

6. p. 8, line 11: ``performs excellent'' should be ``performs excellently''

7. The method caps inferred copy numbers at 10, but one would expect that some focal amplifications, such as the recurrent ESR1 amplification seen in the xenograft case, might have much higher copy number. Is there a simple argument for why you would expect the method to handle such cases reasonably?

8. the model prior term P_{ds} seems intuitively reasonable, but why favor it over a more standard prior for model complexity, such as Bayesian Information Criterion (BIC) or Aikike Information Criterion (AIC)?

**Reviewer 2**

**Were you able to assess all statistics in the manuscript, including the appropriateness of statistical tests used?**

Yes: Test were appropriate.

**Were you able to directly test the methods?**

No.

**Comments to author:**

The manuscript "CONET: Copy number event tree model of evolutionary tumor history for single-cell Data" is introducing a new computational method for jointly calling copy numbers in single-cell DNA sequencing data and inferring the related evolutionary history. The proposed approach is based on very promising intuitions. Moreover, the problem is very interesting and motivated by cutting edge sequencing technologies that will likely represent the future of cancer sequencing studies. However, the key features of the proposed approach are not particularly novel and their impact on the related biological applications have not been clearly demonstrated on either simulated or cancer sequencing data. Importantly, the proposed approach also relies on unrealistic assumptions of cancer evolution that could substantially affect the potential impact of the proposed method. Therefore, the proposed approach in this study does not appear to have the potential for significant impact on the biological application, but rather it represents a more technical advancement.

MAJOR COMMENTS

1. Novelty: The new proposed CONET algorithm is based on two key features, but none of these is fundamentally novel and they do not properly reflect the proposed contributions.

---a. The first key innovation of CONET is a proposed approach that jointly performs copy number calling and infers the related evolutionary history of single cells. While this approach is particularly interesting and promising, the same joint approach has been previously proposed for the same application and data by Kuipers et al. (2020) [1] with SCICoNE. Importantly, it is also not clear what are the key and relevant differences between CONET and SCICoNE, especially it is not clear whether the differences are substantially technical or there is any more fundamental difference.

---b.The second key innovation of CONET is the proposed use of both per-bin and per-breakpoint data, suggesting that these are two distinct signals obtained from sequencing data and their joint analysis provides more power to the inference. However, this claim does not seem accurate as the so called "per-bin" and "per-breakpoint" data are simply different manipulations of the same sequencing data, that are read counts. In fact, per-breakpoint data are simply computed as absolute differences between adjacent per-bin data. While per-breakpoint data are accounting for the genomic location of related bins in addition to per-bin data, the procedure of deciding switch-points based on the read counts of neighboring genomic bins is adopted by nearly all existing algorithms for single-cell copy number calling, such as the HMM algorithm adopted by HMMcopy [2], the CBS algorithm adopted by Ginkgo [3], or the HMM algorithm adopted by Aneufinder [4]. Moreover, approaches to identify breakpoints based on mixed distributions of bins with different copy numbers have also been proposed by very recent single-cell methods, like scope [5]. Lastly, previous bulk-sequencing studies, especially ReMixT [6], have demonstrated that the orthogonal and independent signal from breakpoints inferred through SV analysis can be indeed integrated into copy number analysis and provide more power. However, SVs breakpoints are in fact an orthogonal signal, in contrast to the proposed approach in this study.

2. Benchmark: the proposed benchmark on simulations does not convincingly demonstrate the performance of CONET and its advancements due to three important observations and limitations.

---a. It is unclear whether simulations are realistic, especially because simulations have not been generated based on proper models of sequencing data. Importantly, it is particularly surprising and concerning that there is no mention of either sequencing coverage or total number of reads in the simulations. Instead, simulations have been generated using arbitrary models of the distributions of corrected read counts. Therefore, it is unclear whether the proposed simulations accurately reflect the properties of any actual single cell sequencing dataset, and the performance of existing methods cannot be properly interpreted. This is especially important as different single cell DNA-sequencing technologies have very different features in terms of sequencing coverage and related properties, which fundamentally impact the variances observed in the corrected read counts.

---b. The simulations do not clearly demonstrate that the joint proposed approach of CONET allows an improvement of copy number calls, since one of the benchmarked copy number calling methods (CBS+Mergelevels) consistently and clearly outperforms CONET on at least two metrics. While CONET with known breakpoints has better performance in some instances, this is not relevant as breakpoints are not known in actual applications.

---c. The proposed set of methods for the benchmark does not properly reflect current state of the art for the related field. In fact, some of the most established and important existing copy number calling methods are missing and should be included in the benchmark, especially Ginkgo [3], Aneufinder [4], and possibly some of the most recent methods and advancement like SCOPE [5]. Including some of these methods is critical to convincingly demonstrate that the joint approach proposed by CONET really has the potential to also improve copy number calling.

3. Impact: the proposed model underlying CONET is based on two unrealistic assumptions on cancer biology that would substantially impact its potential impact.

---a. No cell ploidy estimation is performed in CONET, but rather cells are always assumed to have a based ploidy of two. However, aneuploidies and whole genome doublings are very frequent in cancer, and therefore ploidy estimation is one of the most important and challenging steps of copy number calling methods. Therefore, the underlying diploid assumption is not realistic and particularly concerning, and the lack of genome ploidy estimation substantially impacts the applicability of the proposed method.

---b. The underlying model of CONET is assuming that each copy number event occurs only once across different cells. However, the assumption does not seem to be particularly realistic especially when considering recurrent altered regions, like chromosomal arms and whole chromosomes; in fact, it is frequent in cancer to observe such subclonal events affecting the same chromosome multiple times in different cells within the same tumor.

---c. The impact of CONET in terms of biological applications has not been convincingly demonstrated. In fact, it is not clear if any of the proposed features of CONET are enabling the authors to identify new biology or new information from the previous data. Also, the results on the SA501X3F xenograft breast cancer sample do not seem to substantially differ from the input data and it would be critically important to compare it directly with the results that can be obtained using other existing methods, or even naïve approaches like simple rounding; specifically, can the authors show what the copy numbers inferred by other methods look like in a similar figure? Moreover, CONET removed some events that seemed to be clearly present in small subgroups of cells (especially top part of the heatmap) while keeping other events present in similar numbers of cells; what are the differences between these events and was the choice strongly supported by the underlying data?

MINOR COMMENTS.

1. The text mentions that corrections for GC and mappability biases are applied, but no detail is provided anywhere in the text. As there is not a unified correction for these biases, it is fundamental to report the related details.

2. A very important test is missing in the simulated benchmark: how does CONET's result compare to a stepwise approach in which copy numbers are first inferred (for example using CBS+Mergelevels) and then a tree is reconstructed based on the inferred copy numbers, for example using MEDICC [8] or similar methods?

3. In addition to SCICoNE, it would be important to discuss the differences between CONET and other recent copy number evolutionary models based on breakpoints, as the one proposed by Dorri et al (2020) [7]?

4. An important question is the scalability of the proposed method, especially as it relies on a MCMC algorithm. Therefore, including simulations to explore this for varying numbers of cells would be very important, also considering the high number of cells in the most recent single cell DNA sequencing datasets (>2000-5000).

5. Main text is specifying that nodes of the trees correspond to events, but it does not look like to be the case from the reported figures, as every node seems to be defined by the combination of all the acquired events.

6. The exact definitions of infinite and finite site assumptions should be clearly stated to make the reading accessible to a broad audience.

7. The colors of the same methods in the different result figures are inconsistent.

8. Since HMMcopy is also inferring the ploidy of the different cells, it would be fairer in the simulations to fix the ploidy estimate in HMMcopy to the same value used by CONET.

9. The exact motivation and need of the "count discrepancy penalty" is very unclear to the reviewer, despite its reported importance. A clarification and better explanations of this regularization would be particularly important.

10. In the results for the SA501X3F xenograft breast cancer sample, CONET infers a tree in which nearly all the nodes only contain <5 cells. This result raises some concerns about the accuracy of the results: how likely is that there have been some many clonal expansions in the tumor, and we were so lucky to sample so many of them with such low numbers of cells?

11. In the real data results, one of the comparisons between the results of different methods is based on the differences from the corrected read counts and the inferred copy numbers (absolute differences). However, different methods have likely applied different corrections and normalizations, so it is not surprising that other methods have higher differences, as they have minimized different quantities. Therefore, this comparison is not accurate.

REFERENCES:

[1] Kuipers, J., Tuncel, M. A., Ferreira, P., Jahn, K., & Beerenwinkel, N. (2020). Single-cell copy number calling and event history reconstruction. bioRxiv (https://doi.org/10.1101/2020.04.28.065755).

[2] Laks, E., McPherson, A., Zahn, H., Lai, D., Steif, A., Brimhall, J., ... & Shah, S. P. (2019). Clonal decomposition and DNA replication states defined by scaled single-cell genome sequencing. Cell, 179(5), 1207-1221.

[3] Garvin, T., Aboukhalil, R., Kendall, J., Baslan, T., Atwal, G. S., Hicks, J., ... & Schatz, M. C. (2015). Interactive analysis and assessment of single-cell copy-number variations. Nature methods, 12(11), 1058-1060.

[4] Bakker, B., Taudt, A., Belderbos, M. E., Porubsky, D., Spierings, D. C., de Jong, T. V., ... & Foijer, F. (2016). Single-cell sequencing reveals karyotype heterogeneity in murine and human malignancies. Genome biology, 17(1), 1-15.

[5] Wang, R., Lin, D. Y., & Jiang, Y. (2020). SCOPE: a normalization and copy-number estimation method for single-cell DNA sequencing. Cell systems, 10(5), 445-452.

[6] McPherson, A. W., Roth, A., Ha, G., Chauve, C., Steif, A., de Souza, C. P., ... & Shah, S. P. (2017). ReMixT: clone-specific genomic structure estimation in cancer. Genome biology, 18(1), 1-14.

[7] Dorri, F., Salehi, S., Chern, K., Funnell, T., Williams, M., Lai, D., Andronescu, M., Campbell, K.R., McPherson, A., Aparicio, S., Roth, A., Shah, S., Bouchard-Cˆot´e, A.: Efficient bayesian inference of phylogenetic trees from large scale, low-depth genome-wide single-cell data. bioRxiv (2020)

[8] Schwarz, R. F., Trinh, A., Sipos, B., Brenton, J. D., Goldman, N., & Markowetz, F. (2014). Phylogenetic quantification of intra-tumour heterogeneity. PLoS computational biology, 10(4), e1003535.

**Reviewer 3**

**Were you able to assess all statistics in the manuscript, including the appropriateness of statistical tests used?**

No.

**Were you able to directly test the methods?**

No.

**Comments to author:**

This paper introduces CONET, a method for tree inference and copy-number calling from single-cell DNA-seq data of tumors. The paper includes a comparison against HMMcopy, CBS+MergeLevels and SCICoNE. Moreover, the paper shows the application of CONET to a DLP+ xenograft sample. I have the following comments.

1. Input to CONET does not resemble real data

My first major concern is that CONET in its present form is not an independent method. Ideally, CONET would start from BAM files and produce the final output (tree, cell-to-clone clustering and CN matrix). However, the input that CONET requires are corrected read counts per bin, as well as a set of candidate breakpoints. For the real data set analyzed in this paper, the former are obtained from the DLP+ paper and the latter are obtained by running HMMcopy (segmentation method). This is not good -- to facilitate use of the method on other datasets, I suggest the authors include pre-processing scripts to obtain the CONET input.

Moreover, the "corrected read counts" seem to essentially correspond to copy numbers. For example, Eq. (10) describes that inferred copy-number of a clone (i.e. group of cells) is simply the average input "corrected read count" of that bin among cells assigned to the clone. It is not clear to me how one would obtain such a quantity. Moreover, if it is indeed straightforward to infer "corrected read counts" from actual read counts then a simple baseline method would be to simply cluster the cells according to this quantity without using a tree.

- Line 28, p. 15: "corrected for GC content and other potential biases" -- please expand on this, discuss what biases might expect and how one can correct for this.

2. CONET model

- I had a hard time understanding the CONET model. In particular, the manuscript uses "CV event" throughout the manuscript only formally introducing it for the first time in methods. Please provide a definition as soon as you use this term for the time in the Introduction.

- I suggest improving the presentation by including a graphical model (plate diagram). Right now, the method seems rather ad hoc with various penalties.

- I am worried that the introduced tree model may lead to unrealistic inferences, as it does not incorporate practical evolutionary constraints regarding copy number. For example, what if you have a complete loss of a segment? The CONET model may amplify this segment later, something that cannot happen in practice.

- Good to provide an example figure, demonstrating I_bp, I_0, CN events, how one obtains CNs, etc.

- Considering recent work (CHISEL), I would appreciate a discussion of allele-specific copy numbers. Can the CONET model be extended to support this?

- Why normal distributions? Does this match real data?

- How to go from breakpoints to bins? Do you discard bins that fall within but do not overlap with two breakpoints? If not, do you merge bins between breakpoints into segments -- and if so, how do you account for differences in read count due to varying segment lengths?

3. Experimental evaluation

- Simulations are generated from same model used in the inference by CONET. In particular, the issue here is the use of "corrected read counts" -- per Major Comment 1, these are not read counts but rather reflect an estimate of integer copy number. It would be good if the simulated data closely resembled real data, to properly assess the performance of CONET and its generalizability to other datasets. I suggest using a simulator of read counts such as the one introduced in:

Mallory XF, Edrisi M, Navin N, Nakhleh L. Assessing the performance of methods for copy number aberration detection from single-cell DNA sequencing data. Ioshikhes I, ed. PLoS Comput Biol. 2020;16(7):e1008012. doi:10.1371/journal.pcbi.1008012

- Performance metrics should include assessment of cell clustering (such as Rand index, V-measure) as well as ordering of cells (ancestral pair recall, incomparable pair recall have been used in the literature for this.)

- How much is the tree helping? Following up on my first comment, the evaluation should include a comparison against a version of CONET without the tree, or a baseline method where one directly clusters "corrected read counts".

- To show generalizability of the method, I suggest including additional real data experiments beyond the one DLP+ sample. For example, consider including 10X Genomics, ACT and standard DOP-PCR data.

4. Code and usability

README.md on GitHub page does not include example usage, nor a description of the input and output formats, or instruction on how to run the executables. This makes using the method extremely hard.

5. Discussion of previous work in Introduction

Introduction does not adequately discuss previous work, including MEDALT, copy-number tree problems, CHISEL, copy-number calling in bulk DNA-seq data, etc.

Minor comments:

- Page 3, line 54: "defined by a tree" => "defined by a ROOTED tree".

- Page 5, line 23 -- these are running times on what system?

- Eq. (1) -- very confusing condition (i - 1 >= 1). Why not write i >= 2.

**Authors Response**

**Point-by-point responses to the reviewers’ comments:**

*Response: We are grateful to all three Reviewers for their detailed, thoughtful and insightful reviews. In this revision, motivated by the Reviewers’ comments, we have improved three main Figures, two of which include new results, extended one Table in the main text with new results, added 11 new Supplementary Figures and two Supplementary Tables with new results, and improved two Supplementary Figures. We believe that the work invested in this revision has greatly improved both the method and the manuscript.*

Reviewer #1:

This paper describes a new method for inferring evolutionary histories of tumors in terms of copy number variation derived from single-cell data. The problem of copy number evolution in cancers is extremely important in understanding their etiology and mechanism, but it is a challenging problem for computational methods and has historically been relatively neglected by the literature compared to mutation by single nucleotide variations. While there is a long history of copy number phylogeny methods, which the paper does a reasonable job of surveying, there is a need for better methods particularly for the emerging single-cell directions. The present work offers some interesting new ideas, notably combining two sources of sequence evidence --- per-bin and per-breakpoint data --- and integrating copy number and possibly breakpoint inference into phylogenetic tree building. The broad ideas behind the method are sensible and the Bayesian model and the MCMC algorithm to solve for it are well engineered and implemented. The paper does a generally good job of comparing to quite a few related tools in the literature. It provides good empirical evidence for improvement over the prior art by a variety of reasonable metrics based on simulated data. It also provides a reasonable demonstration of effectiveness on a real xenograft data set. It is overall a well written and technically strong study on which I have little to suggest with respect to problems with the methodology. There are, however, some points of the method description that prompt some questions for clarification and some areas in which the empirical validation could be more compelling. I describe a few questions and concerns below:

1. While the model is generally well explained and justified, it does have several user-defined parameters, notably the regularization parameters. I believe more detail is needed on how these parameters were set and incorporated into the validation to demonstrate clearly that the method has not been unfairly tuned to the tests. How were these parameters set in practice for the validation studies? Were they set in a consistent way across tests?

*Answer: Thank you for pointing this out. To improve the presentation and analysis of the regularization parameters we have implemented several changes to the manuscript.*

*First, to make the reader more familiar with the topic of the regularization parameters, we extended their description in the main text in Section Model overview on page 5.*

*Second, we now explicitly mention that for all CONET runs on simulated data, which were presented in the main text in Section Performance of CONET in comparison to other methods on simulated data on page 5, the parameters were fixed to the same values.*

*Third, all used parameter settings for CONET in all experiments, in application to both simulated and real data are now listed in a new Additional File 1: Section S1 CONET regularization parameter values used in the presented results on page 3.*

— And can you provide some evidence that the model is not highly sensitive to these parameters or that a standard parameter set (or standard procedure for setting them) generalizes to independent data sets?

*Answer: Thank you for this comment. We have comprehensively analyzed the sensitivity to the regularization parameters in two ways.*

*First, we have evaluated the effect of different settings of the tree structure prior and of using the attachment prior (see Additional File 1: Section S5 Performance of CONET using different priors on per-breakpoint data simulated from the model on pages 5-8 and Figures S14 and S14) on simulated per-breakpoint data.*

*Second, we have analyzed the impact of the count discrepancy penalty on the real data from the SA501X3F xenograft breast cancer sample (see Additional File 1: Section S6 Evaluation of the count discrepancy penalty for scDNA-seq data sample on pages 8-11 ). Both these analyses were not highlighted well enough in the previous version of the text.*

*To improve the presentation of the regularization parameters in the main text, we added a new Section Assessment of the impact of the regularization parameters and of data separability on CONET performance on page 17, providing a better intuition about the impact of the regularization parameters on the inference.*

*In addition, following the Reviewer’s suggestion, we now added to the Additional File 1: Section S7 A recommended procedure for setting CONET parameters on pages 11-12, addressing this point.*

*Finally, to better assist the users in choosing the parameter settings, we have also extended the README of the CONET project on github adding a Guide to parameter settings.*

2. It would also be useful to see some evidence about convergence of the methods on the test data. While the results look good and the methods show excellent design for efficient convergence, this is a complicated model where one would expect a high risk of methods failing to converge or getting stuck in local optima.

*Answer: Thank you for this suggestion. We inspected model convergence both on simulated and real data. A detailed description of this new analysis is featured in the main text in Section CONET convergence analysis on page 16. The likelihood trace plots for multiple runs, presented in Additional File 1: Figures S11 and S12, show high agreement between different runs and indicate likelihood convergence both for real and simulated data. Summary of inferred model parameter values for different runs is presented in Additional File 1: Table S2. Despite high noise in the analyzed simulated data, inferred parameters for CONET models exhibit excellent reproducibility, with very small differences among parameters inferred from different runs, varying tree size and numbers of cells.*

3. The method is generally well evaluated in terms of simulation data, exploring the relevance of several suitable parameters, appropriate measures of quality, and comparison to related methods. However, I was unclear on how well the simulation protocols capture the relevance of different scales of CNAs, which might be expected to be very relevant to the relative effectiveness of the methods. For example, we might expect the method to have different effectiveness on small focal CNAs vs. changes on large scales or on data with relatively homogeneous vs. highly heterogeneous sizes of CNAs.

*Answer: As suggested, we have comprehensively analyzed the effectiveness of CONET on short versus long copy number events. To this end, we have compared event precision and sensitivity on these two types of events, for CONET with three different sets of candidate breakpoints loci: i) true breakpoint sites, ii) loci identified as breakpoints by HMMCopy and iii) loci identified as breakpoints by CBS followed by MergeLevels. The comparison of results revealed that the event detection performance is similar for long and short events. Although there are some differences between long and short events, there is no systematic trend showing that the performance of CONET depends on the event length.*

*The new analysis is now described in Additional File 1: Section S4 Comparison of event discovery for short and long events on page 4. The results are presented in Additional File 1: Figure S10. We refer to and conclude from these analyses in the main text in Section Performance of CONET in three additional challenging tasks on page 16.*

4. The study of xenograft data provides a necessary demonstration that the method produces reasonable results on a real data set, but it would be helpful to see some more compelling evidence that the results are correct and lead to some novel insight lacking from prior studies. This is understandably difficult given that there is no certain ground truth and the paper makes some argument based on more reproducibility of calls between cells. While there is a logic to this in arguing that CONET is better at finding phylogenetically informative markers, it is still unclear if the CONET results are actually better given that we might expect a lot of low-reproducibility variations in a tumor with a highly unstable genome. The source paper for the data set [31] provided several other complementary analyses on these tumors as part of their own validation that might be used to provide at least indirect validation of the results here. [31] also had additional data sets profiling different passages of the xenograft model, which might also be used to provide an independent validation of some aspects of the inferred tree. And the same data set has been used for validation of quite a few other tools beyond those directly tested here, at least providing a basis for establishing some consensus against which to compare.

*Answer: Thank you for this insightful suggestion. In the revised manuscript, we show the applicability of CONET and its ability to deliver correct and novel biological insights on two additional real datasets:*

*an additional passage xenograft breast cancer sample SA501X4F sequenced using DLP, as suggested by Reviewer 1 (discussed in the main text in Section Additional validation of CONET using passage SA501X4F xenograft breast cancer sample on page 14, visualized in Additional File 1: Figure S6),*

*another, independent breast cancer sample TN2 from a different study, sequenced using ACT (discussed in the main text in Section Application to scDNA-seq data of TN2 invasive ductal carcinoma sample on page 15, visualized in Additional File 1: Figures S7 and S8).*

*In the case of the additional analysis on the xenograft samples, the tree inferred from the mixed sample (original plus the passage sample) shows that the additional cells attach only in a subtree of the tree inferred from the original xenograft sample.*

I also note a few minor points:

5. p. 6, line 62: ``low false rate'' seems like it should be either ``low false positive rate'' or ``low false negative rate''

*Answer: “Lower false positive rate” - changed in the manuscript.*

6. p. 8, line 11: ``performs excellent'' should be ``performs excellently''

*Answer: Changed in the manuscript as suggested.*

7. The method caps inferred copy numbers at 10, but one would expect that some focal amplifications, such as the recurrent ESR1 amplification seen in the xenograft case, might have much higher copy number. Is there a simple argument for why you would expect the method to handle such cases reasonably?

*Answer: The capping was set for the purpose of graphical illustration of the final CN calling results. It is now implemented as a user-defined parameter (changed in the main text Section CN calling on page 27). The parameter does not influence the tree inference nor the CN calling procedure model.*

8. the model prior term P_{ds} seems intuitively reasonable, but why favor it over a more standard prior for model complexity, such as Bayesian Information Criterion (BIC) or Aikike Information Criterion (AIC)?

*Answer: Indeed, in a more standard case, BIC or AIC would be used. BIC or AIC penalizes the number of parameters and data points, but does not penalize the tree size, which was the main goal of the P_{ds} prior.*

Reviewer #2:

The manuscript "CONET: Copy number event tree model of evolutionary tumor history for single-cell Data" is introducing a new computational method for jointly calling copy numbers in single-cell DNA sequencing data and inferring the related evolutionary history. The proposed approach is based on very promising intuitions. Moreover, the problem is very interesting and motivated by cutting edge sequencing technologies that will likely represent the future of cancer sequencing studies. However, the key features of the proposed approach are not particularly novel and their impact on the related biological applications have not been clearly demonstrated on either simulated or cancer sequencing data. Importantly, the proposed approach also relies on unrealistic assumptions of cancer evolution that could substantially affect the potential impact of the proposed method. Therefore, the proposed approach in this study does not appear to have the potential for significant impact on the biological application, but rather it represents a more technical advancement.

MAJOR COMMENTS

1. Novelty: The new proposed CONET algorithm is based on two key features, but none of these is fundamentally novel and they do not properly reflect the proposed contributions.

---a. The first key innovation of CONET is a proposed approach that jointly performs copy number calling and infers the related evolutionary history of single cells. While this approach is particularly interesting and promising, the same joint approach has been previously proposed for the same application and data by Kuipers et al. (2020) [1] with SCICoNE. Importantly, it is also not clear what are the key and relevant differences between CONET and SCICoNE, especially it is not clear whether the differences are substantially technical or there is any more fundamental difference.

*Answer: Indeed, one of the innovations of our approach is the joint inference of the copy number event tree and copy number calling, and on a general level, SCICoNE has the same feature. Still, there are several reasons why we are confident that CONET should be considered a highly novel approach.*

*There are at least two key methodological differences between CONET and SCICoNE (in addition to several purely technical differences). One difference is that SCICoNE does not model the per-breakpoint data, and computes its likelihood solely based on the per-bin data. In contrast, CONET computes the model likelihood with terms defined on the per-breakpoint data, and not per-bin data. Another is that SCICoNE builds a tree labelled not only by the events defined as breakpoint pairs, but also their copy numbers. This vastly enlarges the space of possible models and makes the model search much more difficult than it is for CONET. At the same time, it is the feature that SCICoNE uses to be able to make copy number calling. We instead maintain a smaller search space, and use a new count discrepancy penalty (computed efficiently based on per-bin data), which essentially is a trick that i) allows us to regularize the model using the per-bin data, and ii) perform copy number calling with much less computational effort put into the MCMC sampling. An extended discussion of the differences between CONET and SCICoNE, and other models was added in the main text in Section Discussion on page 18.*

*On top of that, we would like to point out that Kuipers et al. (2020) is a preprint. To make it clear that it is a preprint, we now explicitly mention it in the main text in Section Background on page 3 when Kuipers et al. (2020) is first mentioned. Apart from CONET and that preprint, there exist no other approach (neither preprint nor published) that can jointly perform copy number calling and infer the related copy number evolutionary history of single cells. In fact, there exist very few methods that can do anything related to the phylogeny of copy number events, as it is a difficult computational problem. This is again emphasizing the novelty of our approach.*

*Finally, we would like to emphasize that CONET results show a clear advantage over SCICoNE’s performance. It is demonstrated both for simulated data (in the main text in Section Performance of CONET in comparison to other methods on simulated data on pages 5-9 and Figures 2 and 3), and for biological data (in the main text in Section Comparison to other methods on pages 12-14).*

---b.The second key innovation of CONET is the proposed use of both per-bin and per-breakpoint data, suggesting that these are two distinct signals obtained from sequencing data and their joint analysis provides more power to the inference. However, this claim does not seem accurate as the so called "per-bin" and "per-breakpoint" data are simply different manipulations of the same sequencing data, that are read counts. In fact, per-breakpoint data are simply computed as absolute differences between adjacent per-bin data.

*Answer: Indeed, the per-bin and per-breakpoint data are computed from the same sequencing observations. They give, however, very different signals to the method. To clarify: the per-breakpoint data is a better indication of the existence of specific breakpoints in specific cells. We thus use the per-breakpoint data to compute the likelihood of our tree with breakpoint pairs as CN events. In contrast, the per-bin data are more indicative of copy number levels, which we do not model directly and do not use to compute the likelihood of the tree, but rather use for regularization (count discrepancy penalty), and for copy number calling.*

— While per-breakpoint data are accounting for the genomic location of related bins in addition to per-bin data, the procedure of deciding switch-points based on the read counts of neighboring genomic bins is adopted by nearly all existing algorithms for single-cell copy number calling, such as the HMM algorithm adopted by HMMcopy [2], the CBS algorithm adopted by Ginkgo [3], or the HMM algorithm adopted by Aneufinder [4]. Moreover, approaches to identify breakpoints based on mixed distributions of bins with different copy numbers have also been proposed by very recent single-cell methods, like scope [5].

*Answer: Clearly, all listed approaches are methods for identification of copy number variants and as such they must identify points where copy numbers change. None of them uses per-breakpoint data as defined for CONET, i.e., the absolute count differences, and none of them uses the same distribution to model the data. Most importantly, none of them performs copy number calling based on an evolutionary model of copy number events. We now introduce these copy number calling methods in the main text in Section Background on page 2. We also discuss the differences between CONET and these copy number calling approaches in the main text in Section Discussion on page 18.*

— Lastly, previous bulk-sequencing studies, especially ReMixT [6], have demonstrated that the orthogonal and independent signal from breakpoints inferred through SV analysis can be indeed integrated into copy number analysis and provide more power. However, SVs breakpoints are in fact an orthogonal signal, in contrast to the proposed approach in this study.

*Answer: Thank you for pointing to ReMixT and SV analysis. As the strength of CONET lies in an integrative approach - combining per-breakpoint and per-bin data, as well as simultaneous tree inference and copy number calling - it could indeed potentially further benefit from incorporating additional signals such as the topology of genomic structural variants. This is now stated in the main text in Section Discussion on page 19.*

2. Benchmark: the proposed benchmark on simulations does not convincingly demonstrate the performance of CONET and its advancements due to three important observations and limitations.

---a. It is unclear whether simulations are realistic, especially because simulations have not been generated based on proper models of sequencing data. Importantly, it is particularly surprising and concerning that there is no mention of either sequencing coverage or total number of reads in the simulations. Instead, simulations have been generated using arbitrary models of the distributions of corrected read counts. Therefore, it is unclear whether the proposed simulations accurately reflect the properties of any actual single cell sequencing dataset, and the performance of existing methods cannot be properly interpreted. This is especially important as different single cell DNA-sequencing technologies have very different features in terms of sequencing coverage and related properties, which fundamentally impact the variances observed in the corrected read counts.

*Answer: Thank you very much for raising this point. Since our model does not take as input raw sequencing data, but data that is pre-processed (normalized and corrected for biases), we naturally also simulate such input data. The used data distributions are realistic, as they were carefully defined based on analysis of real sequencing data. In the revised manuscript, we show that the assumed data distributions fit the real data perfectly, for datasets generated using two different technologies, DLP and ACT (Additional File 1: Figure S1). This is mentioned in the main text in Section Model Overview on page 3.*

---b. The simulations do not clearly demonstrate that the joint proposed approach of CONET allows an improvement of copy number calls, since one of the benchmarked copy number calling methods (CBS+Mergelevels) consistently and clearly outperforms CONET on at least two metrics. While CONET with known breakpoints has better performance in some instances, this is not relevant as breakpoints are not known in actual applications.

*Answer: In this revision, we have made several improvements and additional analyses.*

*We have modified the CN calling procedure. The CN calling is performed in two steps. First, we identify such sets of bins from different cells that underwent the same evolutionary history according to their attachment to the inferred CONET. These are simply such bins that were changed by the same set of CN events on the way down from the root to the nodes to which their cells are attached. Next, bins in each set are assigned an estimated integer copy number that is a rounded median (previously: mean - this point was modified) of the corrected counts across those bins. The change from taking mean to median, made in this revision, is justified by the fact that the median is more robust to outliers. This modification has improved CONET CN calling results (in the main text updated Figure 3 M-P).*

*We have realized that we previously reported MSE and not RMSE, which is now corrected (in the main text updated Figure 3 M-P).*

*Additionally, we now extended the CN calling results analysis for simulated data (in the main text in Section Performance of CN calling on page 9, Figure 3) to include also results for CONET with candidate breakpoint loci defined using CBS+MergeLevels, denoted CONET (CBS+MergeLevels).*

*Finally, we illustrate the problem with noise in the data, by adding a comparison to the naive approach of rounding the per-bin counts to nearest integers (shortly CC-rounding; illustrated in a new figure in Additional File 1: Figure S4). CC-rounding overfits the data and calls false positive breakpoints resulting in a dramatically high false positive rate and symmetric distance for breakpoint identification, and a high CN-RMSE for CN calling (main the main text in Sections Performance of breakpoint detection and Performance of CN calling on page 9, also in Figure 3).*

*The impression that CONET does not clearly outperform other methods could have been due to three reasons:*

*CN-RMSE was minimized by CBS+MergeLevels.*

*In terms of breakpoint identification, for 4 out of 8 scenarios, CBS+MergeLevels has a slightly lower false negative rate and symmetric distance than CONET without known candidate breakpoint loci (now referred to as CONET (HMMCopy), as in this case the candidate breakpoints were found using HMMCopy).*

*There could be a misunderstanding, that the “known” versions of CONET or SCICoNE are given information about the presence or absence of the breakpoints in each cell.*

*Ad 1) The new results clearly show that the modification from taking mean to median counts over bins with the same CN event history improved CN-RMSE results for all versions of CONET. Moreover, CONET (CBS+MergeLevels) performs similarly well as CONET (known) in the tree inference (in the main text, new Figure 2). Here, it is most striking that CONET without known breakpoints outperforms SCICoNE with known breakpoints in most experimental setups and measures. Also, CONET (CBS+MergeLevels) obtains very low CN-RMSE, comparable to CBS+MergeLevels (in the main text, new Figure 3).*

*Ad 2) In terms of breakpoint identification, CONET (HMMCopy) has a clearly lower false positive rate in all 8 out of 8 scenarios and has only a slightly higher false negative rate and symmetric distance in 4 out of 8 scenarios. CONET (CBS+MergeLevels) outperforms CBS+MergeLevels itself in breakpoint identification for all scenarios and all measures (for 24 out of 24 measure-scenario combinations): false positive and negative rates, symmetric distance (in the main text, new Figure 3).*

*Ad 3) Neither known candidate breakpoint loci, nor candidate breakpoint loci found using either HMMCopy or CBS+MergeLevels, do not specify the presence or absence of breakpoints in the cells, but only the set of candidates for breakpoint loci (in the main text improved Figure 1A). The combined CONET tree inference and CN calling returns the identified tree, copy number profiles and the set of identified breakpoints (in the main text improved Figure 1C), which can be different from the input set of candidate breakpoints.*

*The extended results are now discussed in the main text in Section Performance of CONET in comparison to other methods on simulated data on pages 5-9. Altogether, this analysis clearly demonstrates that the joint proposed approach taken by CONET allows an improvement in terms of tree inference, breakpoint identification and copy number calling.*

---c. The proposed set of methods for the benchmark does not properly reflect current state of the art for the related field. In fact, some of the most established and important existing copy number calling methods are missing and should be included in the benchmark, especially Ginkgo [3], Aneufinder [4], and possibly some of the most recent methods and advancement like SCOPE [5]. Including some of these methods is critical to convincingly demonstrate that the joint approach proposed by CONET really has the potential to also improve copy number calling.

*Answer: To our understanding, the works that the Reviewer pointed, have a very different focus than CONET. CONET assumes preprocessed data at the input and the weight of the contribution is put on developing an advanced probabilistic model of an evolutionary tree over copy number events and simultaneous CN calling from the preprocessed data, together with an MCMC procedure for model inference. In contrast, for Gingko, Aneufinder, and SCOPE, most of the analysis is invested in pre-processing raw data, normalizing and correcting raw reads, while copy number calling, compared to CONET, is done in a straightforward manner. In contrast to HMMCopy and CBS+MergeLevels that we compare to, none of these three approaches is applicable to simulated data we used in our analysis. Therefore we conclude the comparison to these three methods is out of the scope of the presented work.*

*In fact, we consider avoiding the preprocessing step as an advantage, as the preprocessing pipeline should be suited for a particular sequencing technology. This makes CONET better generalizable, i.e. applicable to data coming from different sequencing protocols including those that will be developed in the future. This difference of CONET to other copy number approaches is now discussed in the main text in Section Discussion on page 18.*

3. Impact: the proposed model underlying CONET is based on two unrealistic assumptions on cancer biology that would substantially impact its potential impact.

---a. No cell ploidy estimation is performed in CONET, but rather cells are always assumed to have a based ploidy of two. However, aneuploidies and whole genome doublings are very frequent in cancer, and therefore ploidy estimation is one of the most important and challenging steps of copy number calling methods. Therefore, the underlying diploid assumption is not realistic and particularly concerning, and the lack of genome ploidy estimation substantially impacts the applicability of the proposed method.

*Answer: Although CONET does not estimate ploidy itself, it is applicable to data coming from tumors with a given basal ploidy different than two. We now demonstrate it by applying CONET to a triple-negative breast cancer (TN2 sample) generated by Minussi et al., (2021) using a novel ACT sequencing method, with a basal ploidy equal to three. We note here that to determine the ploidy for the TN2 sample, Minussi et al., (2021) did not analyze the same scDNA-seq ACT data, but used API fluorescence values from FACS. Such identified ploidy was provided as input to CONET.*

*The new ACT data analysis results are described in the main text in Section Application to scDNA-seq data of TN2 invasive ductal carcinoma sample on page 15 and illustrated in Additional File 1: new Figures S7 and S8. To avoid the misunderstanding that CONET is only applicable to basal ploidy of two, we now improved Section Methods in the main text to clarify that the basal ploidy is a parameter set by the user.*

---b. The underlying model of CONET is assuming that each copy number event occurs only once across different cells. However, the assumption does not seem to be particularly realistic especially when considering recurrent altered regions, like chromosomal arms and whole chromosomes; in fact, it is frequent in cancer to observe such subclonal events affecting the same chromosome multiple times in different cells within the same tumor.

*Answer: To clarify: CONET does not assume that each copy number event occurs only once across different cells. In contrast, regions recurrently altered across cells are likely to be inferred, as they are likely an effect of CN evolution. We do, however, assume that the occurrence of exactly the same event (with the same start and end) is not possible twice across the nodes of the tree. This would mean the occurrence of exactly the same CN event independently twice in the evolutionary history of that cell population.*

*In contrast to the SNV models, where only one SNV can be acquired in one event, here the event is defined by two breakpoints (start and end of a CN event). CONET allows multiple reappearances of any of those two breakpoints as a start or end of any other CN event across the tree. As such, CONET violates the so-called infinite sites assumption. In this way, CONET is more realistic, as it is known that the same breakpoint loci can be re-used for different events (for example a shorter and a longer one, with the same breakpoint start).*

---c. The impact of CONET in terms of biological applications has not been convincingly demonstrated. In fact, it is not clear if any of the proposed features of CONET are enabling the authors to identify new biology or new information from the previous data.

*Answer: CONET can identify new biology in several ways.*

*First, unlike other methods, CONET identifies new biology in the form of the evolutionary tree on CN events, giving the set of events and their partial order. This is the substantial new biology/new information that we bring compared to standard CN calling approaches. In terms of CN calling, we do not only provide integer copy number values across contiguous genomic regions for each cell, which are provided by other CN calling methods and constitute already a relevant biological insight. Instead, crucially, we provide such CN calls that are explained by the inferred tree.*

—Also, the results on the SA501X3F xenograft breast cancer sample do not seem to substantially differ from the input data and it would be critically important to compare it directly with the results that can be obtained using other existing methods, or even naïve approaches like simple rounding; specifically, can the authors show what the copy numbers inferred by other methods look like in a similar figure?

*Answer: As suggested by the Reviewer, in addition to other compared methods, we now also compare CONET to simple rounding (referred to as CC-rounding in the text). On simulated data, the CC-rounding was shown to clearly overfit and interpret noise as CN changes, which resulted in a very high false positive rate in the task of breakpoint identification and a high CN-RMSE (metrics shown in Additional File 1: Figure S2). CC-rounding performance metrics possible to calculate for the SA501X3F xenograft breast cancer sample (with unknown ground truth) also suggest strong overfitting (added in the main text in Table 1).*

*In addition, following the Reviewer’s suggestion, we include CN heatmaps for the SA501X3F xenograft breast cancer sample obtained using the methods (Additional File 1: Figures S3–CBS+MergeLevels and S4–CC-rounding), apart from HMMCopy, for which the heatmap is already presented in Zahn et al (2017) Figure 3, and SCIConE, for which the heatmap is already presented in Kuipers et al (2020, preprint) Figure 3. It is illuminating to contemplate the heatmap for the poor-performing naive CC-rounding (Additional File 1: Figure S4), which overfits to noise and is almost indistinguishable from the input per-bin data.*

*There is no ground truth for the real data, and thus we cannot say which copy number call is a true positive or negative. When evaluating the different methods, we expect and measure to what extent the obtained copy number calls are similar to the input data, but at the same time avoid falsely interpreting the high level of noise present in this input as actual CNs. Judging from the heatmaps and results in the main text in Table 1, all methods, except for the naive CC-rounding, achieve this to some extent, but at the same time show some differences in called copy numbers. Since in contrast to pure CN calling approaches, the events inferred by CONET are explained by the tree, they are less likely to be false positives. Also, their validation can indirectly be performed by validating the tree. The inferred tree for SA501X3F xenograft breast cancer sample shows plausible evolutionary events. In addition, as described in the main text in Section Additional validation of CONET using passage SA501X4F xenograft breast cancer sample on pages 15-16, we have validated it using an independent sample of single cells sequences from a passage (Additional File 1: Figure S6).*

—Moreover, CONET removed some events that seemed to be clearly present in small subgroups of cells (especially top part of the heatmap) while keeping other events present in similar numbers of cells; what are the differences between these events and was the choice strongly supported by the underlying data?

*Answer: We identify two main reasons why the model does not infer a CN. First, as explained above, the level of output detail is steered using regularization parameters. Also, the breakpoints for these events may not have high enough per-breakpoint absolute corrected count differences.*

MINOR COMMENTS.

1. The text mentions that corrections for GC and mappability biases are applied, but no detail is provided anywhere in the text. As there is not a unified correction for these biases, it is fundamental to report the related details.

*Answer: Data preprocessing is performed using existing approaches, prior to the application of CONET. We now provide an explanation of how GC and mappability biases were corrected for in the main text in Section Real data preprocessing on pages 20-21.*

2. A very important test is missing in the simulated benchmark: how does CONET's result compare to a stepwise approach in which copy numbers are first inferred (for example using CBS+Mergelevels) and then a tree is reconstructed based on the inferred copy numbers, for example using MEDICC [8] or similar methods?

*Answer: We did not identify an approach that could be applied to copy number calls to infer an evolutionary tree. MEDICC, in particular, is not applicable to such data. MEDICC is not specifically designed for scDNA-seq and not for many cells. For example, in the original paper the method is applied to only 15 genomes (the equivalent of 15 single cells) and 100 genomic segments with integer CN profiles, separately for major and minor alle. The information of minor or major alleles is not available for the simulated and real data that we analyze.*

3. In addition to SCICoNE, it would be important to discuss the differences between CONET and other recent copy number evolutionary models based on breakpoints, as the one proposed by Dorri et al (2020) [7]?

*Answer: We discuss differences to SCICoNE, Dorri et al (2020), and MEDALT in the main text in Section Discussion on page 18. We are not aware of any other approaches that would perform anything related to evolution on copy number events from scDNA-seq.*

4. An important question is the scalability of the proposed method, especially as it relies on a MCMC algorithm. Therefore, including simulations to explore this for varying numbers of cells would be very important, also considering the high number of cells in the most recent single cell DNA sequencing datasets (>2000-5000).

*Answer: Thank you for this suggestion. We comprehensively analyzed the scalability of CONET for large numbers of cells, evaluating its performance in the most challenging setting, for larger trees and noise. CONET performance is not negatively affected by the number of cells. The results are presented in Additional File 1: Table S1 and described in the main text in Section Performance of CONET in three additional challenging tasks on page 16.*

5. Main text is specifying that nodes of the trees correspond to events, but it does not look like to be the case from the reported figures, as every node seems to be defined by the combination of all the acquired events.

*Answer: As explained in the main text in the Section Model Overview on pages 3-4, CONET model infers trees with single event nodes. Since the inferred trees are large in the case of biological samples (225 nodes for the SA501X3F xenograft breast cancer sample), in Figure 4 we present the tree in a compacted form as is described in the Figure 4 caption. Specifically, we collapse nodes if they have no* cells attached *to them and do not have more than one child, facilitating a more compact visual representation. The number of collapsed CN events is printed at the beginning of the node’s label so that we do not lose any information about the original CONET.*

6. The exact definitions of infinite and finite site assumptions should be clearly stated to make the reading accessible to a broad audience.

*Answer: The infinite sites assumption is defined and explained in the main text in Section Background on page 2, while the finite sites assumption - in the main text in Section Model Overview on page 4.*

7. The colors of the same methods in the different result figures are inconsistent.

*Answer: Thank you for pointing that out. We have improved the colors of the methods to be consistent across different figures, including figures in the Additional File 1.*

8. Since HMMcopy is also inferring the ploidy of the different cells, it would be fairer in the simulations to fix the ploidy estimate in HMMcopy to the same value used by CONET.

*Answer: In all simulations we set the ploidy for HMMCopy to two.*

9. The exact motivation and need of the "count discrepancy penalty" is very unclear to the reviewer, despite its reported importance. A clarification and better explanations of this regularization would be particularly important.

*Answer: Intuitively, the count discrepancy penalty corrects discrepancies between the observed per-bin data and the inferred CNs across cells. We explain that intuition in the main text in Section Model overview on page 5.*

10. In the results for the SA501X3F xenograft breast cancer sample, CONET infers a tree in which nearly all the nodes only contain <5 cells. This result raises some concerns about the accuracy of the results: how likely is that there have been some many clonal expansions in the tumor, and we were so lucky to sample so many of them with such low numbers of cells?

*Answer: Cancer genomes are highly unstable and new mutations can arise with every cell division, so in fact every cell is different and single cell whole genome sequencing allows us to detect at least some of such differences. CNAs characteristic for a very small number of cells were also detected in the original publications ] for both SA501X3F (Zahn et al., 2017) and TN2 samples (Manussi et al., 2021). Thus, the number of assigned cells per each node as given by CONET is expected. Moreover, if the user only wants to infer CONET with events that are common for a higher number of cells, they can obtain a smaller tree by using regularization parameters (The role of regularization parameters is now better explained in the main text in a new Section Assessment of impact of the count discrepancy penalty on CONET performance based on the xenograft breast cancer SA501X3F data, on pages 17-18. It is also extensively studied in Additional File 1: Sections S5 and S6 and Figures S13 and S14. )*

11. In the real data results, one of the comparisons between the results of different methods is based on the differences from the corrected read counts and the inferred copy numbers (absolute differences). However, different methods have likely applied different corrections and normalizations, so it is not surprising that other methods have higher differences, as they have minimized different quantities. Therefore, this comparison is not accurate.

*Answer: All methods have been applied to data that was pre-processed (corrected and normalized) in the same way.*

REFERENCES:

[1] Kuipers, J., Tuncel, M. A., Ferreira, P., Jahn, K., & Beerenwinkel, N. (2020). Single-cell copy number calling and event history reconstruction. bioRxiv (https://doi.org/10.1101/2020.04.28.065755).

[2] Laks, E., McPherson, A., Zahn, H., Lai, D., Steif, A., Brimhall, J., ... & Shah, S. P. (2019). Clonal decomposition and DNA replication states defined by scaled single-cell genome sequencing. Cell, 179(5), 1207-1221.

[3] Garvin, T., Aboukhalil, R., Kendall, J., Baslan, T., Atwal, G. S., Hicks, J., ... & Schatz, M. C. (2015). Interactive analysis and assessment of single-cell copy-number variations. Nature methods, 12(11), 1058-1060.

[4] Bakker, B., Taudt, A., Belderbos, M. E., Porubsky, D., Spierings, D. C., de Jong, T. V., ... & Foijer, F. (2016). Single-cell sequencing reveals karyotype heterogeneity in murine and human malignancies. Genome biology, 17(1), 1-15.

[5] Wang, R., Lin, D. Y., & Jiang, Y. (2020). SCOPE: a normalization and copy-number estimation method for single-cell DNA sequencing. Cell systems, 10(5), 445-452.

[6] McPherson, A. W., Roth, A., Ha, G., Chauve, C., Steif, A., de Souza, C. P., ... & Shah, S. P. (2017). ReMixT: clone-specific genomic structure estimation in cancer. Genome biology, 18(1), 1-14.

[7] Dorri, F., Salehi, S., Chern, K., Funnell, T., Williams, M., Lai, D., Andronescu, M., Campbell, K.R., McPherson, A., Aparicio, S., Roth, A., Shah, S., Bouchard-Cˆot´e, A.: Efficient bayesian inference of phylogenetic trees from large scale, low-depth genome-wide single-cell data. bioRxiv (2020)

[8] Schwarz, R. F., Trinh, A., Sipos, B., Brenton, J. D., Goldman, N., & Markowetz, F. (2014). Phylogenetic quantification of intra-tumour heterogeneity. PLoS computational biology, 10(4), e1003535.

Reviewer #3:

This paper introduces CONET, a method for tree inference and copy-number calling from single-cell DNA-seq data of tumors. The paper includes a comparison against HMMcopy, CBS+MergeLevels and SCICoNE. Moreover, the paper shows the application of CONET to a DLP+ xenograft sample. I have the following comments.

1. Input to CONET does not resemble real data

—My first major concern is that CONET in its present form is not an independent method. Ideally, CONET would start from BAM files and produce the final output (tree, cell-to-clone clustering and CN matrix). However, the input that CONET requires are corrected read counts per bin, as well as a set of candidate breakpoints. For the real data set analyzed in this paper, the former are obtained from the DLP+ paper and the latter are obtained by running HMMcopy (segmentation method). This is not good -- to facilitate use of the method on other datasets, I suggest the authors include pre-processing scripts to obtain the CONET input.

*Answer: Indeed, CONET requires pre-processed data at input. Requiring pre-processed data and candidate variant sites at input is very common for phylogenetic approaches to SNV and CN evolution, also those applied to scDNA-seq data (refs [31-35 and 60,61,63] in the main text). The weight of our contribution is put on developing an advanced probabilistic model of an evolutionary tree over copy number events and simultaneous CN variant calling from the preprocessed data, together with a MCMC procedure for model inference.*

*In contrast, starting from BAM files and taking care of normalization and bias correction is in the focus of several copy number calling approaches, such as HMMCopy, CBS+MergeLevels, Gingko, Aneufinder, or SCOPE. For these approaches, most of the analysis is invested in pre-processing raw data, normalizing and correcting raw reads, while final copy number calling, compared to CONET, is done in a straightforward manner.*

*In fact, we consider avoiding the preprocessing step as an advantage, as the preprocessing pipeline should be suited for a particular sequencing technology. This makes CONET better generalizable, i.e., applicable to data coming from different sequencing protocols including those that will be developed in the future. This as well as the differences to copy number approaches that preprocess raw read data are now discussed in the main text in Section Discussion on page 18.*

—Moreover, the "corrected read counts" seem to essentially correspond to copy numbers. For example, Eq. (10) describes that inferred copy-number of a clone (i.e. group of cells) is simply the average input "corrected read count" of that bin among cells assigned to the clone. It is not clear to me how one would obtain such a quantity. Moreover, if it is indeed straightforward to infer "corrected read counts" from actual read counts then a simple baseline method would be to simply cluster the cells according to this quantity without using a tree.

*Answer: Thank you for raising this point. First, the "corrected read counts" (or the per-bin data) do not correspond to copy numbers. The corrected read count distribution is a mixture of (truncated) gaussians with large variances around the means that correspond to copy numbers. We now illustrate the data distribution in a new supplementary figure (Additional File 1: Figure S1). These distributions show the high level of noise in this data.*

*In fact, assuming that the corrected read counts correspond to copy numbers in a straightforward manner would lead to overfitting to noise. For simulated data, this problem is illustrated by the performance metrics of the naive approach of CN calling by rounding the per-bin counts to nearest integers (shortly CC-rounding; metrics added in Additional File 1: Figure S2). Recall the CN-RMSE measure, which can be interpreted as the difference between the inferred CNs and the true CNs. CC-rounding yields high CN-RMSE and calls false positive breakpoints resulting in dramatically high false positive rate for breakpoint identification.*

*For the SA501X3F xenograft breast cancer sample, it is instructive to contemplate the heatmap for naive CC-rounding (Additional File 1: Figure S4), which is almost indistinguishable from the input per-bin data. Performance metrics possible to calculate for biological sample (with unknown ground truth) also suggest strong overfitting (added in the main text in Table 1).*

*Second, what happens in equation (10) is a definition of such a set of bins from different cells, which underwent the same evolutionary history according to their attachment to inferred CONET. They are simply such bins that were changed by the same set of events on the way down from the root to the nodes to which their cells are attached. In Section CN calling in the main text, bins in this set are assigned an estimated integer copy number which is a median of the corrected counts across those bins. In this way, we utilize the evolutionary history encoded in the tree to direct the copy number calling (taking the median is an improvement with respect to the previous submission, where we were taking the mean). Note we do not cluster cells here, we “cluster” bins by their evolutionary history. Note that bins with the same history do not have to come from cells assigned to the same node in the tree. The procedure of clustering of bins and assigning them integer copy numbers is exemplified in improved Figure 1.*

— Line 28, p. 15: "corrected for GC content and other potential biases" -- please expand on this, discuss what biases might expect and how one can correct for this.

*Answer: The data pre-processing methods depend on a specific scDNA-seq technique and are not part of CONET model. Moreover we believe that new methods will be developed and they may need different corrections. This is the reason why we do not expand on this matter in the CONET - Copy Number Event Tree model Section in the main text.*

*The corrections applied to the data sets used in our Results (GC content and mappability correction for SA501X3F and SA501X4F samples sequenced using DLP technique and GC content correction for TN2 sample sequenced using ACT technique) are described in detail in Section Real data pre-processing on pages 20-21.*

2. CONET model

— I had a hard time understanding the CONET model. In particular, the manuscript uses "CV event" throughout the manuscript only formally introducing it for the first time in methods. Please provide a definition as soon as you use this term for the time in the Introduction.

*Answer: As suggested, the definition was added in the main text in Section Background, as soon as we use this term (on page 1). We also explain that we model CN events as pairs of breakpoints and that the CN events constitute the nodes of the tree (in Section Model overview beginning of page 4).*

— I suggest improving the presentation by including a graphical model (plate diagram). Right now, the method seems rather ad hoc with various penalties.

*Answer: As suggested by the Reviewer, the graphical representation of the model with priors and regularization based on the corrected count penalty is added to the overview Figure 1 in the main text.*

–- I am worried that the introduced tree model may lead to unrealistic inferences, as it does not incorporate practical evolutionary constraints regarding copy number. For example, what if you have a complete loss of a segment? The CONET model may amplify this segment later, something that cannot happen in practice.

*Answer: The model does not infer the copy number changes in the nodes of the tree. It only infers the pairs of breakpoints. If it did, in theory it would be possible, but in practice it is unlikely to happen - there is no evidence in the data for such events, as they are impossible, and the model will not infer something which it does not have evidence for.*

–- Good to provide an example figure, demonstrating I_bp, I_0, CN events, how one obtains CNs, etc.

*Answer: We now improved Figure 1, we explain that CN events are acquisitions of CNAs and in our model they are modelled as pairs of breakpoints and label the nodes of the tree, and we illustrate how CNs are found by showing clusters of bins with the same evolutionary history and their inferred CNs. I_bp and I_0 are more technical and we preferred to keep them in the Methods Section only. These variables are sets that are deterministically derived for a given cell, tree T and attachment \sigma; I_bp(j | T, \sigma) is a set of breakpoints that are present in cell j according to the tree and the attachment, while I_0(j | T, \sigma) is a set of breakpoints that are not present in cell j.*

— Considering recent work (CHISEL), I would appreciate a discussion of allele-specific copy numbers. Can the CONET model be extended to support this?

*Answer: Thank you for this suggestion. Possibly, CONET could be extended to account for allele-specific copy numbers in different clones using an approach similar to CHISEL. This is now discussed in the main text in Section Discussion on page 19.*

— Why normal distributions? Does this match real data?

*Answer: In the revised manuscript, we show that the assumed data distributions fit the real data perfectly, for datasets generated using two different technologies, DLP and ACT (Additional File 1: Figure S1). This is explicitly mentioned in the main text in Section Model Overview on page 5.*

— How to go from breakpoints to bins? Do you discard bins that fall within but do not overlap with two breakpoints? If not, do you merge bins between breakpoints into segments -- and if so, how do you account for differences in read count due to varying segment lengths?

*Answer: Each bin has a location and for each bin we can state its evolutionary history by checking which CN events changed it. A bin is considered changed by a CN event if this bin is localized between the start and end of this CN event. We do not discard any bins. We also do not explicitly merge bins between breakpoints into segments. As explained above and now visualized in Figure 1, we cluster bins that underwent the same history (were changed by the same set of CN events) and give them the same integer CN, which is a rounded median of their counts. Such clustered bins are often co-localized in the genome, and then they can create consistent segmens. Still, they do not have to form such segments, as for some cells they may be separated by bins from other clusters.*

3. Experimental evaluation

— Simulations are generated from same model used in the inference by CONET. In particular, the issue here is the use of "corrected read counts" -- per Major Comment 1, these are not read counts but rather reflect an estimate of integer copy number. It would be good if the simulated data closely resembled real data, to properly assess the performance of CONET and its generalizability to other datasets. I suggest using a simulator of read counts such as the one introduced in:

Mallory XF, Edrisi M, Navin N, Nakhleh L. Assessing the performance of methods for copy number aberration detection from single-cell DNA sequencing data. Ioshikhes I, ed. PLoS Comput Biol. 2020;16(7):e1008012. doi:10.1371/journal.pcbi.1008012

*Answer: Since our model takes as input not raw sequencing data, but data which is pre-processed (normalized and corrected for biases), we naturally also simulate such input data. The used data distributions are realistic, as they were carefully defined based on analysis of real sequencing data. In the revised manuscript, we show that the assumed data distributions fit the real data perfectly, for datasets generated using two different technologies, DLP and ACT (Additional File 1: Figure S1). This is mentioned in the main text in Section Model Overview on page 5.*

— Performance metrics should include assessment of cell clustering (such as Rand index, V-measure) as well as ordering of cells (ancestral pair recall, incomparable pair recall have been used in the literature for this.)

*Answer: CONET does not cluster cells, so we cannot assess the performance of CONET using clustering quality measures such as Rand index or V-measure. However, CONET orders CN events and since cells are assigned to nodes, we can assess whether the pairwise ordering of cells imposed by the partial order of their assigned nodes in the simulated data is correctly inferred by the method. To evaluate whether the descendant--ancestor relationship among cells has been correctly recovered by CONET, we defined and measured ancestry recall (defined in the main text in Section Model evaluation metrics for simulated data on page 33) in the challenging simulation setting with high noise (with tree size in {20, 40} and number of cells in {200, 1000}; Additional File 1: Figure S9). With average ancestry recall scores greater than 0.7 across all settings, and greater than 0.9 for CONET (known) in all scenarios, CONET proves to correctly recover ancestry relations of cells. This is now described in the main text in Section Performance of CONET in three additional challenging tasks on page 16.*

— How much is the tree helping? Following up on my first comment, the evaluation should include a comparison against a version of CONET without the tree, or a baseline method where one directly clusters "corrected read counts".

*Answer: As explained above, the tree, together with cell assignment to nodes, directs the identification of bin clusters, containing such bins that underwent the same CN events. This is now better visualized in Figure 1 with illustrated examples of such bin clusters and how the copy number is evaluated for them. There is no cell clustering performed by CONET and no version of the model without the tree. Instead, we compare our CN calling results, which arise from CONET structure and cell attachment, to those from other CN calling methods in Paragraph Comparison of CN calling results.*

- To show generalizability of the method, I suggest including additional real data experiments beyond the one DLP+ sample. For example, consider including 10X Genomics, ACT and standard DOP-PCR data.

*Answer: Thank you for this suggestion. In the revised manuscript we have applied CONET to the TN2 invasive ductal carcinoma sample with over 1000 single cells sequenced with ACT method. We present the results in the main text in Section Application to scDNA-seq data of TN2 invasive ductal carcinoma sample on page 15 and in Additional File 1: Figures S7 and S8.*

4. Code and usability

README.md on GitHub page does not include example usage, nor a description of the input and output formats, or instruction on how to run the executables. This makes using the method extremely hard.

*Answer: The example usage and description of the input were hidden further in the structure of our github repository - thank you for pointing that out. We have now expanded our initial readme.md file, in which we describe the model parameters, input and output formats, and point to the location of example usage python scripts. We have also added R repository which helps to visualize the output from the model and calculates the quality metrics (described in Additional File 1: Section S6.1) to better assess the quality of obtained results.*

5. Discussion of previous work in Introduction

Introduction does not adequately discuss previous work, including MEDALT, copy-number tree problems, CHISEL, copy-number calling in bulk DNA-seq data, etc.

*Answer: We have improved the presentation of previous work. MEDALT and other previous approaches that are somehow related to copy number evolution were already overviewed in the Background section, but the names of the methods were not mentioned. This is now corrected. Copy number tree problems approached by methods applied to bulk data are discussed in the second paragraph of the Background section. We extended the description of the general problem with modeling copy number evolution in the main text in Section Background pages 1-2. We discuss copy number calling together with identifying CN evolution from bulk data in the second paragraph of Background, page 2. We also now mention existing copy number calling methods applicable to scDNA-seq data, including CHISEL (page 2).*

Minor comments:

- Page 3, line 54: "defined by a tree" => "defined by a ROOTED tree".

*Answer: The correction was made as suggested.*

- Page 5, line 23 -- these are running times on what system?

*Answer: On a system equipped with AMD Ryzen Threadripper 3990X 64-Core CPU and 128 GB RAM, using 5 threads. This is now added to that line.*

- Eq. (1) -- very confusing condition (i - 1 >= 1). Why not write i >= 2.

*Answer: The correction was made as suggested.*

**Second round of review**

**Reviewer 1**

As before, I believe the work addresses an important problem in better resolving copy number evolution in tumors and offers promising and innovative ideas to solve this problem. I provide my other comments in response to the Review Questions. I do not have any further concerns aside from a minor typo I note there.

I found the revisions and response to reviewers well responsive to the critiques and believe all of my concerns have been sufficiently addressed. Minor errors have been corrected, some points of confusion about the method and validation protocols have been clarified, and the empirical evidence is greatly improved that the method is an improvement over state-of-the-art alternatives that can offer novel insight into tumor biology. I have also considered the concerns of the other reviewers and while I largely agree with their earlier concerns, I would consider the points they raised answered to my satisfaction. I believe the revised manuscript does make a good case that the work is significant for the area, technically innovative, and sufficiently validated to show a valuable advance over the prior art.

I have no other critiques to offer, aside from one minor typo I noticed: ``settenig’’ on page 17.

**Reviewer 2**

The authors have added new descriptions, experiments, and figures which help address some of the previous technical issues and concerns. However, the concerns related to the motivation, novelty, impact, and benchmarking of the proposed contribution have not been satisfactorily addressed. Thus, the manuscript still does not convincingly demonstrate that the new proposed algorithm can have a substantial impact on the biological application and remains a more technical and incremental contribution which requires a more comprehensive benchmark (some of the proposed methods have been erroneously excluded).

Detailed comments of major concerns:

Motivation: it remains unclear whether the proposed algorithm CONET is a copy number calling method (meaning a method that infers integer copy numbers of genomic segments from single cell DNA sequencing data). On the one hand, the goal of CONET seems to be the inference of integer copy numbers and, in fact, CONET’s results are compared with those of other well-known copy number calling methods (such as HMMcopy and CBS+MergeLevels). On the other hand, CONET requires in input pre-processed data that have to be inferred by other copy number callers, especially candidate breakpoints (for example inferred by HMMcopy), cell ploidies (inferred by other methods or measured experimentally by FACS), and corrected read counts (which are essentially point estimates of integer copy numbers, obtained by scaling raw read counts by ploidy). As pointed out by all Reviewers, these limitations significantly restrict the applicability of CONET which, therefore, does not represent a proper copy number calling method and it cannot be an independent alternative to existing methods. Maybe, CONET could represent a method to correct/improve copy numbers inferred by other existing methods, but the manuscript should be restructured to be put in this context and targeted experiments to prove the importance of correcting inferred copy numbers should be performed. Moreover, if CONET is a method to correct inferred copy numbers, why not starting from the integer copy numbers already inferred by other methods? It is unclear why CONET should take in input the breakpoints inferred by other methods (like HMMcopy) but not the inferred integer copy numbers.

Innovation: The innovations of using both per-bin and per-breakpoint data have been overstated and should be properly put into the context of current literature. In fact, nearly all existing copy number calling methods based on genome segmentation already use per-breakpoint and per-bin data. For example, HMMcopy uses per-breakpoint data through a HMM algorithm to identify change points in the genome and it uses per-bin data to infer the underlying integer copy numbers by properly scaling the related raw read counts. The fact that CONET uses a different mathematical definition of per-breakpoint data is more a technical difference rather than methodological innovation.

Simulations: The new Figure S1 does not demonstrate that the proposed simulations are realistic, but it simply shows that there exists a fit of the proposed underlying probabilistic model for two real datasets. Why should we expect a model of arbitrarily high complexity to not fit the data? Rather than comparing the fitness of the probabilistic model, it would be important and more interesting to compare the distributions of read counts in the simulated datasets vs those in the real datasets. Yet, the most accurate and convincing benchmark would be obtained by simulating single cell DNA sequencing reads using one of the main available alternatives, from which the input data required by CONET could be easily obtained.

Benchmark: Importantly, a comparison with existing methods for reconstructing copy number trees from single cells has to be included, especially considering methods like MEDICC (https://doi.org/10.1101/2021.02.28.433227 and https://doi.org/10.1371/journal.pcbi.1003535) WCND (https://doi.org/10.1093/bioinformatics/btaa470), Sitka Tree (https://doi.org/10.1101/2020.05.06.058180) and MEDALT (https://doi.org/10.1186/s13059-021-02291-5). The authors have claimed that no methods exist to do this, but these methods above demonstrate otherwise. In particular, the authors have claimed that MEDICC cannot be applied to many single cells, but this is also incorrect since the recent version of MEDICC2 has been applied to thousands of single cells (see https://doi.org/10.1101/2021.02.28.433227). Moreover, two important concerns still remain in the benchmarking results. First, existing copy number calling methods (CBS+MergeLevels) seem to still perform equally or better than CONET in several metrics (Fig. 2G,K,O,P), remarking that it would be important to expand the benchmarking to include more and the most recent copy number calling methods, especially the standard Aneufinder and the recent SCOPE, which has been applied to very similar datasets. The authors have claimed that Aneufinder, Ginkgo, and SCOPE are out of scope for the proposed comparison, but this is clearly incorrect since together with HMMcopy (included in the comparison) these methods are among the most common existing copy number calling methods and are indeed included in state of the art benchmarks (https://doi.org/10.1371/journal.pcbi.1008012 and https://doi.org/10.1186/s13059-020-02119-8). Second, HMMcopy seems to be performing particularly badly on simulations, despite being one of the most used methods, how is this explained?

Impact: The results of CONET on real datasets do not demonstrate the potential to reveal novel biological insights compared to existing methods. While it is possible that some of the novel features of CONET could allow the identification of new biology, this possibility should be demonstrated on the presented results. Moreover, placing copy number events on a tree does not make the results more reliable per se (“the events inferred by CONET are explained by the tree, they are less likely to be false positives”) and at least some orthogonal evidence should be provided to indicate higher accuracy of CONET’s results.

**Reviewer 3**

The revised manuscript has addressed most of my previous comments. I appreciate the additional real data experiments, as well as the detailed descriptions on data processing. Three minor comments left:

1. Regarding my previous comment about cell clustering, the cell attachment function \sigma : {1,...,m} -> V_T is assigning multiple cells to the same vertex in the tree — and thus can be viewed as cell clustering. It would be good to assess accuracy of this function on simulations.

2. The revised manuscript now assesses "ancestry recall”, which is good. To complete assessment of cell attachments, I encourage the authors to also include “branching recall” in the manuscript. The fraction of pairs of cells that were attached to distinct branches in ground truth that are recalled.

3. Finally, regarding the new TN2 (ACT) data analysis, the manuscript states:

“The sequencing reads were binned into bins with varying width (average 222349 bases) and corrected for GC content.”

Which tools were used for doing this? More details would be appreciated. Also, it is becoming more common in the field to release processing pipelines on github to facilitate reproduction of the results (e.g. using snakemake). I think this would be a good idea in general for all results here, especially given the many preprocessing steps required to obtain the input to the tool.

**Authors Response**

**Point-by-point responses to the reviewers’ comments:**

Reviewer #1:

As before, I believe the work addresses an important problem in better resolving copy number evolution in tumors and offers promising and innovative ideas to solve this problem. I provide my other comments in response to the Review Questions. I do not have any further concerns aside from a minor typo I note there.

I found the revisions and response to reviewers well responsive to the critiques and believe all of my concerns have been sufficiently addressed. Minor errors have been corrected, some points of confusion about the method and validation protocols have been clarified, and the empirical evidence is greatly improved that the method is an improvement over state-of-the-art alternatives that can offer novel insight into tumor biology. I have also considered the concerns of the other reviewers and while I largely agree with their earlier concerns, I would consider the points they raised answered to my satisfaction. I believe the revised manuscript does make a good case that the work is significant for the area, technically innovative, and sufficiently validated to show a valuable advance over the prior art.

I have no other critiques to offer, aside from one minor typo I noticed: ``settenig’’ on page 17.

*ANSWER: Thank you very much for the favourable opinion. We corrected the typo on page 17 (now 14 - Figures removed from main body of the manuscript).*

Reviewer #2:

The authors have added new descriptions, experiments, and figures which help address some of the previous technical issues and concerns. However, the concerns related to the motivation, novelty, impact, and benchmarking of the proposed contribution have not been satisfactorily addressed. Thus, the manuscript still does not convincingly demonstrate that the new proposed algorithm can have a substantial impact on the biological application and remains a more technical and incremental contribution which requires a more comprehensive benchmark (some of the proposed methods have been erroneously excluded).

Detailed comments of major concerns:

Motivation: it remains unclear whether the proposed algorithm CONET is a copy number calling method (meaning a method that infers integer copy numbers of genomic segments from single cell DNA sequencing data). On the one hand, the goal of CONET seems to be the inference of integer copy numbers and, in fact, CONET’s results are compared with those of other well-known copy number calling methods (such as HMMcopy and CBS+MergeLevels). On the other hand, CONET requires in input pre-processed data that have to be inferred by other copy number callers, especially candidate breakpoints (for example inferred by HMMcopy), cell ploidies (inferred by other methods or measured experimentally by FACS), and corrected read counts (which are essentially point estimates of integer copy numbers, obtained by scaling raw read counts by ploidy). As pointed out by all Reviewers, these limitations significantly restrict the applicability of CONET which, therefore, does not represent a proper copy number calling method and it cannot be an independent alternative to existing methods. Maybe, CONET could represent a method to correct/improve copy numbers inferred by other existing methods, but the manuscript should be restructured to be put in this context and targeted experiments to prove the importance of correcting inferred copy numbers should be performed. Moreover, if CONET is a method to correct inferred copy numbers, why not starting from the integer copy numbers already inferred by other methods? It is unclear why CONET should take in input the breakpoints inferred by other methods (like HMMcopy) but not the inferred integer copy numbers.

*ANSWER: Thank you for your detailed remarks. Indeed, CONET is not only a copy number calling method but - as stated in the abstract, the last paragraph of the background (on pages 2-3), Figure 1 and in several other places in the manuscript - it is a method of both reconstructing a CN event tree, and CN calling. It has the advantage over existing CN calling methods because it explains how the final CN profiles arise in cells by recreating the order of CN events and placing them on an evolutionary tree. Thus it gives additional biological insights into the importance of CN events changing the number of cancer genes copies in cells, by looking into the tree structure (described in for example in Section CONET structure for SA501X3F xenograft breast cancer data and shown in Figure 4). CONET CN profiles result from the structure of CN event tree and thus reflect an evolutionary process. Their comparison to CN profiles inferred by other CN calling methods is included to demonstrate the relevance of our approach.*

*Regarding the necessity of using other methods to obtain the set of candidate breakpoints: since the focus and main innovation of CONET is inferring the tree structure of CN event tree, we have left the opportunity to establish the set of potential breakpoints to the user, who can freely choose between existing methods and add their own candidate breakpoints. During the CONET modelling procedure, the candidate breakpoints are assessed by the model (some of them may be left out and not included as breakpoints in the final tree and CN calling).*

Innovation: The innovations of using both per-bin and per-breakpoint data have been overstated and should be properly put into the context of current literature. In fact, nearly all existing copy number calling methods based on genome segmentation already use per-breakpoint and per-bin data. For example, HMMcopy uses per-breakpoint data through a HMM algorithm to identify change points in the genome and it uses per-bin data to infer the underlying integer copy numbers by properly scaling the related raw read counts. The fact that CONET uses a different mathematical definition of per-breakpoint data is more a technical difference rather than methodological innovation.

*Answer: We are very grateful for pointing that out. We modified the manuscript in parts referring to the per-bin and per-breakpoint data not to overstate the novelty of using both data representations. Specifically, we removed the mention of per-bin and per-breakpoint data from the Abstract completely, we removed the paragraph introducing the per-bin and per-breakpoint data as a separate paragraph in the Background and restructured the next paragraph so that it mentions the use of both data types as a feature of the approach, without underlining its novelty (page 3). In the Discussion, we removed a sentence mentioning per bin and per breakpoint data from the first paragraph on page 15, and changed text on page 16. Finally, we added an explanation about similarities and differences between the ways some CN calling methods and CONETexploring the per-bin and per-breakpoint signal in the data on page 15. There is no mention of using both per-bin and per-breakpoint data as a novelty across the entire manuscript.*

Simulations: The new Figure S1 does not demonstrate that the proposed simulations are realistic, but it simply shows that there exists a fit of the proposed underlying probabilistic model for two real datasets. Why should we expect a model of arbitrarily high complexity to not fit the data? Rather than comparing the fitness of the probabilistic model, it would be important and more interesting to compare the distributions of read counts in the simulated datasets vs those in the real datasets. Yet, the most accurate and convincing benchmark would be obtained by simulating single cell DNA sequencing reads using one of the main available alternatives, from which the input data required by CONET could be easily obtained.

*Answer: The real data in Figure S1 are fit to a mixed normal distribution with five components, which is not of arbitrarily high complexity. Exactly such fitted mixed normal distribution of the same complexity is used to simulate the synthetic data. Specifically, (as described in Section: Simulations for comparative evaluation of CONET on both per-bin and per-breakpoint data on page 28) in order to obtain a realistic distribution of simulated corrected counts, we fit a mixture of five normal distributions with means fixed to (0,1,2,3,4) and unknown variances to the distribution of corrected counts from the xenograft breast cancer SA501X3F data.*

Benchmark: Importantly, a comparison with existing methods for reconstructing copy number trees from single cells has to be included, especially considering methods like MEDICC (https://doi.org/10.1101/2021.02.28.433227 and https://doi.org/10.1371/journal.pcbi.1003535) WCND (https://doi.org/10.1093/bioinformatics/btaa470), Sitka Tree (https://doi.org/10.1101/2020.05.06.058180) and MEDALT (https://doi.org/10.1186/s13059-021-02291-5). The authors have claimed that no methods exist to do this, but these methods above demonstrate otherwise. In particular, the authors have claimed that MEDICC cannot be applied to many single cells, but this is also incorrect since the recent version of MEDICC2 has been applied to thousands of single cells (see https://doi.org/10.1101/2021.02.28.433227). Moreover, two important concerns still remain in the benchmarking results. First, existing copy number calling methods (CBS+MergeLevels) seem to still perform equally or better than CONET in

several metrics (Fig. 2G,K,O,P), remarking that it would be important to expand the benchmarking to include more and the most recent copy number calling methods, especially the standard Aneufinder and the recent SCOPE, which has been applied to very similar datasets. The authors have claimed that Aneufinder, Ginkgo, and SCOPE are out of scope for the proposed comparison, but this is clearly incorrect since together with HMMcopy (included in the comparison) these methods are among the most common existing copy number calling methods and are indeed included in state of the art benchmarks (https://doi.org/10.1371/journal.pcbi.1008012 and https://doi.org/10.1186/s13059-020-02119-8). Second, HMMcopy seems to be performing particularly badly on simulations, despite being one of the most used methods, how is this explained?

*Answer: Thank you for this thorough analysis. Regarding the first part, we have included a comparison to CN tree reconstruction for SCICoNE model (Kuipers, which is still in preprint) and for MEDALT (Wang2021, published) which is inferring cell lineage tree (see Section: Comparison of event tree inference on pages 10-11). MEDICC2 and sitka model are still preprints (indicated in Section: Background on page 2), and sitka model infers only breakpoint tree (not CN event tree). As for benchmarking the CN calling methods - we have presented those that could have been applied to our synthetic data (many of the mentioned methods require different data at input).*

*Regarding the second part of your comment, indeed HMMCopy performs badly compared to other methods and it may be explained by the fact that all methods, including HMMCopy were run using their default settings. Tweaking their parameters potentially could improve their results. We now state it directly in Section: Performance of breakpoint detection on page 7 and in Section: Performance of CN calling on page 7.*

Impact: The results of CONET on real datasets do not demonstrate the potential to reveal novel biological insights compared to existing methods. While it is possible that some of the novel features of CONET could allow the identification of new biology, this possibility should be demonstrated on the presented results. Moreover, placing copy number events on a tree does not make the results more reliable per se (“the events inferred by CONET are explained by the tree, they are less likely to be false positives”) and at least some orthogonal evidence should be provided to indicate higher accuracy of CONET’s results.

*Answer: Please see above the answer to the Motivation part of the review. In addition, we would like to emphasize that apart from only evaluating CN calling itself (Figures 3 and 5, Table 1, Supplementary Figure S8, Supplementary Table S4), we also included available orthogonal evidence, such as i) multiple evaluations of the quality of breakpoint detection, tree inference, cell attachment, cell ancestry and cell clustering (Figures 2 and 4, Supplementary Figures S7, S9, S13 and S14, Supplementary Tables S1 and S3), which validate the inferred tree and indirectly the CN calling made based on the tree, and ii) validation on additional passage of the DLP breast cancer data SA501X4F, which showed results expected biologically (Supplementary Figure S6).*

Reviewer #3:

The revised manuscript has addressed most of my previous comments. I appreciate the additional real data experiments, as well as the detailed descriptions on data processing. Three minor comments left:

1. Regarding my previous comment about cell clustering, the cell attachment function \sigma : {1,...,m} -> V_T is assigning multiple cells to the same vertex in the tree — and thus can be viewed as cell clustering. It would be good to assess accuracy of this function on simulations.

*Answer: Thank you for pointing that out. To assess the quality of such defined clustering, we computed the rand index (described in the main text in Section Model evaluation metrics for simulated data on page 30) measuring the agreement of the true clustering of cells to the vertices with the inferred clustering, in the challenging simulation setting with high noise (with tree size in {20, 40} and number of cells in {200, 1000}; Additional File 1: Figure S9 C, D - extended). With a median rand index greater than 0.9 across all settings, CONET demonstrates excellent performance in clustering the cells by attaching them to tree vertices. This is now described in the main text in Section Performance of CONET in three additional challenging tasks on pages 12-13.*

2. The revised manuscript now assesses "ancestry recall”, which is good. To complete assessment of cell attachments, I encourage the authors to also include “branching recall” in the manuscript. The fraction of pairs of cells that were attached to distinct branches in ground truth that are recalled.

*Answer: As suggested by the Reviewer, we computed the branching recall (described in the main text in Section Model evaluation metrics for simulated data on page 30) in the challenging simulation setting with high noise, same as for the ancestry recall and rand index (with tree size in {20, 40} and number of cells in {200, 1000}; Additional File 1: Figure S9 E, F - extended). With median branching recall scores greater than 0.7 across all settings, CONET proves to correctly recover the branching of cells. This is now described in the main text in Section Performance of CONET in three additional challenging tasks on pages 12-13.*

3. Finally, regarding the new TN2 (ACT) data analysis, the manuscript states:

“The sequencing reads were binned into bins with varying width (average 222349 bases) and corrected for GC content.”

Which tools were used for doing this? More details would be appreciated. Also, it is becoming more common in the field to release processing pipelines on github to facilitate reproduction of the results (e.g. using snakemake). I think this would be a good idea in general for all results here, especially given the many preprocessing steps required to obtain the input to the tool.

*Answer: The data was preprocessed by Minussi et al 2021 as described in that paper. We received the already preprocessed data from the authors of Minussi 2021 and we now state it directly in the manuscript in Section: Real data pre-processing on page 18.*

**Third round of review**

**Reviewer 3**

The authors have addressed my previous comments satisfactorily. I have no further comments.
